# Whole Genome Scan and Selection Signatures for Climate Adaption in Yanbian Cattle

**DOI:** 10.3389/fgene.2020.00094

**Published:** 2020-02-25

**Authors:** Jiafei Shen, Quratulain Hanif, Yang Cao, Yongsheng Yu, Chuzhao Lei, Guoliang Zhang, Yumin Zhao

**Affiliations:** ^1^ Key Laboratory of Beef Cattle Genetics and Breeding in Ministry of Agriculture and Rural Agriculture, Branch of Animal Husbandry, Jilin Academy of Agricultural Sciences, Changchun, China; ^2^ College of Animal Science and Technology, Northwest A&F University, Yangling, China; ^3^ Computational Biology Laboratory, Department of Agricutural Biotechnology, National Institute for Biotechnology and Genetic Engineering, Faisalabad, Pakistan; ^4^ Pakistan Institute of Engineering and Applied Sciences, Nilore, Pakistan

**Keywords:** cold adaptability, positive selective, *CD36*, *CORT*, *FGF5*

## Abstract

Yanbian cattle is inhabitant of North of China, exhibiting many phenotypic features, such as long, dense body hair, and abundant intramuscular fat, designed to combat the extreme cold climate adaption. In the current study, we studied the cold tolerance of nine Yanbian cattle by whole genome resequencing and compared with African tropical cattle, N’Dama, as a control group. Yanbian cattle was aligned to the Bos taurus reference genome (ARS-UCD1.2) yielding an average of 10.8 fold coverage. The positive selective sweep analysis for the cold adaption in Yanbian cattle were analyzed using composite likelihood ratio (CLR) and nucleotide diversity (θπ), resulting in 292 overlapped genes. The strongest selective signal was found on BTA16 with potential mutation in CORT gene, a regulatory gene of primary hormone in the hypothalamic-pituitary-adrenal (HPA) axis, is reported to be associated with the cold stress, representedfour missense mutations (c.269C > T, p.Lys90Ile; c.251A > G, p.Glu84Gly; c.112C > T, p.Pro38Ser; c.86G > A, p.Pro29His). Meanwhile another gene on BTA6, showed significantly higher selective sweep signals for a cold adapted trait for hair follicle and length development, *FGF5* (fibroblast growth factor 5) with a missense mutation (c.191C > T, p.Ser64Phe). Moreover, cold adapted Yanbian cattle was statistically compared with the hot adapted N’Dama cattle, a taurine cattle reported to show superior heat tolerance than zebu cattle, making them better adapted to the hot regions of Africa. XP-CLR, Fst, and θπ ratio were used to compare both breeds, yielding 487, 924, and 346 genes respectively. Among the 12 overlapped genes, (*CD36*) (c.638A > G, p.Lys 213Arg) involved in fat digestion and absorption plays an important role in membrane transport of long-chain fatty acid and its expression could increase in cold exposure. Henceforth, our study provides a novel genetic insights into the cold climate adaptation of Yanbian cattle and identified three candidate genes (*CORT*, *FGF5*, *and CD36*), which can add to an understanding of the cold climate adaptation of Yanbian cattle.

## Introduction

Cold climate adaptation is a general term used to describe the physiological functions associated with cold adaptation. Studies show that cold exposure will lead to increase the blood pressure (De Lorenzo et al., 1999). During cold stress, the body tries to save energy by cheap methods (such as standing hair) and by changes posture to reduce surface area (Cannon and Nedergaard, 2004). To protect tissues from cold damage, the body adopts different processes, which increases warm blood flow near the surface of the skin (Cannon and Nedergaard, 2011). Those adaptation mechanisms, as well as biological processes, suggest the complex mechanisms of adaptation to cold.

Yanbian cattle is a taurine breed that living in northeast China (Xin et al., 2010) and belongs to the “yellow” class of Chinese cattle (Ji et al., 2014). Unlike majority of Chinese indigenous breeds, Yanbian cattle have had no ancestral to breed with indicine cattle (Xin et al., 2014). They are mainly used as herbivores, especially in the rice fields but are also increasingly used for the beef purpose. The living environment of Yanbian cattle has long, freezing winters with snow-covered grounds half a year and only brief summers. The temperature drops as low as −37°C at the peak of the winter season (**Figure S1**). Yanbian cattle exhibit unique morphoanatomical adaptations to the cold climate with its long and dense hairs as predicted by Allen’s rule (Allen, 1877), compactly built with short limbs. On the other hand, N’Dama has been known for its heat resistance in the harsh climatic conditions in Africa. The temperature rise as high as 50°C in the summers, while the weather remains hotter rest of the year as well. Thus, it is an ideal taurine to be compared with Yanbian cattle to identify the potential temperature regulating genes and pathways.

Along with constant release of whole-genomic sequence data in domesticated cattle (Daetwyler et al., 2014; Stothard et al., 2015; Kim et al., 2017), continuous expansion of directory to genetic variants (Karim et al., 2011), gradual maturity of selective theory and method (Vitti et al., 2013), the genetic basis of phenotypic diversity can be hunted down at the complete genome level. However, to our knowledge, no information has yet been generated regarding the cold climate adaptation based on whole genomes level in Yanbian cattle.

Yanbian cattle living in cold environments can be an excellent model for the identification of genomic loci explaining cold climate adaptation in cattle. Here, we are starting from the whole genome scan of Yanbian and N’Dama cattle, and reported genes that are positively selected in Yanbian cattle associated with cold stress adaptation. We used composite likelihood ratio (CLR) and θπ statistics to study the diverse nature of Yanbian cattle, which can provide genomic materials for genetic improvement of Yanbian cattle adaptive traits. Furthermore, we also employed three different statistical approaches i.e., XP-CLR, Fst, and *θ*π ratio, in order to detect selection signatures in Yanbian cattle, compared to N’Dama cattle. The high fat content, marbling, and the superior hide of Yanbian cattle needs to be well preserved and further enhanced. However, the genetic predispositions associated with adaption and enhanced cold tolerant parameters remain uncertain. The current study will help us to enunciate the extreme environmental adaptations and the positive selective sweeps in the Yanbian cattle.

## Material and Methods

### Library Construction and Sequencing

We generated whole-genome resequencing data for nine Yanbian cattle, three of them have been generated from our previous study (Chen et al., 2018). N’Dama cattle (n=10), a natural inhabitant of temporal climate, was included in the study for the genome comparison, in order to understand the heat and cold tolerance in livestock (Kim et al., 2017). The genomic DNA was extracted from the ear tissues using the standard phenol-chloroform protocol (Sterky et al., 2017). In **Supplementary Note 1** we have detailed description about the approaches and tools utilized in the current analysis.

### Read Mapping and Single-Nucleotide Polymorphism Calling

The clean reads were aligned to the latest reference genome sequence (GCF_002263795.1) using Burrows-Wheeler Aligner (BWA)-MEM (Li et al., 2009) with default parameters. The Picard tools (version 1.106) were used to generate the quality matrices whereas, the Genome Analysis Toolkit (GATK, version 3.8) was employed for the single-nucleotide polymorphism (SNP) calling for mapping (McKenna et al., 2010). We used “HaplotypeCaller,” “GenotypeGVCFs,” and “SelectVariants” argument of GATK to call the raw SNP. The filtration of the raw SNPs was conducted by using “variant Filtration” with the following parameters: 1) the depth of base quality to ensure variant confidence (QD) < 2.0; 2) the quality of mapping reads (MQ) > 40.0; 3) also, the Phred-scaled P-value calculating with Fisher`s exact test (FS) < 60.0; 4) ReadPosRankSum < −8.0, 5) MQRankSum < −12.5; 6) mean sequence depth (for all individuals) > 1/3× and < 3×; while, 7) SOR > 3.0.

### Variant Functional Annotation and Enrichment Analysis

The SNP were annotated by SnpEff tool using ARS-UCD1.2 database and gene set enrichment analyses were carried out with Gene Ontology (GO) and Kyoto Encyclopedia of Genes and Genomes (KEGG) pathway using KEGG Orthology-Based Annotation System (KOBAS) tool. To provide a preliminary overview of the genomic excess and test its reliability, we performed different GO and KEGG pathway enrichment analyses with KOBAS using different lists of genes located in chromosomal regions from the different selective analysis methods of Yanbian to N’Dama cattle. The enriched pathways and genes were selected stringently with an adjusted probability (*P* < 0.05).

### Selective Sweep Identification

The genome and nucleotide diversity were calculated by *θ*π, whereas, allele frequency for the positive selection signals were attained by CLR in Yanbian cattle. To infer the scan in progress, we used a 50 kb window for both statistical parameters. The CLR test uses information from allele frequencies to detect selective scans and relies on determining skews in the allele spectrum to bias rare and frequent alleles (Nielsen et al., 2005). Whereas, *θ*π analyzes the complete polymorphism data to compile the nature of diversity of the species.

We also performed XP-CLR (Chen et al., 2010), Fst, and *θ*π ratio to identify the potential areas differentially, selected between Yanbian and N’Dama cattle. XP-CLR is used for detecting selective sweeps that models the multilocus allele frequency differentiation between the two populations (Chen et al., 2010). We used a 50 kb non-overlapping sliding windows with the number of SNPs less than 600, and the correlation level reduced the contribution of SNPs to the XP-CLR results to 0.95. XP-CLR values in the top 0.5% of the empirical distribution (XP-CLR> 29.49) are designated as candidate selection scans, and genes that in those window region are defined as potential candidate genes (Lee et al., 2014). It will give more evidence to the same gene regions using different methods (Qanbari and Simianer, 2014). Fst can change the degree of differentiation between the different populations of one species. If populations are differentiated, the number of genetic differentiation in the selected locus region increase while the difference in genomic region is greater than the neutral conditions (Oleksyk et al., 2010). VCFtools were used to calculate the Fst values of the candidate gene regions in a 50 kb window size at an interval of 20 kb steps (Danecek et al., 2011). Finally, the identified selective sweeps regions were annotated to the reference genome (ARS-UCD1.2) and were further explored following the functional analysis. Lastly, *θ*π ratio between Yanbian and N’Dama cattle groups was calculated as ln(θπ,Yanbian/θπ,N’Dama), which reflected the loss of nucleotide diversity in Yanbian cattle relative to N’Dama.

## Results

### Resequencing of Yanbian Cattle and Single-Nucleotide Polymorphism Call

The re-sequenced Yanbian and the N’Dama cattle were pooled together in a 19 cattle data set, generated to an average of 9.15X coverage. In total, 4.2 billion reads were generated which were aligned against the ARS-UCD1.2 reference genome using BWA MEM algorithm, yielding ~4.1 billion mapping reads, covering 99.16% of the reference sequence across the region (**Table 1**). After the SNP call, 11,331,903 and 5,720,198 SNPs in Yanbian and N’Dama cattle, respectively were identified. The quality of the SNP call was evaluated with the Ts/Tv ratio (**Table 2**). The SNPs count of Yanbian cattle was observed to be larger than N’Dama, which might account for the low coverage and low genetic diversity of N’Dama sequencing. At the same time, the average ratios of homozygous versus heterozygous SNPs of Yanbian and N’Dama cattle are 0.487 and 0.848, respectively, representing the more genetic diversity in North China with its elevated heterozygous.

**Table 1:** Summary statistics of Yanbian and N’Dama cattle re-sequenced reads.

Sample name	No. sample	Raw reads	Mapped reads	^a^Properly paired reads	^b^Average coverage	^c^Average fold
Yanbian	9	2,722,889,367	2,716,850,732	2,691,580,076	99.78%	10.8X
N’Dama	10	1,474,655,523	1,453,725,812	1,424,295,346	98.54%	7.5X
Total	19	4,197,544,890	4,170,576,544	4,115,875,422	99.16%	9.15X

a Properly paired reads, “properly paired” means that both ends of the reads were mapped with correct orientation and their fragment sizes were less than 500 bp.

b Average coverage, assembly coverage calculated as the proportion of bases in the genome assembly that were covered by at least one read.

c Average fold, average fold that was calculated as the average depth of coverage across the whole genome.

**Table 2:** Functional annotation of the identified single-nucleotide polymorphisms (SNPs) in Yanbian and N’Dama cattle.

Fields		Yanbian	N’Dama	Total
**Sample counts**		9	10	19
^a^SNP counts		11,331,903	5,720,198	12,246,286
^b^Ts/Tv ratio		2.3604	2.3969	***
Hom/Het ratio		0.467871422	0.848630285	***
^c^**SNP categories**				
^d^Exon	Synonymous variant	186,012	194,636	314,721
	Initiator codon variant	16	13	21
	Start lost	183	97	237
	Stop gained	858	526	1,151
	Stop lost	167	81	182
	Stop retained variant	150	81	165
Splice	Splice region variant	37,336	21,114	45,471
site	Splice acceptor variant	484	359	613
	Splice donor variant	610	311	723
Intron	Intron variant	48,543,638	27,356,467	55,275,820
	Intragenic variant	7,816,671	4,242,273	8,544,070
UTR	5 prime UTR variant	57,009	36,103	72,734
	5 prime UTR premature-start codon gain variant	9,935	6,167	12,529
	3 prime UTR variant	243,890	122,412	279,308
Intergenic	^e^Upstream gene variant	2,709,568	1,467,399	3,120,061
	^e^Downstream gene variant	2,787,820	1,487,654	3,198,705
	Intergenic region	6,128,216	3,047,398	6,503,198
**Functional classes**				
	Missense	110,574	78,703	151,271
	Nonsense	858	526	1,151
	Silent	186,039	194,657	314,747

a SNP count; the overlapped SNP loci between samples were counted as one.

b Ts/Tv ratio: transition–transversion ratio (Ts/Tv) is a method to check the quality of the number of SNP calls.

c Because the analysis to categorize the SNPs was done non-exclusively, some SNPs were counted at multiple categories.

d SNP categories were clustered by six genomic regions: exon, splice site, intron, UTR, flanking region, and intergenic

e Upstream/downstream: 5 Kbp regions that are adjacent to the both ends of a gene were defined as upstream and downstream regions respectively. The regions (***) were not calculated as it is not required.

### Biological Process and Pathways of Yanbian Cattle Population

**Figure 1:**
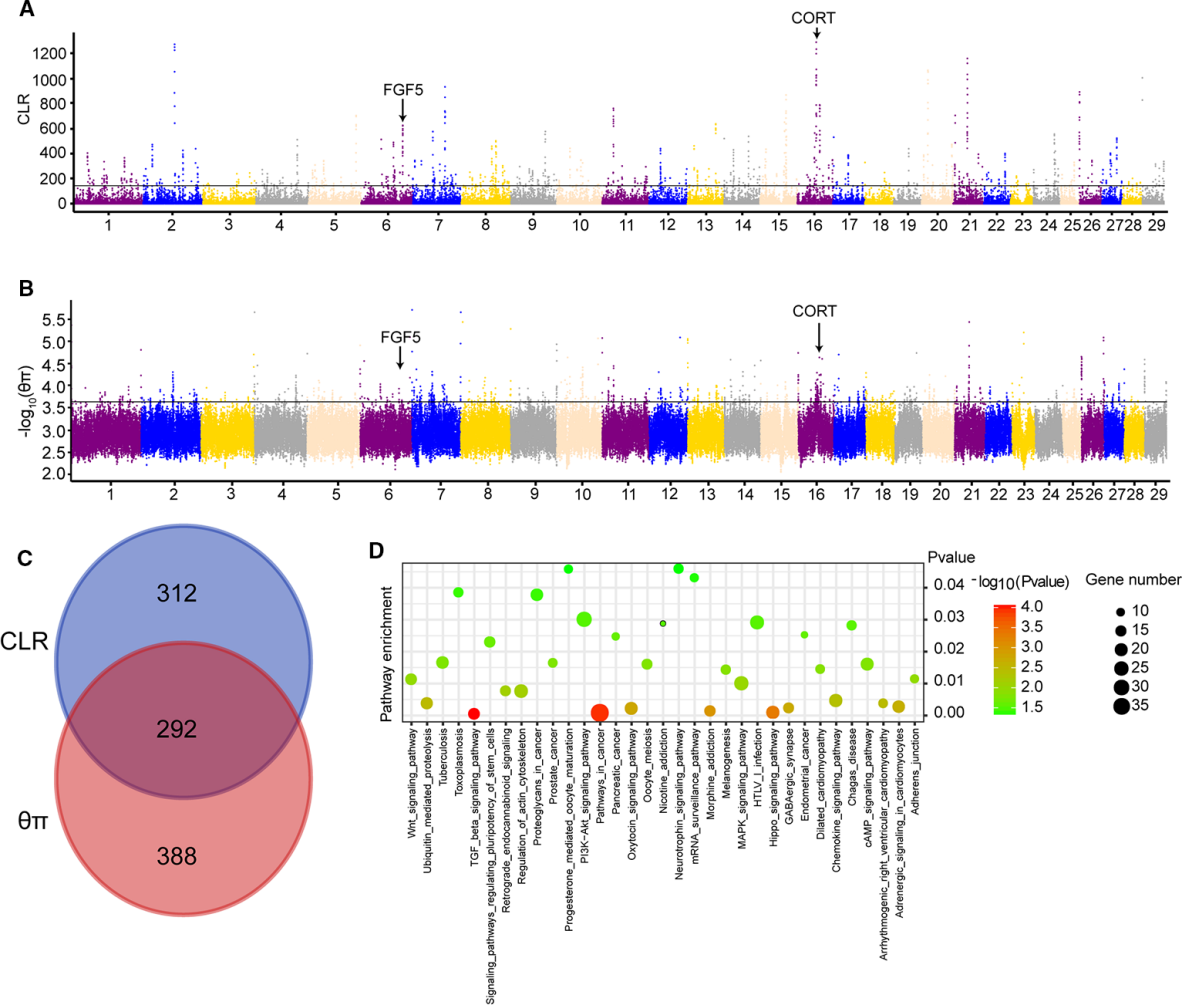
Genome-wide selection scan in Yanbian cattle. **(A, B)** Manhattan plot of the genome-wide distribution of composite likelihood ratio (CLR) and qp in Yanbian cattle using 50 kb windows size and 20 kb step size, respectively. **(C)** Venn diagram showing the genes overlap among CLR and qp significant selection region. **(D)** Kyoto Encyclopedia of Genes and Genomes (KEGG) pathway analysis of differentially expressed genes. Advanced bubble chart shows enrichment of differentially expressed genes in signaling pathways. Y-axis label represents pathway, and X-axis label represents rich factor (rich factor = amount of differentially expressed genes enriched in the pathway/amount of all genes in background gene set). Size and color of the bubble represent amount of differentially expressed genes enriched in pathway and enrichment significance, respectively.

CLR and *θ*π statistics were calculated for the Yanbian cattle alone, to distinguish the positive selection region. Based on CLR test statistic, we obtained 604 putative genes (**Table S5**) whereas, 680 positively selected genes were detected by *θ*π test (**Table S6**). Of these, 292 overlapped genes were detected in both statistics (**Figure 1C**). GO and KEGG pathways were employed by KOBAS, whereas, only 34 enriched pathways were retained (correct *p* < 0.05; **Table S7**; **Figure 1D**).

### Positive Selective Signature in Yanbian Cattle Related to Cold Climate Adaptation

Among 292 candidate genes detected in both CLR and *θ*π, some positively selected genes were considered to be associated with cold climate adaptation (*CORT* and *FGF5*). The strongest selection signal found on BTA16 (16:43127555-43129154) contains the *CORT* gene (**Figures 1A, B**), which was reported to be related with the cold stress in mouse, chicken, and humans (Dronjak et al., 2004; Hangalapura et al., 2004). In the cold exposure experiment of rats, *CORT* in the blood of rats significantly increased after 2 h of cold exposure (Dronjak et al., 2004). Meanwhile, the regulatory mechanism of cold stress and stress was studied in chickens, and it was found that *CORT* in plasma was considerably different under variable cold stress levels (Hangalapura et al., 2004). To identify the potential causal mutation around *CORT* locus, we checked all of mutations of Yanbian cattle, and four missense mutations in *CORT* (c.269C > T, p.Lys90Ile; c.251A > G, p.Glu84Gly; c.112C > T, p.Pro38Ser; c.86G > A, p.Pro29His) were found.

Interestingly, we found a gene, fibroblast growth factor 5 (*FGF5*) on BTA6 from CLR test (**Figure 1A**), which has been reported to be related to the development of hair follicles and hair length in cat, dog and human (Drögemüller et al., 2007; Dierks et al., 2013; Higgins et al., 2014). Meanwhile, we also found that *FGF5* was located in a significant region on chromosome from *θ*π test (**Figure 1B**). Under cold stress conditions, long and dense hairs are very important in order to keep the body warm against the cold environment, a distinctive feature of Yanbian cattle.

### Biological Process and Pathway Between Yanbian and N’Dama Cattle

**Figure 2:**
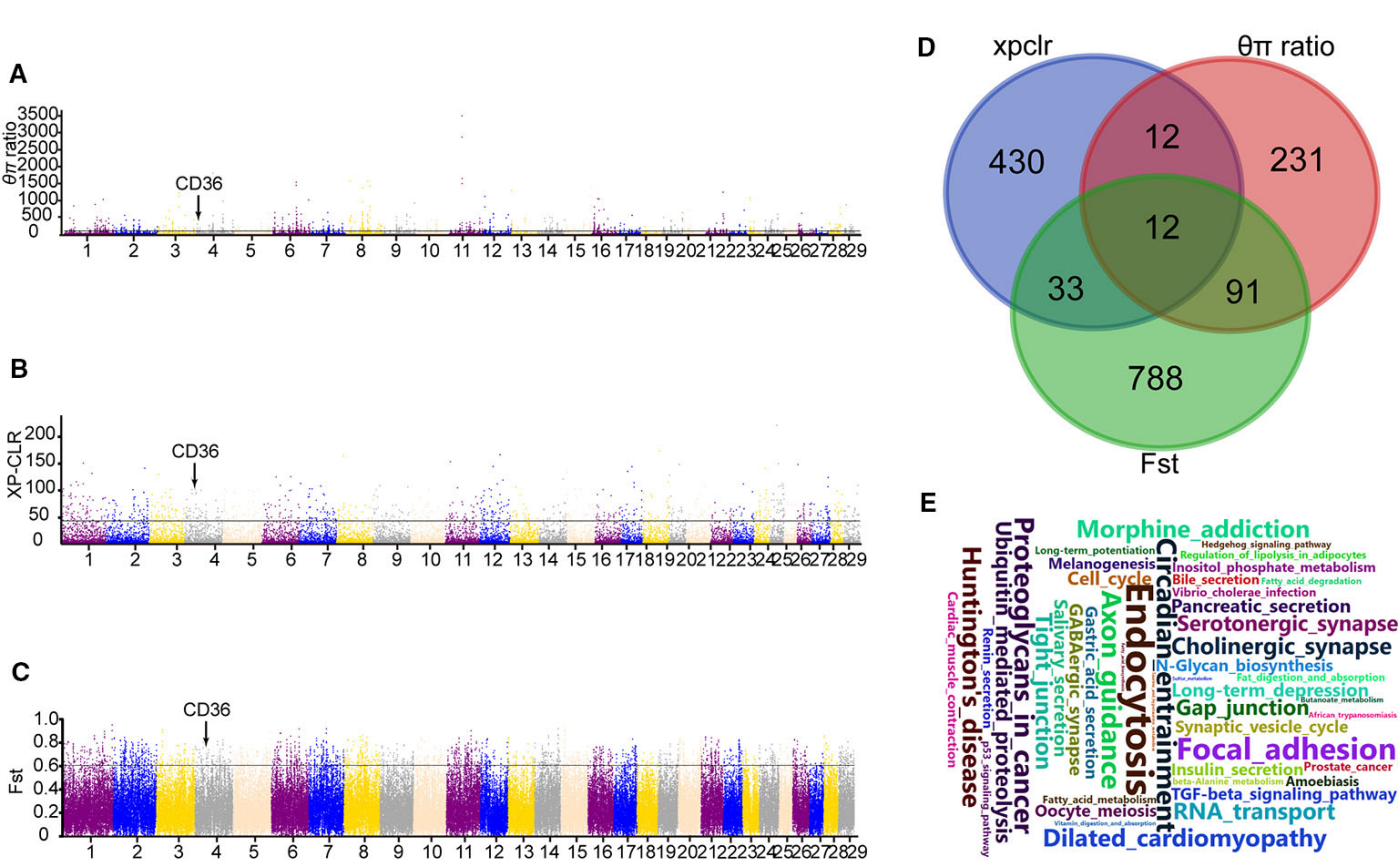
Genome-wide selection scan between Yanbian and N’Dama cattle. **(A–C)** Manhattan plot of the genome-wide distribution of qp ratio, XP-CLR, and Fst between Yanbian and N’Dama cattle using 50 kb windows size and 20 kb step size, respectively. The threshold corresponding to the top 0.5% of qp ratio, XP-CLR, and Fst are marked with a horizontal line, respectively. **(D)** Venn diagram showing the genes overlap among qp ratio, XP-CLR, and Fst significant selection region. **(E)** Word cloud illustrating major enrichment pathway of these genes in qp ratio, XP-CLR, and Fst significant selection region.

Moreover, XP-CLR test was performed to distinguish the positive selection region between Yanbian and N’Dama cattle. Based on the analysis, we obtained 487 putative genes (**Table S1**). Whereas, 924 and 346 candidate genes were identified using Fst and *θπ* ratio between the two populations, respectively. (**Tables S2** and **S3**). In addition, 12 overlapping genes from the three tests were detected and evaluated for the functional studies (**Figure 2D**). GO and KEGG pathways with KOBAS were used to build on biological modules consisting of clusters of functional terms. After wiping out duplicates, 1,375 genes were retained from XP-CLR, Fst, and *θ*π ratio for the analysis. KEGG pathway analysis resulted in 98 significantly enriched pathways (correct *p* < 0.05; **Table S4**). Vascular smooth muscle contraction, circadian entrainment, hypertrophic cardiomyopathy (HCM), fatty acid metabolism, and fat digestion and absorption were involved as major enrichment pathway, which may play an important role in the cold adaptation of Yanbian cattle.

### Candidate Regions and Genes Under Positive Selection in Yanbian and N’Dama Cattle

Here, we compared the genomes of Yanbian and N’Dama cattle to identify signatures of positive selection within each subspecies following environmental and artificial selection pressures. Twelve genes were overlapped in XPCLR, Fst, and *θ*π ratio methods including fatty acid synthase (*CD36*; **Figures 2A–C**), which plays main role in membrane transport in the heart and adipose tissue. The expression of *CD36* is increased in cold exposure, which enhances brown adipose tissue (BAT) uptake of TG-rich lipoprotein (*TRL*) and of albumin bound FA. Previous studies have shown that *CD36* gene in Hanwoo and Yanbian cattle affects the intramuscular fat deposition. Compared with N’Dama, Yanbian cattle have excellent meat quality. Yanbian cattle have been in the cold environment for a long time, and have a lot of fat deposits in their bodies, which is helpful to resist the cold. The divergent mutations of *CD36* gene were checked between Yanbian and N’Dama cattle, and one missense mutations (c.638A > G, p.Lys 213Arg) was found.

## Discussion

We herein carefully examined the whole genome resequencing of Yanbian and N’Dama cattle. The Ts/Tv ratio indicate the quality of resequencing, which was calculated to be 2.36 and 2.39 in Yanbian and N’Dama cattle respectively, comparable with the previous studies (**Tables 1** and **2**) (Choi et al., 2013; Choi et al., 2015). As for the heterozygous and homozygous ratio were concerned in the detected SNPs, the higher ratio of Yanbian cattle suggests that it’s population structure maybe normal and have a high heterozygosity rate.

Our study used resequencing data from Yanbian and N’Dama cattle to reveal a detailed genomic information along with candidate genes associated to the cold climate adaptation in Yanbian cattle. Yanbian cattle live in cold north China for a long time. Many biological traits in Yanbian cattle have adapted to the local cold environment, such as long and dense body hairs and increased muscular fat. In order to study the cold-adaptive mechanism of Yanbian cattle, N’Dama cattle was selected, a *Bos taurus* cattle living in the hot areas of Africa, as the reference. Cold adaptation research indicate that more than one mechanism involved in the biological response to cold stress. And it is a multi-factor complex trait that be affected by different factors at different levels in molecular and mechanical aspects. Consistent with the expectations of biological complexity of cold adaptation, our selection test highlights several different processes that are coherently responsible.

We calculated the CLR and *θ*π to analyze the positive selection of Yanbian cattle. The strongest selection signal of CLR found on BTA16 (16: 43127555-43129154) contains the *CORT* gene (**Figure 1A**), which was reported to be related with the cold stress in mouse, chicken and humans (Dronjak et al., 2004; Hangalapura et al., 2004). Secretion of *CORT*, a primary hormone in the hypothalamic-pituitary-adrenal (*HPA*) axis, exhibits a circadian rhythm in many species (Mormède et al., 2007). At the same time, *CORT* levels are also used as one of the biochemical parameters used to measure the physiological response of animals to stressful environments. Hence, increased cortisol levels in blood are an important stress indicator (Grandin, 1997; Ndlovu et al., 2008). In the cold exposure experiment of rats, *CORT* in the blood of rats significantly increased after 2 h of cold exposure (Dronjak et al., 2004). Meanwhile, the regulatory mechanism of cold stress and stress was studied in chickens, and it was found that *CORT* in plasma was considerably different under variable cold stress levels (Hangalapura et al., 2004). At the same time, we found four missense mutations in *CORT* (c.269C > T, p.Lys90Ile; c.251A > G, p.Glu84Gly; c.112C > T, p.Pro38Ser; c.86G > A, p.Pro29His). Also, *CORT* is one of the biochemical parameters used to measure the physiological response of animals to stressful environments. Under different cold stress levels, the study on the regulation mechanism of cold stress and stress in chickens found that *CORT* levels in plasma were significantly different (Hangalapura et al., 2004). Meanwhile, we also identified another gene *FGF5* that may influence the hair length and density in Yanbian cattle, which is very important for keeping the body warm against the cold environment. *FGF5* has been reported to be related to the development of hair follicles and hair length in cat, dog and human (Drögemüller et al., 2007; Dierks et al., 2013; Higgins et al., 2014). Though, analyzing the selection region of CLR test, *FGF5* was found to be on top prominent point on BTA6 (**Figure 1A**), and also was located in a significant region on BTA6 from *θ*π test (**Figure 1B**).

Three methods (XP-CLR, Fst and *θ*π ratio) were used to analysis the whole genome data and choose the significant signals between Yanbian and N’Dama cattle. Twelve genes (*RBFOX1*, *CD36*, *GRXCR2*, *KCNB2*, *NSG2*, *ROBO1*, *NRXN1*, *LINGO2*, *GRM5*, and *AMOTL1*) were overlapped among XP-CLR, Fst, and *θ*π ratio. Among all, *CD36* plays an important role in membrane transport of long-chain fatty acid (FA) in the heart, skeletal muscle, and adipose tissue (Glatz et al., 2010). The expression of *CD36* is increased in cold exposure, which enhances BAT uptake of TG-rich lipoprotein (TRL) and of albumin bound FA (Bartelt et al., 2011). Previous studies have shown that *CD36* gene in Hanwoo and Yanbian cattle affects the intramuscular fat deposition (Jeong et al., 2012). Also, the expression of *CD36* has been proven to be positively correlated with obesity in dairy cows (Prodanović et al., 2016). Compared with N’Dama, Yanbian cattle have excellent meat quality. Yanbian cattle have been in the cold environment for a long time, and have a lot of fat deposits in their bodies, which is helpful to resist the cold. There are some pathways [fat digestion and absorption, AMPK signaling pathway, phagosome, extracellular matrix receptors (ECM)-receptor interaction] represented in Yanbian and N’Dama cattle include *CD36* genes (**Figures 2A–C**). Studies have shown that fat digestion and absorption pathway affects the heat production of animals, *CD36* gene plays an indispensable role in the heat production (Putri et al., 2015). Also, the expression of *CD36* has been reported to be positively correlated with obesity in dairy cows (Prodanović et al., 2016).

As far as we know, Yanbian cattle as beef cattle is rich in muscle fat. The phenomenon of fat abundance in muscle of Yanbian cattle may be one of the mechanisms for resisting cold climate. A missense mutation (c.638A > G, p.Lys 213Arg) was been found between Yanbian and N’Dama cattle, we speculate that this may be one of the causes of Yanbian cattle cold tolerance.

## Data Availability Statement

The bioproject number of the sequencing data information about Yanbian cattle is PRJNA565271 in the NCBI Sequence Read Archive. The sequenced N’Dama cattle genomes in this study are publicly available from GenBank with the Bioproject accession number PRJNA312138.

## Ethics Statement

The animal study was reviewed and approved by Institutional Animal Care and Use Committee of Northwest A&F University following the recommendation of the Regulations for the Administration of Affairs Concerning Experimental Animals of China.

## Author Contributions

JS and QH contributed equally towards the construction and execution of this manuscript. YC and YY helped in sample collection, CL revised the manuscript and provided with the valuable suggestion. GZ and YZ contributed in the funding for the research.

## Funding

This work was supported by Natural Science Foundation of China (No. 31872317), the Program of National Beef Cattle and Yak Industrial Technology System (No. CARS-37).

## Conflict of Interest

The authors declare that the research was conducted in the absence of any commercial or financial relationships that could be construed as a potential conflict of interest.
