# Supplementary material for: Whole Genome Scan and Selection Signatures for Climate Adaption in Yanbian Cattle
Source: Front Genet. 2020 Feb 25;11:94. doi: 10.3389/fgene.2020.00094 (PMC7059643; doi:10.3389/fgene.2020.00094)
Supplement: Supplementary file 1 [file DataSheet_1.docx]

**Whole genome scan and selection signatures for climate adaption in Yanbian cattle**

**Jiafei Shen^1,2,a^, Quratulain Hanif^3,a^, Yang Cao^1^, Yongsheng Yu^1^, Chuzhao Lei^2^, Guoliang Zhang^1^, and Yumin Zhao^1^****^,^**^b^

^1^ Key Laboratory of Beef Cattle Genetics and Breeding in Ministry of Agriculture and Rural Agriculture, Branch of Animal Husbandry, Jilin Academy of Agricultural Sciences, Changchun 130033, China.

^2^ College of Animal Science and Technology, Northwest A&F University, Yangling, Shaanxi 712100, China.

^3^ National Institute for Biotechnology and Genetic Engineering, Pakistan Institute of Engineering and Applied Sciences, Faisalabad, 577, Pakistan

^a^ *These authors contributed equally to this work.*

^b^ Corresponding author: Yumin Zhao, [yuminzhao@126.com](mailto:yuminzhao@126.com)

**Supplementary Information**

|  | **Supplementary Notes** | **Page** |  |
| --- | --- | --- | --- |
|  | Supplementary note 1 | 1-3 |  |
|  |  |  |  |
|  | **Supplementary Figures** | **Page** |  |
|  | Supplementary figure 1 | 4 |  |
|  |  |  |  |
|  | **Supplementary Tables** | **Page** |  |
|  | Supplementary Table S1 | 5-17 |  |
|  | Supplementary Table S2 | 18-39 |  |
|  | Supplementary Table S3 | 40-50 |  |
|  | Supplementary Table S4 | 51-61 |  |
|  | Supplementary Table S5 | 62-73 |  |
|  | Supplementary Table S6 | 74-86 |  |
|  | Supplementary Table S7 | 87-90 |  |

**Supplementary Note 1**

**Whole-genome resequencing**

In total, 9 DNA samples of Yanbian cattle were collected. Paired-end libraries were generated for each individual using standard procedures. The average insert size was 500 bp, and read length was 150 bp. All libraries were sequenced on an Illumina HiSeq 2000 platform to an average raw read sequence coverage of 10.8X, assuming a genome size of 2.72 Gb. The average coverage was approximately 9.15 X (ranging from 7.5 X to 10.8 X). Such depth ensured the accuracy of variant calling and genotyping, and met the requirement for population genetic analyses.

**Variant discovery and genotyping**

A total of 19 samples were used as final set for variant discovery. First, all cleaned reads were mapped to the cattle reference assembly ARS-UCD1.2 using BWA-MEM (0.7.13-r1126) with default parameters (Li and Durbin, 2009). The average mapping rate was 99.16%, and the sequencing coverage was approximately 9.15 X. Duplicate reads were removed using Picard Tools (<http://broadinstitute.github.io/picard/>). Then, the Genome Analysis Toolkit (GATK, version 3.6-0-g89b7209) was used to detect single nucleotide polymorphisms (SNPs) (Danecek *et al.*, 2011). The following criteria were applied to all SNPs: (1) SNPs mean sequencing depth (over all included individuals) < 1/3X and > 3X were filtered; (2) SNPs with Variant Confidence/Quality by Depth (QD) < 2 were filtered; (3) SNPs with RMS Mapping Quality (MQ) < 40.0 were filtered; (4) SNPs with Phred-scaled P-value using Fisher’s exact test to detect strand bias (FS) > 60 were filtered; (5) SNPs with Z-score according to the Wilcoxon rank sum test of Alt vs. Ref read mapping qualities (MQRankSum) < -12.5 were filtered; (6) SNPs with Z-score according to the Wilcoxon rank sum test of Alt vs. Ref read position bias (ReadPosRankSum) < -8 were filtered; (7) SNPs with maximum missing rate < 0.1; and (8) SNPs with only two alleles. A total of 12.3 million autosomal SNPs were identified and used in subsequent analyses. The identified SNPs were further classified based on the gene annotation for the reference genome. SNPs were categorized as variants in intergenic regions, 5′-UTRs, coding sequences (exon), introns, 3′-UTRs and noncoding regions. SNPs in coding sequences were further grouped as synonymous SNPs not causing amino acid changes or nonsynonymous SNPs causing amino acid changes. In addition, nonsense mutations were defined as mutations causing premature stops, elongated transcripts or introduced false start codons. The distribution of SNPs within various genomic regions is shown in Table 2.

**Supplementary Figures**


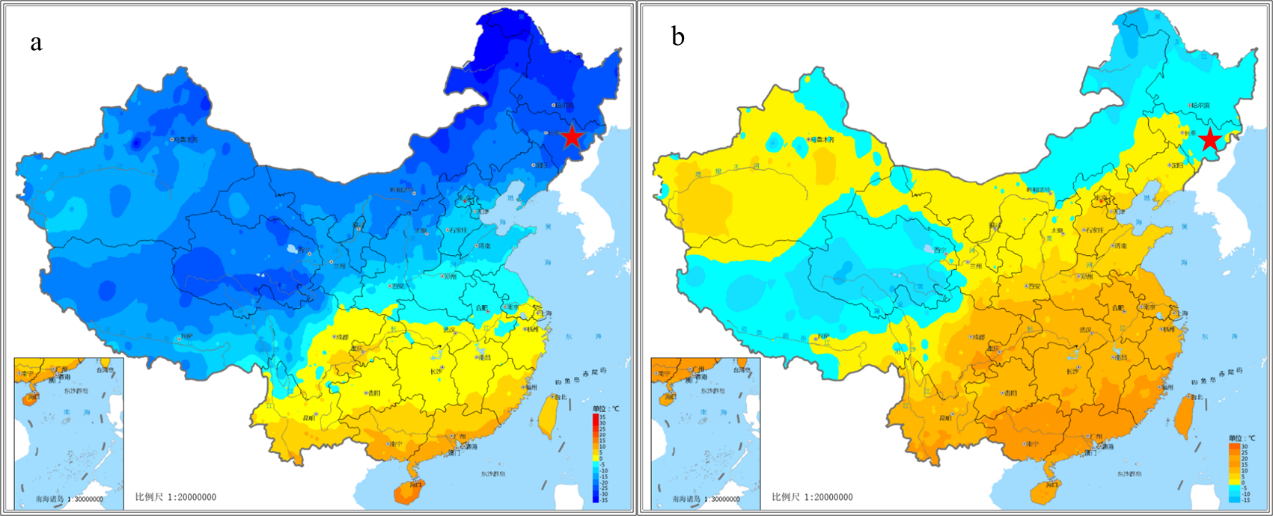


Supplementary Figure 1. Diagram of surface temperature in China. The figure shows the distribution of average temperature in China during the years. The different colors on the scale represent different temperatures, blue for low temperatures, yellow for high temperatures, and asterisks for living areas of Yanbian cattle. Figure a shows the China’s average temperature distribution in January, and the temperature in the Yanbian cattle living area is below -30 degrees Celsius. Figure b is the average annual temperature in China, and the living area of Yanbian cattle is also below 0 degrees Celsius.

**Table S1**. A summary of genes from XP-CLR (*P*-value < 0.5%) between Yanbian and N'Dama cattle

| CHROM | START | END | XPCLR | GENE |
| --- | --- | --- | --- | --- |
| 19 | 19325961 | 19375961 | 149.81823 | LGALS9 |
| 12 | 24914833 | 24964833 | 146.14546 | SERTM1 |
| 2 | 72925278 | 72975278 | 142.785371 | TFCP2L1 |
| 17 | 12018957 | 12068957 | 137.114316 | SLC10A7 |
| 1 | 56579116 | 56629116 | 133.02089 | PHLDB2 |
| 3 | 17803417 | 17853417 | 131.717234 | IVL |
| 6 | 26850345 | 26900345 | 126.428017 | STPG2 |
| 4 | 12532185 | 12582185 | 124.422786 | PON1 |
| 4 | 12532185 | 12582185 | 124.422786 | PPP1R9A |
| 14 | 45062803 | 45112803 | 121.932657 | COLEC10 |
| 1 | 27029116 | 27079116 | 117.081285 | ROBO1 |
| 18 | 47054930 | 47104930 | 116.167793 | ZNF566 |
| 17 | 24068957 | 24118957 | 115.412894 | PABPC4L |
| 24 | 23800927 | 23850927 | 113.209077 | CCDC178 |
| 22 | 19008988 | 19058988 | 112.93789 | GRM7 |
| 29 | 26576202 | 26626202 | 110.233052 | OR10S1 |
| 29 | 26576202 | 26626202 | 110.233052 | OR4D5 |
| 6 | 34450345 | 34500345 | 110.204455 | CCSER1 |
| 24 | 21200927 | 21250927 | 109.551093 | MIR187 |
| 9 | 12757723 | 12807723 | 109.315887 | DDX43 |
| 9 | 12757723 | 12807723 | 109.315887 | OOEP |
| 24 | 24800927 | 24850927 | 109.112498 | GAREM1 |
| 20 | 36910001 | 36960001 | 108.607362 | WDR70 |
| 11 | 22451746 | 22501746 | 105.612858 | SLC8A1 |
| 5 | 4422009 | 4472009 | 104.353282 | TRNAY-GUA |
| 7 | 19815379 | 19865379 | 103.770609 | SIRT6 |
| 7 | 19815379 | 19865379 | 103.770609 | ANKRD24 |
| 7 | 19815379 | 19865379 | 103.770609 | EBI3 |
| 1 | 3129116 | 3179116 | 103.525973 | URB1 |
| 15 | 25080381 | 25130381 | 103.026631 | NXPE4 |
| 10 | 34110336 | 34160336 | 102.465808 | RASGRP1 |
| 3 | 40303417 | 40353417 | 102.430975 | COL11A1 |
| 4 | 13482185 | 13532185 | 102.363595 | SLC25A13 |
| 5 | 66622009 | 66672009 | 101.902114 | PAH |
| 12 | 21364833 | 21414833 | 101.178273 | ATP7B |
| 5 | 48672009 | 48722009 | 100.916465 | WIF1 |
| 28 | 12600777 | 12650777 | 99.684149 | CHRM3 |
| 2 | 70975278 | 71025278 | 97.287243 | STEAP3 |
| 7 | 45615379 | 45665379 | 93.820588 | VDAC1 |
| 7 | 45615379 | 45665379 | 93.820588 | C7H5orf15 |
| 7 | 18915379 | 18965379 | 93.757986 | PTPRS |
| 24 | 23950927 | 24000927 | 92.926207 | CCDC178 |
| 18 | 14904930 | 14954930 | 92.852753 | SHCBP1 |
| 16 | 4350477 | 4400477 | 92.613376 | EIF2D |
| 16 | 4350477 | 4400477 | 92.613376 | RASSF5 |
| 5 | 2022009 | 2072009 | 92.007073 | TRHDE |
| 13 | 18750789 | 18800789 | 91.713439 | PARD3 |
| 16 | 30100477 | 30150477 | 90.685247 | CDC42BPA |
| 7 | 18815379 | 18865379 | 88.698717 | ZNRF4 |
| 15 | 16830381 | 16880381 | 88.618079 | GUCY1A2 |
| 12 | 21264833 | 21314833 | 88.545272 | CCDC70 |
| 9 | 8657723 | 8707723 | 88.511718 | LMBRD1 |
| 1 | 25929116 | 25979116 | 88.484751 | TRNAG-UCC |
| 1 | 52679116 | 52729116 | 88.449589 | CD47 |
| 10 | 17510336 | 17560336 | 88.296749 | UACA |
| 2 | 52675278 | 52725278 | 87.805031 | GTDC1 |
| 3 | 23553417 | 23603417 | 87.676907 | PHGDH |
| 3 | 23553417 | 23603417 | 87.676907 | HMGCS2 |
| 3 | 40353417 | 40403417 | 87.417724 | COL11A1 |
| 1 | 81629116 | 81679116 | 86.713598 | LIPH |
| 12 | 2564833 | 2614833 | 86.229789 | DIAPH3 |
| 1 | 78529116 | 78579116 | 85.348004 | LPP |
| 23 | 20650117 | 20700117 | 85.227693 | CD2AP |
| 15 | 1280381 | 1330381 | 84.590778 | IZUMO1R |
| 2 | 52425278 | 52475278 | 84.074894 | GTDC1 |
| 20 | 20460001 | 20510001 | 83.410416 | RAB3C |
| 6 | 33050345 | 33100345 | 82.484787 | CCSER1 |
| 1 | 58479116 | 58529116 | 82.073205 | GRAMD1C |
| 1 | 58479116 | 58529116 | 82.073205 | ATP6V1A |
| 24 | 20350927 | 20400927 | 82.069434 | FHOD3 |
| 24 | 7200927 | 7250927 | 81.766634 | RTTN |
| 2 | 15625278 | 15675278 | 81.301853 | UBE2E3 |
| 24 | 20600927 | 20650927 | 81.2501 | FHOD3 |
| 23 | 9850117 | 9900117 | 79.848449 | SLC26A8 |
| 23 | 9850117 | 9900117 | 79.848449 | SRPK1 |
| 11 | 30851746 | 30901746 | 79.054888 | STON1 |
| 25 | 5554306 | 5604306 | 78.330858 | RBFOX1 |
| 12 | 40214833 | 40264833 | 78.282521 | PCDH9 |
| 28 | 15700777 | 15750777 | 77.719543 | ANK3 |
| 3 | 16053417 | 16103417 | 77.716098 | SHE |
| 3 | 16053417 | 16103417 | 77.716098 | TDRD10 |
| 10 | 32510336 | 32560336 | 77.690436 | MEIS2 |
| 12 | 33314833 | 33364833 | 77.324701 | SHISA2 |
| 24 | 20150927 | 20200927 | 77.10755 | KIAA1328 |
| 14 | 47112803 | 47162803 | 76.799122 | SLC30A8 |
| 2 | 6175278 | 6225278 | 76.597429 | C2H2orf88 |
| 2 | 18375278 | 18425278 | 75.862736 | FKBP7 |
| 2 | 18375278 | 18425278 | 75.862736 | PLEKHA3 |
| 5 | 48972009 | 49022009 | 75.795616 | TBC1D30 |
| 7 | 50315379 | 50365379 | 75.751912 | SIL1 |
| 13 | 25150789 | 25200789 | 75.064401 | KIAA1217 |
| 24 | 21700927 | 21750927 | 74.778308 | ZNF397 |
| 24 | 21700927 | 21750927 | 74.778308 | ZNF24 |
| 25 | 7604306 | 7654306 | 74.554743 | PMM2 |
| 25 | 7604306 | 7654306 | 74.554743 | TMEM186 |
| 25 | 7604306 | 7654306 | 74.554743 | ABAT |
| 2 | 52325278 | 52375278 | 74.50989 | GTDC1 |
| 26 | 26062988 | 26112988 | 74.488435 | SORCS3 |
| 26 | 23512988 | 23562988 | 74.422212 | CNNM2 |
| 26 | 23512988 | 23562988 | 74.422212 | AS3MT |
| 27 | 4003840 | 4053840 | 74.276749 | CSMD1 |
| 17 | 10668957 | 10718957 | 74.251932 | EDNRA |
| 28 | 9350777 | 9400777 | 74.172736 | ACTN2 |
| 26 | 13212988 | 13262988 | 74.020102 | PPP1R3C |
| 15 | 47980381 | 48030381 | 73.996996 | MGC137098 |
| 15 | 47980381 | 48030381 | 73.996996 | OR52H1 |
| 2 | 52475278 | 52525278 | 73.908936 | GTDC1 |
| 10 | 29510336 | 29560336 | 73.758144 | FMN1 |
| 6 | 68500345 | 68550345 | 73.753895 | SCFD2 |
| 6 | 68500345 | 68550345 | 73.753895 | RASL11B |
| 25 | 13404306 | 13454306 | 73.488201 | PARN |
| 8 | 53822087 | 53872087 | 72.8328 | GNAQ |
| 6 | 17550345 | 17600345 | 72.798106 | PAPSS1 |
| 18 | 46804930 | 46854930 | 72.779705 | ZNF565 |
| 18 | 46804930 | 46854930 | 72.779705 | ZNF146 |
| 18 | 46804930 | 46854930 | 72.779705 | COX7A1 |
| 18 | 46804930 | 46854930 | 72.779705 | CAPNS1 |
| 14 | 36262803 | 36312803 | 72.774357 | KCNB2 |
| 11 | 47951746 | 48001746 | 72.382959 | KRCC1 |
| 11 | 47951746 | 48001746 | 72.382959 | SMYD1 |
| 12 | 50464833 | 50514833 | 72.020867 | TBC1D4 |
| 16 | 3950477 | 4000477 | 72.015541 | SRGAP2 |
| 16 | 3950477 | 4000477 | 72.015541 | FAM72A |
| 8 | 26672087 | 26722087 | 71.817582 | SH3GL2 |
| 25 | 21604306 | 21654306 | 71.701465 | PRKCB |
| 15 | 41380381 | 41430381 | 71.653797 | GALNT18 |
| 23 | 9750117 | 9800117 | 71.052696 | SRPK1 |
| 23 | 9750117 | 9800117 | 71.052696 | LHFPL5 |
| 23 | 9750117 | 9800117 | 71.052696 | CLPS |
| 3 | 23953417 | 24003417 | 70.973831 | WARS2 |
| 20 | 17910001 | 17960001 | 70.679941 | ZSWIM6 |
| 3 | 10803417 | 10853417 | 70.538706 | AIM2 |
| 6 | 25400345 | 25450345 | 70.436514 | ADH6 |
| 1 | 47529116 | 47579116 | 70.144307 | TRNAF-AAA |
| 29 | 10576202 | 10626202 | 69.653978 | DLG2 |
| 22 | 12158988 | 12208988 | 69.302126 | SCN10A |
| 3 | 29253417 | 29303417 | 69.190013 | SYT6 |
| 19 | 675961 | 725961 | 69.129284 | CA10 |
| 7 | 21165379 | 21215379 | 69.001614 | TMPRSS9 |
| 28 | 12500777 | 12550777 | 68.64303 | CHRM3 |
| 18 | 47154930 | 47204930 | 68.490015 | ZNF568 |
| 19 | 22975961 | 23025961 | 68.345 | RTN4RL1 |
| 8 | 62072087 | 62122087 | 68.313081 | MIR2473 |
| 8 | 62072087 | 62122087 | 68.313081 | MIR2472 |
| 8 | 62072087 | 62122087 | 68.313081 | SHB |
| 16 | 6450477 | 6500477 | 68.215848 | KCNT2 |
| 12 | 40164833 | 40214833 | 68.16858 | PCDH9 |
| 10 | 50760336 | 50810336 | 68.097006 | FAM81A |
| 9 | 9957723 | 10007723 | 67.872371 | B3GAT2 |
| 9 | 9957723 | 10007723 | 67.872371 | SMAP1 |
| 20 | 41160001 | 41210001 | 67.277932 | TRNAG-CCC |
| 16 | 45850477 | 45900477 | 66.752546 | CAMTA1 |
| 4 | 62732185 | 62782185 | 66.496833 | BMPER |
| 24 | 25450927 | 25500927 | 66.474178 | B4GALT6 |
| 18 | 47504930 | 47554930 | 66.374707 | ZNF420 |
| 4 | 20632185 | 20682185 | 66.250681 | SCIN |
| 4 | 9682185 | 9732185 | 66.167587 | ANKIB1 |
| 5 | 70922009 | 70972009 | 65.603423 | PWP1 |
| 13 | 29650789 | 29700789 | 65.559474 | NMT2 |
| 13 | 29650789 | 29700789 | 65.559474 | RPP38 |
| 13 | 29650789 | 29700789 | 65.559474 | ACBD7 |
| 13 | 29650789 | 29700789 | 65.559474 | OLAH |
| 14 | 8862803 | 8912803 | 65.271016 | KCNQ3 |
| 3 | 42753417 | 42803417 | 65.269915 | CDC14A |
| 2 | 52725278 | 52775278 | 65.115691 | GTDC1 |
| 26 | 24612988 | 24662988 | 65.083778 | COL17A1 |
| 3 | 49253417 | 49303417 | 64.867199 | ARHGAP29 |
| 10 | 3260336 | 3310336 | 64.844097 | KCNN2 |
| 1 | 73879116 | 73929116 | 64.831634 | ATP13A4 |
| 7 | 45015379 | 45065379 | 64.830544 | FSTL4 |
| 24 | 24000927 | 24050927 | 64.828986 | CCDC178 |
| 2 | 70725278 | 70775278 | 64.372949 | MARCO |
| 3 | 29753417 | 29803417 | 64.358306 | MAGI3 |
| 8 | 4422087 | 4472087 | 63.91608 | GALNTL6 |
| 10 | 6960336 | 7010336 | 63.84662 | ANKDD1B |
| 10 | 6960336 | 7010336 | 63.84662 | POLK |
| 6 | 44100345 | 44150345 | 63.779813 | DHX15 |
| 28 | 9300777 | 9350777 | 63.584498 | ACTN2 |
| 20 | 28660001 | 28710001 | 63.540743 | EMB |
| 24 | 13850927 | 13900927 | 63.535309 | PIK3C3 |
| 23 | 20800117 | 20850117 | 63.487259 | OPN5 |
| 19 | 23675961 | 23725961 | 63.487041 | RAP1GAP2 |
| 9 | 49107723 | 49157723 | 63.485965 | ASCC3 |
| 2 | 725278 | 775278 | 63.320486 | HERC2 |
| 2 | 63925278 | 63975278 | 63.312904 | NCKAP5 |
| 23 | 9900117 | 9950117 | 63.301389 | SLC26A8 |
| 21 | 24262447 | 24312447 | 62.911986 | ADAMTSL3 |
| 24 | 20100927 | 20150927 | 62.662685 | KIAA1328 |
| 19 | 24025961 | 24075961 | 62.426456 | OR3A1 |
| 1 | 66329116 | 66379116 | 62.305486 | SLC15A2 |
| 1 | 66329116 | 66379116 | 62.305486 | EAF2 |
| 24 | 23550927 | 23600927 | 62.071181 | ASXL3 |
| 13 | 9450789 | 9500789 | 62.05348 | MACROD2 |
| 23 | 27200117 | 27250117 | 61.985491 | PRRT1 |
| 23 | 27200117 | 27250117 | 61.985491 | PPT2 |
| 23 | 27200117 | 27250117 | 61.985491 | EGFL8 |
| 23 | 27200117 | 27250117 | 61.985491 | AGPAT1 |
| 23 | 27200117 | 27250117 | 61.985491 | RNF5 |
| 23 | 27200117 | 27250117 | 61.985491 | AGER |
| 23 | 27200117 | 27250117 | 61.985491 | PBX2 |
| 17 | 1968957 | 2018957 | 61.765548 | MAP9 |
| 12 | 23014833 | 23064833 | 61.756962 | LHFPL6 |
| 20 | 33910001 | 33960001 | 61.702602 | TRNAE-CUC |
| 3 | 49703417 | 49753417 | 61.679719 | BCAR3 |
| 24 | 20300927 | 20350927 | 61.637738 | FHOD3 |
| 24 | 20300927 | 20350927 | 61.637738 | TPGS2 |
| 28 | 8500777 | 8550777 | 61.599025 | LYST |
| 10 | 37310336 | 37360336 | 61.531451 | EHD4 |
| 27 | 33003840 | 33053840 | 61.486078 | PLPBP |
| 27 | 33003840 | 33053840 | 61.486078 | ERLIN2 |
| 18 | 2004930 | 2054930 | 61.327785 | GLG1 |
| 19 | 19125961 | 19175961 | 61.105514 | KSR1 |
| 15 | 15730381 | 15780381 | 61.089588 | AMOTL1 |
| 22 | 558988 | 608988 | 60.871958 | VOPP1 |
| 5 | 5872009 | 5922009 | 60.860063 | OSBPL8 |
| 4 | 40382185 | 40432185 | 60.853042 | CD36 |
| 23 | 11950117 | 12000117 | 60.713529 | ZFAND3 |
| 1 | 22929116 | 22979116 | 60.677427 | LIPI |
| 1 | 22929116 | 22979116 | 60.677427 | RBM11 |
| 3 | 18153417 | 18203417 | 60.534526 | CRCT1 |
| 8 | 22772087 | 22822087 | 60.175371 | IFN-TAU |
| 12 | 24714833 | 24764833 | 60.08291 | SMAD9 |
| 12 | 24714833 | 24764833 | 60.08291 | ALG5 |
| 3 | 37103417 | 37153417 | 59.918897 | TRNAC-GCA |
| 23 | 4450117 | 4500117 | 59.753071 | BMP5 |
| 8 | 48272087 | 48322087 | 59.624856 | C8H9orf57 |
| 7 | 40715379 | 40765379 | 59.601621 | OR2B11 |
| 2 | 17275278 | 17325278 | 59.415455 | ZNF385B |
| 25 | 2504306 | 2554306 | 58.997035 | ZSCAN10 |
| 19 | 8725961 | 8775961 | 58.921098 | VEZF1 |
| 19 | 8725961 | 8775961 | 58.921098 | CUEDC1 |
| 23 | 11100117 | 11150117 | 58.818356 | TBC1D22B |
| 23 | 11100117 | 11150117 | 58.818356 | TMEM217 |
| 12 | 14464833 | 14514833 | 58.7418 | SMIM2 |
| 20 | 28710001 | 28760001 | 58.708708 | EMB |
| 26 | 24462988 | 24512988 | 58.690317 | STN1 |
| 12 | 22914833 | 22964833 | 58.638379 | LHFPL6 |
| 9 | 67357723 | 67407723 | 58.489115 | LAMA2 |
| 18 | 11104930 | 11154930 | 58.486138 | KIAA0513 |
| 18 | 11104930 | 11154930 | 58.486138 | ZDHHC7 |
| 16 | 38950477 | 39000477 | 58.38937 | PRRC2C |
| 4 | 32232185 | 32282185 | 58.36966 | CCDC126 |
| 2 | 29025278 | 29075278 | 58.365684 | MIR2285W |
| 2 | 29025278 | 29075278 | 58.365684 | XIRP2 |
| 4 | 16682185 | 16732185 | 58.340872 | NXPH1 |
| 28 | 15950777 | 16000777 | 57.993869 | ANK3 |
| 16 | 45500477 | 45550477 | 57.807335 | PER3 |
| 16 | 45500477 | 45550477 | 57.807335 | UTS2 |
| 7 | 52565379 | 52615379 | 57.688119 | DIAPH1 |
| 7 | 52565379 | 52615379 | 57.688119 | PCDHGC3 |
| 7 | 52565379 | 52615379 | 57.688119 | PCDHGA8 |
| 7 | 52565379 | 52615379 | 57.688119 | PCDHGB4 |
| 7 | 52565379 | 52615379 | 57.688119 | PCDHGA2 |
| 11 | 20051746 | 20101746 | 57.582992 | CDC42EP3 |
| 18 | 48204930 | 48254930 | 57.206271 | RYR1 |
| 18 | 48204930 | 48254930 | 57.206271 | RASGRP4 |
| 18 | 48204930 | 48254930 | 57.206271 | FAM98C |
| 18 | 48204930 | 48254930 | 57.206271 | SPRED3 |
| 11 | 11901746 | 11951746 | 57.198331 | EXOC6B |
| 28 | 13750777 | 13800777 | 57.082688 | FXYD4 |
| 15 | 31730381 | 31780381 | 56.711204 | TECTA |
| 14 | 27162803 | 27212803 | 56.685986 | ASPH |
| 5 | 68372009 | 68422009 | 56.612697 | SLC41A2 |
| 5 | 8722009 | 8772009 | 56.478304 | SYT1 |
| 22 | 37808988 | 37858988 | 56.417517 | SYNPR |
| 15 | 1380381 | 1430381 | 56.27511 | GPR83 |
| 7 | 57065379 | 57115379 | 56.201331 | GRXCR2 |
| 16 | 4500477 | 4550477 | 56.160476 | MAPKAPK2 |
| 13 | 25250789 | 25300789 | 56.11304 | KIAA1217 |
| 11 | 7101746 | 7151746 | 55.941502 | IL1RL1 |
| 13 | 12000789 | 12050789 | 55.870273 | CAMK1D |
| 3 | 49353417 | 49403417 | 55.770767 | ABCA4 |
| 26 | 25812988 | 25862988 | 55.756733 | SORCS3 |
| 11 | 37301746 | 37351746 | 55.740092 | MIR2296 |
| 11 | 37301746 | 37351746 | 55.740092 | SPTBN1 |
| 3 | 15153417 | 15203417 | 55.549792 | ASH1L |
| 12 | 13714833 | 13764833 | 55.534733 | ENOX1 |
| 29 | 28976202 | 29026202 | 55.529547 | STT3A |
| 29 | 28976202 | 29026202 | 55.529547 | EI24 |
| 18 | 11854930 | 11904930 | 55.504811 | MIR2325A |
| 18 | 11854930 | 11904930 | 55.504811 | IRF8 |
| 12 | 24614833 | 24664833 | 55.500021 | SUPT20H |
| 6 | 33300345 | 33350345 | 55.397691 | CCSER1 |
| 13 | 12450789 | 12500789 | 55.264304 | ECHDC3 |
| 13 | 12450789 | 12500789 | 55.264304 | PROSER2 |
| 8 | 16272087 | 16322087 | 55.220327 | LINGO2 |
| 1 | 44379116 | 44429116 | 55.216528 | FILIP1L |
| 1 | 44379116 | 44429116 | 55.216528 | CMSS1 |
| 16 | 35050477 | 35100477 | 55.152554 | WDR64 |
| 20 | 4710001 | 4760001 | 55.060777 | ATP6V0E1 |
| 20 | 4710001 | 4760001 | 55.060777 | RPL26L1 |
| 2 | 15675278 | 15725278 | 55.055914 | UBE2E3 |
| 20 | 10010001 | 10060001 | 55.036755 | BDP1 |
| 5 | 39272009 | 39322009 | 55.030924 | PDZRN4 |
| 16 | 46300477 | 46350477 | 54.948663 | CAMTA1 |
| 10 | 9160336 | 9210336 | 54.919236 | AP3B1 |
| 2 | 26125278 | 26175278 | 54.913169 | MYO3B |
| 26 | 13012988 | 13062988 | 54.627085 | HECTD2 |
| 2 | 4875278 | 4925278 | 54.508896 | MYO7B |
| 2 | 4875278 | 4925278 | 54.508896 | GPR17 |
| 2 | 4875278 | 4925278 | 54.508896 | LIMS2 |
| 15 | 37980381 | 38030381 | 54.413148 | PDE3B |
| 15 | 35130381 | 35180381 | 54.385806 | NUCB2 |
| 15 | 35130381 | 35180381 | 54.385806 | NCR3LG1 |
| 8 | 26622087 | 26672087 | 54.379133 | SH3GL2 |
| 13 | 11900789 | 11950789 | 54.339635 | CAMK1D |
| 25 | 2354306 | 2404306 | 54.277505 | PKMYT1 |
| 25 | 2354306 | 2404306 | 54.277505 | PAQR4 |
| 25 | 2354306 | 2404306 | 54.277505 | KREMEN2 |
| 25 | 2354306 | 2404306 | 54.277505 | FLYWCH1 |
| 11 | 11801746 | 11851746 | 54.195983 | EXOC6B |
| 3 | 24203417 | 24253417 | 54.10106 | TBX15 |
| 9 | 65707723 | 65757723 | 53.872377 | THEMIS |
| 8 | 4222087 | 4272087 | 53.806451 | GALNTL6 |
| 23 | 11500117 | 11550117 | 53.727259 | MDGA1 |
| 5 | 2322009 | 2372009 | 53.550583 | TRHDE |
| 7 | 53115379 | 53165379 | 53.474384 | NDFIP1 |
| 26 | 23562988 | 23612988 | 53.422941 | CNNM2 |
| 2 | 61575278 | 61625278 | 53.385355 | UBXN4 |
| 2 | 61575278 | 61625278 | 53.385355 | LCT |
| 20 | 5760001 | 5810001 | 53.235321 | NSG2 |
| 23 | 9800117 | 9850117 | 53.216387 | SRPK1 |
| 29 | 17126202 | 17176202 | 53.070748 | TENM4 |
| 13 | 11750789 | 11800789 | 52.960546 | TRNAE-UUC |
| 13 | 11750789 | 11800789 | 52.960546 | CAMK1D |
| 4 | 27532185 | 27582185 | 52.855636 | HDAC9 |
| 5 | 48572009 | 48622009 | 52.771586 | LEMD3 |
| 5 | 27272009 | 27322009 | 52.519537 | KRT74 |
| 5 | 27272009 | 27322009 | 52.519537 | KRT72 |
| 5 | 27272009 | 27322009 | 52.519537 | KRT73 |
| 3 | 36153417 | 36203417 | 52.488139 | NTNG1 |
| 22 | 658988 | 708988 | 52.397156 | VOPP1 |
| 13 | 29250789 | 29300789 | 52.33108 | FAM107B |
| 16 | 3800477 | 3850477 | 52.20576 | RHEX |
| 23 | 3600117 | 3650117 | 52.135557 | DST |
| 8 | 4322087 | 4372087 | 52.081226 | GALNTL6 |
| 1 | 79879116 | 79929116 | 52.063957 | RTP4 |
| 19 | 5625961 | 5675961 | 52.060309 | MMD |
| 26 | 8112988 | 8162988 | 52.034121 | PRKG1 |
| 4 | 7432185 | 7482185 | 51.699652 | ABCA13 |
| 5 | 57672009 | 57722009 | 51.693299 | OR2AP1 |
| 16 | 34650477 | 34700477 | 51.660618 | PLD5 |
| 23 | 22300117 | 22350117 | 51.545586 | C23H6orf141 |
| 23 | 22300117 | 22350117 | 51.545586 | GLYATL3 |
| 18 | 26904930 | 26954930 | 51.469942 | TRNAW-CCA |
| 15 | 680381 | 730381 | 51.432406 | OR4C15 |
| 9 | 17057723 | 17107723 | 51.306956 | HTR1B |
| 15 | 5080381 | 5130381 | 51.266161 | DYNC2H1 |
| 20 | 32710001 | 32760001 | 51.065788 | OXCT1 |
| 18 | 44754930 | 44804930 | 50.984238 | GPI |
| 18 | 44754930 | 44804930 | 50.984238 | KIAA0355 |
| 5 | 20872009 | 20922009 | 50.969452 | EPYC |
| 24 | 24700927 | 24750927 | 50.897168 | GAREM1 |
| 8 | 10122087 | 10172087 | 50.869901 | FZD3 |
| 10 | 29610336 | 29660336 | 50.774185 | FMN1 |
| 29 | 17926202 | 17976202 | 50.655133 | ALG8 |
| 24 | 750927 | 800927 | 50.647196 | NFATC1 |
| 11 | 14801746 | 14851746 | 50.606215 | NLRC4 |
| 11 | 14801746 | 14851746 | 50.606215 | SLC30A6 |
| 29 | 6576202 | 6626202 | 50.582449 | GRM5 |
| 1 | 52429116 | 52479116 | 50.523626 | BBX |
| 14 | 45112803 | 45162803 | 50.382543 | COLEC10 |
| 1 | 19379116 | 19429116 | 50.372548 | BTG3 |
| 18 | 15854930 | 15904930 | 50.337656 | PHKB |
| 18 | 15854930 | 15904930 | 50.337656 | ITFG1 |
| 26 | 5412988 | 5462988 | 50.238162 | PCDH15 |
| 2 | 44225278 | 44275278 | 50.214933 | CACNB4 |
| 12 | 24814833 | 24864833 | 50.179388 | RFXAP |
| 9 | 39757723 | 39807723 | 50.165973 | DDO |
| 9 | 39757723 | 39807723 | 50.165973 | SLC22A16 |
| 1 | 74729116 | 74779116 | 50.159896 | FGF12 |
| 4 | 65332185 | 65382185 | 50.12541 | ADCYAP1R1 |
| 3 | 14253417 | 14303417 | 50.083933 | IQGAP3 |
| 3 | 14253417 | 14303417 | 50.083933 | TTC24 |
| 3 | 50603417 | 50653417 | 50.083498 | RPL5 |
| 3 | 50603417 | 50653417 | 50.083498 | FAM69A |
| 6 | 18150345 | 18200345 | 49.95249 | DKK2 |
| 4 | 60382185 | 60432185 | 49.800397 | ELMO1 |
| 9 | 49307723 | 49357723 | 49.759861 | ASCC3 |
| 29 | 1426202 | 1476202 | 49.70305 | DEUP1 |
| 6 | 50250345 | 50300345 | 49.567969 | PCDH7 |
| 16 | 35250477 | 35300477 | 49.447079 | FH |
| 10 | 33860336 | 33910336 | 49.445127 | SPRED1 |
| 16 | 2700477 | 2750477 | 49.384707 | NFASC |
| 13 | 12300789 | 12350789 | 49.349356 | UPF2 |
| 7 | 19315379 | 19365379 | 49.241919 | FEM1A |
| 7 | 19315379 | 19365379 | 49.241919 | TRNAG-CCC |
| 7 | 19315379 | 19365379 | 49.241919 | TICAM1 |
| 19 | 16875961 | 16925961 | 49.006415 | ASIC2 |
| 2 | 76325278 | 76375278 | 48.940158 | CNTNAP5 |
| 4 | 24032185 | 24082185 | 48.934437 | TRNAN-GUU |
| 4 | 24032185 | 24082185 | 48.934437 | MEOX2 |
| 18 | 29204930 | 29254930 | 48.850633 | TRNAA-AGC |
| 20 | 23510001 | 23560001 | 48.691245 | SLC38A9 |
| 4 | 63032185 | 63082185 | 48.607517 | MIR1814C |
| 7 | 21765379 | 21815379 | 48.578869 | RAD50 |
| 5 | 70972009 | 71022009 | 48.566671 | RTCB |
| 5 | 70972009 | 71022009 | 48.566671 | ASCL4 |
| 5 | 70972009 | 71022009 | 48.566671 | PRDM4 |
| 2 | 52575278 | 52625278 | 48.352881 | GTDC1 |
| 6 | 67900345 | 67950345 | 48.327031 | SPATA18 |
| 6 | 67900345 | 67950345 | 48.327031 | SGCB |
| 7 | 30165379 | 30215379 | 48.301838 | CSNK1G3 |
| 26 | 12512988 | 12562988 | 48.292482 | ANKRD1 |
| 26 | 12512988 | 12562988 | 48.292482 | RPP30 |
| 23 | 18700117 | 18750117 | 48.289198 | RUNX2 |
| 13 | 34550789 | 34600789 | 48.227051 | SVIL |
| 3 | 44603417 | 44653417 | 48.20827 | SNX7 |
| 10 | 4810336 | 4860336 | 48.135775 | LVRN |
| 27 | 21553840 | 21603840 | 48.113641 | TUSC3 |
| 6 | 44150345 | 44200345 | 48.086713 | DHX15 |
| 8 | 36122087 | 36172087 | 48.039081 | PTPRD |
| 7 | 33065379 | 33115379 | 47.98036 | PRR16 |
| 18 | 2054930 | 2104930 | 47.930155 | RFWD3 |
| 18 | 2054930 | 2104930 | 47.930155 | GLG1 |
| 4 | 40432185 | 40482185 | 47.899863 | CD36 |
| 11 | 12351746 | 12401746 | 47.779325 | CYP26B1 |
| 11 | 50201746 | 50251746 | 47.728719 | DNAH6 |
| 14 | 20312803 | 20362803 | 47.702069 | SNTG1 |
| 8 | 22872087 | 22922087 | 47.572592 | IFNAG |
| 8 | 22872087 | 22922087 | 47.572592 | KLHL9 |
| 13 | 51400789 | 51450789 | 47.504985 | HSPA12B |
| 13 | 51400789 | 51450789 | 47.504985 | C13H20orf27 |
| 13 | 51400789 | 51450789 | 47.504985 | SPEF1 |
| 13 | 51400789 | 51450789 | 47.504985 | CENPB |
| 13 | 51400789 | 51450789 | 47.504985 | CDC25B |
| 6 | 12600345 | 12650345 | 47.453527 | ANK2 |
| 11 | 11051746 | 11101746 | 47.436917 | ALMS1 |
| 4 | 25182185 | 25232185 | 47.393605 | BZW2 |
| 6 | 40150345 | 40200345 | 47.351896 | SLIT2 |
| 27 | 32953840 | 33003840 | 47.214484 | ZNF703 |
| 23 | 23500117 | 23550117 | 47.167248 | TRNAY-AUA |
| 10 | 64810336 | 64860336 | 47.16642 | SQOR |
| 5 | 24722009 | 24772009 | 47.162177 | FGD6 |
| 5 | 24722009 | 24772009 | 47.162177 | NR2C1 |
| 11 | 2001746 | 2051746 | 47.006785 | KCNIP3 |
| 11 | 2001746 | 2051746 | 47.006785 | PROM2 |
| 7 | 50115379 | 50165379 | 46.971513 | CTNNA1 |
| 7 | 19415379 | 19465379 | 46.906313 | TRNAG-CCC |
| 7 | 19415379 | 19465379 | 46.906313 | DPP9 |
| 7 | 19415379 | 19465379 | 46.906313 | TRNAG-UCC |
| 7 | 24165379 | 24215379 | 46.882313 | CHSY3 |
| 8 | 39222087 | 39272087 | 46.813822 | CD274 |
| 4 | 58332185 | 58382185 | 46.778916 | RGS2 |
| 28 | 1350777 | 1400777 | 46.721481 | ACTA1 |
| 8 | 13172087 | 13222087 | 46.707231 | MIR2285M-5 |
| 3 | 27353417 | 27403417 | 46.536559 | SLC22A15 |
| 4 | 23982185 | 24032185 | 46.429472 | MEOX2 |
| 20 | 36660001 | 36710001 | 46.372822 | WDR70 |
| 3 | 15253417 | 15303417 | 46.345476 | RUSC1 |
| 3 | 15253417 | 15303417 | 46.345476 | ASH1L |
| 1 | 74779116 | 74829116 | 46.267039 | FGF12 |
| 13 | 33450789 | 33500789 | 46.258314 | ARHGAP12 |
| 28 | 16050777 | 16100777 | 46.223647 | ANK3 |
| 4 | 10882185 | 10932185 | 46.167752 | MIR378-2 |
| 4 | 10882185 | 10932185 | 46.167752 | CALCR |
| 5 | 9422009 | 9472009 | 45.968743 | PPP1R12A |
| 5 | 10872009 | 10922009 | 45.904978 | PPFIA2 |
| 3 | 62903417 | 62953417 | 45.889079 | ADGRL2 |
| 14 | 46162803 | 46212803 | 45.846629 | EXT1 |
| 19 | 18675961 | 18725961 | 45.817971 | OMG |
| 19 | 18675961 | 18725961 | 45.817971 | EVI2B |
| 7 | 45665379 | 45715379 | 45.78013 | VDAC1 |
| 24 | 20400927 | 20450927 | 45.619344 | FHOD3 |
| 23 | 11550117 | 11600117 | 45.606394 | MDGA1 |
| 16 | 3500477 | 3550477 | 45.535876 | SLC41A1 |
| 16 | 3500477 | 3550477 | 45.535876 | RAB29 |
| 11 | 32451746 | 32501746 | 45.520055 | NRXN1 |
| 7 | 68165379 | 68215379 | 45.483704 | HAVCR1 |
| 1 | 24829116 | 24879116 | 45.459483 | ROBO2 |
| 18 | 11454930 | 11504930 | 45.456151 | GSE1 |
| 4 | 42382185 | 42432185 | 45.443424 | MAGI2 |
| 4 | 63932185 | 63982185 | 45.422183 | AVL9 |
| 10 | 62010336 | 62060336 | 45.387168 | DUT |
| 7 | 33215379 | 33265379 | 45.220737 | PRR16 |
| 16 | 24050477 | 24100477 | 45.119289 | MARK1 |
| 10 | 28260336 | 28310336 | 45.105433 | SLC12A6 |
| 10 | 28260336 | 28310336 | 45.105433 | NOP10 |
| 10 | 28260336 | 28310336 | 45.105433 | NUTM1 |
| 10 | 28260336 | 28310336 | 45.105433 | LPCAT4 |
| 2 | 71275278 | 71325278 | 45.054878 | SCTR |
| 12 | 21964833 | 22014833 | 45.029471 | FOXO1 |
| 13 | 35050789 | 35100789 | 45.018554 | JCAD |
| 3 | 9803417 | 9853417 | 44.954322 | SLAMF8 |
| 3 | 9803417 | 9853417 | 44.954322 | VSIG8 |
| 3 | 9803417 | 9853417 | 44.954322 | CFAP45 |
| 17 | 1818957 | 1868957 | 44.945614 | TLL1 |
| 23 | 11300117 | 11350117 | 44.909757 | CMTR1 |
| 5 | 10372009 | 10422009 | 44.882322 | LIN7A |
| 14 | 16712803 | 16762803 | 44.829281 | DERL1 |
| 11 | 38301746 | 38351746 | 44.790087 | PNPT1 |
| 2 | 15175278 | 15225278 | 44.770941 | ITGA4 |
| 3 | 29903417 | 29953417 | 44.72633 | MAGI3 |
| 5 | 20972009 | 21022009 | 44.629558 | DCN |
| 5 | 20972009 | 21022009 | 44.629558 | LUM |
| 10 | 51260336 | 51310336 | 44.619777 | RNF111 |
| 24 | 9400927 | 9450927 | 44.58202 | DSEL |
| 15 | 7980381 | 8030381 | 44.576557 | ARHGAP42 |
| 8 | 26022087 | 26072087 | 44.575605 | ADAMTSL1 |
| 17 | 35718957 | 35768957 | 44.498338 | TRPC3 |
| 5 | 4472009 | 4522009 | 44.412767 | KCNC2 |
| 18 | 40404930 | 40454930 | 44.329838 | CCNE1 |
| 11 | 40201746 | 40251746 | 44.327939 | TRNAC-GCA |
| 1 | 2829116 | 2879116 | 44.310363 | SYNJ1 |
| 1 | 57729116 | 57779116 | 44.300161 | NEPRO |
| 1 | 57729116 | 57779116 | 44.300161 | GTPBP8 |
| 3 | 23503417 | 23553417 | 44.258181 | HMGCS2 |
| 3 | 23503417 | 23553417 | 44.258181 | REG4 |
| 13 | 29450789 | 29500789 | 44.251125 | HSPA14 |
| 13 | 29450789 | 29500789 | 44.251125 | CDNF |
| 2 | 26325278 | 26375278 | 44.220516 | UBR3 |
| 2 | 17175278 | 17225278 | 44.097364 | ZNF385B |
| 5 | 11272009 | 11322009 | 44.095291 | PPFIA2 |
| 7 | 14515379 | 14565379 | 44.028341 | COL5A3 |
| 7 | 14515379 | 14565379 | 44.028341 | TRNAD-GUC |
| 1 | 58729116 | 58779116 | 44.001194 | QTRT2 |
| 1 | 58729116 | 58779116 | 44.001194 | CCDC191 |
| 1 | 58679116 | 58729116 | 43.883296 | CCDC191 |
| 24 | 7500927 | 7550927 | 43.762107 | DOK6 |
| 4 | 60782185 | 60832185 | 43.75822 | ANLN |
| 2 | 25075278 | 25125278 | 43.756609 | METTL8 |
| 27 | 3053840 | 3103840 | 43.544452 | CSMD1 |
| 19 | 32125961 | 32175961 | 43.540986 | COX10 |
| 22 | 18908988 | 18958988 | 43.501268 | GRM7 |
| 7 | 47565379 | 47615379 | 43.432419 | SMAD5 |
| 18 | 39504930 | 39554930 | 43.400289 | CHST4 |
| 18 | 14854930 | 14904930 | 43.382741 | SHCBP1 |
| 11 | 1351746 | 1401746 | 43.365848 | ACOXL |
| 15 | 23580381 | 23630381 | 43.287718 | NCAM1 |
| 16 | 46400477 | 46450477 | 43.274769 | CAMTA1 |
| 3 | 12553417 | 12603417 | 43.263571 | FCRL1 |
| 7 | 40565379 | 40615379 | 43.155704 | ZNF496 |
| 25 | 8604306 | 8654306 | 43.084447 | GRIN2A |
| 12 | 21464833 | 21514833 | 43.078694 | NEK5 |
| 27 | 26553840 | 26603840 | 43.065428 | MIR2399 |
| 27 | 26553840 | 26603840 | 43.065428 | RBPMS |
| 7 | 6865379 | 6915379 | 43.06451 | TPM4 |
| 7 | 6865379 | 6915379 | 43.06451 | RAB8A |
| 7 | 6865379 | 6915379 | 43.06451 | HSH2D |
| 25 | 11354306 | 11404306 | 43.038418 | CPPED1 |
| 21 | 31812447 | 31862447 | 43.037079 | SCAPER |
| 2 | 60675278 | 60725278 | 42.974183 | TRNAC-GCA |
| 12 | 21664833 | 21714833 | 42.974012 | TRNAM-CAU |
| 12 | 21664833 | 21714833 | 42.974012 | THSD1 |
| 2 | 23975278 | 24025278 | 42.957843 | TRNAG-UCC |
| 14 | 1162803 | 1212803 | 42.934272 | ZC3H3 |
| 9 | 28207723 | 28257723 | 42.898707 | CLVS2 |
| 18 | 39254930 | 39304930 | 42.82276 | AP1G1 |
| 28 | 15750777 | 15800777 | 42.773639 | ANK3 |
| 11 | 11601746 | 11651746 | 42.653672 | EXOC6B |
| 9 | 43307723 | 43357723 | 42.642891 | RTN4IP1 |
| 9 | 43307723 | 43357723 | 42.642891 | QRSL1 |
| 6 | 34350345 | 34400345 | 42.592104 | CCSER1 |
| 8 | 15272087 | 15322087 | 42.574885 | LINGO2 |
| 18 | 4604930 | 4654930 | 42.546336 | ADAMTS18 |
| 27 | 33203840 | 33253840 | 42.482972 | EIF4EBP1 |
| 27 | 33203840 | 33253840 | 42.482972 | ADRB3 |
| 27 | 33203840 | 33253840 | 42.482972 | TRNAC-GCA |

**Table S2**. A summary of genes from Fst (*P*-value < 0.5%) between Yanbian and N'Dama cattle

| CHROM | START | END | Fst | GENE |
| --- | --- | --- | --- | --- |
| 1 | 1.43E+08 | 1.43E+08 | 0.600909 | CBS\|U2AF1 |
| 1 | 56540001 | 56710000 | 0.721453 | PHLDB2 |
| 1 | 76380001 | 76470000 | 0.714669 | GMNC |
| 1 | 1.07E+08 | 1.07E+08 | 0.662466 | IFT80\|C1H3orf80 |
| 1 | 33280001 | 33430000 | 0.777678 | CADM2 |
| 1 | 1.13E+08 | 1.13E+08 | 0.746683 | MME |
| 1 | 33180001 | 33270000 | 0.781597 | CADM2 |
| 1 | 1060001 | 1130000 | 0.611802 | LOC101903444\|C1H21orf140\|SMIM11A\|KCNE2 |
| 1 | 1.35E+08 | 1.35E+08 | 0.636867 | AMOTL2 |
| 1 | 1.08E+08 | 1.08E+08 | 0.786587 | SCHIP1 |
| 1 | 26420001 | 26470000 | 0.606684 | ROBO1 |
| 1 | 1.07E+08 | 1.07E+08 | 0.596324 | TRIM59\|SMC4\|IFT80 |
| 1 | 5780001 | 5870000 | 0.782378 | CLDN17 |
| 1 | 1.41E+08 | 1.41E+08 | 0.67456 | DSCAM |
| 1 | 2140001 | 2230000 | 0.85163 | IFNAR1 |
| 1 | 1.26E+08 | 1.26E+08 | 0.737513 | U2SURP\|PAQR9 |
| 1 | 1.25E+08 | 1.25E+08 | 0.791277 | SLC9A9 |
| 1 | 1.06E+08 | 1.06E+08 | 0.600875 | SPTSSB |
| 1 | 93540001 | 93590000 | 0.596894 | NLGN1 |
| 1 | 1.55E+08 | 1.55E+08 | 0.9459 | SATB1 |
| 1 | 1.3E+08 | 1.3E+08 | 0.683609 | RBP2 |
| 1 | 68940001 | 68990000 | 0.625035 | KALRN |
| 1 | 1.22E+08 | 1.22E+08 | 0.624808 | PLSCR4 |
| 1 | 1.13E+08 | 1.13E+08 | 0.624995 | GPR149 |
| 1 | 68540001 | 68630000 | 0.758159 | KALRN |
| 1 | 1.25E+08 | 1.25E+08 | 0.762986 | SLC9A9 |
| 1 | 82100001 | 82150000 | 0.653274 | C1H3orf70\|VPS8 |
| 1 | 1.18E+08 | 1.18E+08 | 0.679861 | TSC22D2 |
| 1 | 1.15E+08 | 1.16E+08 | 0.705344 | MBNL1 |
| 1 | 35060001 | 35150000 | 0.702954 | VGLL3\|LOC101902812 |
| 1 | 67620001 | 67690000 | 0.656633 | SEC22A\|ADCY5 |
| 1 | 99820001 | 99910000 | 0.851102 | WDR49 |
| 2 | 1120001 | 1190000 | 0.623644 | CYFIP1 |
| 2 | 1.34E+08 | 1.34E+08 | 0.613857 | KLHDC7A |
| 2 | 1.2E+08 | 1.2E+08 | 0.695763 | NPPC\|DIS3L2 |
| 2 | 75900001 | 75970000 | 0.640874 | CNTNAP5 |
| 2 | 1.04E+08 | 1.04E+08 | 0.782843 | MREG\|TMEM169\|PECR |
| 2 | 62800001 | 62890000 | 0.740425 | MGAT5 |
| 2 | 38160001 | 38230000 | 0.643856 | CCDC148\|UPP2 |
| 2 | 61820001 | 61950000 | 0.781758 | ZRANB3 |
| 2 | 31040001 | 31110000 | 0.630949 | SCN3A |
| 2 | 58420001 | 58510000 | 0.794515 | NXPH2 |
| 2 | 37680001 | 37850000 | 0.769291 | PKP4 |
| 2 | 90720001 | 90790000 | 0.705327 | KIAA2012\|SUMO1\|NOP58 |
| 2 | 1.36E+08 | 1.36E+08 | 0.675181 | SRARP |
| 2 | 34560001 | 34610000 | 0.637235 | SLC4A10 |
| 2 | 25500001 | 25630000 | 0.767998 | GORASP2\|LOC101905343\|GAD1 |
| 2 | 6940001 | 6990000 | 0.619675 | WDR75 |
| 2 | 58540001 | 58690000 | 0.657667 | SPOPL |
| 2 | 22720001 | 22830000 | 0.81276 | SP3 |
| 2 | 35740001 | 36090000 | 0.837189 | RBMS1\|LOC101906513 |
| 2 | 79800001 | 79890000 | 0.792588 | MYO1B |
| 2 | 1.12E+08 | 1.12E+08 | 0.741626 | AP1S3\|WDFY1 |
| 2 | 97940001 | 98010000 | 0.641013 | KANSL1L\|ACADL |
| 2 | 37960001 | 38010000 | 0.630605 | CCDC148 |
| 2 | 91100001 | 91270000 | 0.722045 | FAM117B\|ICA1L\|WDR12\|CARF |
| 2 | 1.36E+08 | 1.36E+08 | 0.772918 | FBXO42\|CPLANE2\|ARHGEF19\|EPHA2 |
| 2 | 26360001 | 26450000 | 0.625748 | UBR3 |
| 2 | 30940001 | 31030000 | 0.717036 | SCN2A |
| 2 | 98400001 | 98470000 | 0.620125 | CPS1 |
| 2 | 97020001 | 97110000 | 0.748734 | LOC112442978\|LOC616092 |
| 2 | 1.02E+08 | 1.02E+08 | 0.704892 | SPAG16 |
| 2 | 38420001 | 38510000 | 0.802597 | ACVR1 |
| 2 | 99780001 | 99850000 | 0.642481 | ERBB4 |
| 2 | 6280001 | 6350000 | 0.747525 | MSTN |
| 2 | 25280001 | 25350000 | 0.631872 | TLK1 |
| 2 | 6160001 | 6210000 | 0.618339 | C2H2orf88 |
| 2 | 34460001 | 34530000 | 0.694679 | DPP4\|SLC4A10 |
| 2 | 90860001 | 90970000 | 0.816361 | BMPR2 |
| 2 | 60400001 | 60450000 | 0.623984 | THSD7B |
| 2 | 9020001 | 9070000 | 0.617829 | CALCRL |
| 3 | 67480001 | 67590000 | 0.695246 | PIGK |
| 3 | 800001 | 850000 | 0.602856 | ADCY10 |
| 3 | 73140001 | 73210000 | 0.679159 | NEGR1 |
| 3 | 59720001 | 59790000 | 0.634091 | UOX\|SAMD13\|SAMD13 |
| 3 | 1.14E+08 | 1.14E+08 | 0.63241 | HJURP |
| 3 | 49040001 | 49130000 | 0.638328 | ABCD3 |
| 3 | 49200001 | 49270000 | 0.657822 | ARHGAP29 |
| 3 | 79100001 | 79150000 | 0.606001 | PDE4B |
| 3 | 75020001 | 75090000 | 0.685591 | LRRC7 |
| 3 | 86440001 | 86510000 | 0.723014 | FGGY |
| 3 | 99260001 | 99310000 | 0.599184 | LOC784417 |
| 3 | 11640001 | 11690000 | 0.609521 | LOC507767 |
| 3 | 33720001 | 33790000 | 0.612939 | GSTM2\|LOC100295687\|LOC107132300\|GSTM1 |
| 3 | 1.15E+08 | 1.15E+08 | 0.63534 | AGAP1 |
| 3 | 10520001 | 10570000 | 0.597133 | ACKR1\|CADM3 |
| 3 | 42880001 | 42950000 | 0.67809 | CDC14A |
| 3 | 79980001 | 80030000 | 0.637103 | DNAJC6 |
| 3 | 1.12E+08 | 1.12E+08 | 0.608499 | CSMD2 |
| 3 | 89380001 | 89550000 | 0.783814 | FYB2\|PRKAA2 |
| 3 | 18940001 | 19030000 | 0.631023 | C2CD4D\|RORC\|LINGO4\|TDRKH\|OAZ3\|MRPL9 |
| 3 | 35280001 | 35330000 | 0.61206 | VAV3 |
| 3 | 57220001 | 57270000 | 0.622415 | SH3GLB1 |
| 3 | 99100001 | 99230000 | 0.654001 | LOC100847677\|CYP4A11 |
| 3 | 1.18E+08 | 1.18E+08 | 0.692159 | TRAF3IP1\|ASB1 |
| 3 | 14280001 | 14350000 | 0.669925 | IQGAP3\|MEF2D |
| 3 | 19040001 | 19190000 | 0.903381 | RIIAD1\|CELF3\|SNX27 |
| 3 | 14360001 | 14430000 | 0.602185 | MEF2D |
| 3 | 87480001 | 87550000 | 0.701863 | OMA1 |
| 4 | 63340001 | 63410000 | 0.603898 | BBS9 |
| 4 | 79300001 | 79350000 | 0.595359 | INHBA |
| 4 | 53460001 | 53650000 | 0.676374 | MDFIC |
| 4 | 19980001 | 20050000 | 0.651887 | THSD7A |
| 4 | 1.11E+08 | 1.11E+08 | 0.635601 | CNTNAP2 |
| 4 | 19240001 | 19350000 | 0.678983 | PHF14 |
| 4 | 36200001 | 36290000 | 0.664907 | SEMA3A |
| 4 | 77360001 | 77410000 | 0.600063 | COA1 |
| 4 | 78000001 | 78070000 | 0.66676 | HECW1 |
| 4 | 77560001 | 77650000 | 0.709262 | HECW1 |
| 4 | 1.11E+08 | 1.11E+08 | 0.756108 | CNTNAP2 |
| 4 | 78080001 | 78130000 | 0.637844 | MRPL32\|PSMA2 |
| 4 | 77840001 | 77930000 | 0.708682 | HECW1 |
| 4 | 66960001 | 67010000 | 0.629553 | CHN2 |
| 4 | 94500001 | 94550000 | 0.626927 | KLF14 |
| 4 | 55320001 | 55410000 | 0.62677 | GPR85 |
| 4 | 79000001 | 79090000 | 0.731683 | GLI3 |
| 4 | 97520001 | 97570000 | 0.606417 | EXOC4 |
| 4 | 1.05E+08 | 1.05E+08 | 0.646922 | KIAA1147\|WEE2 |
| 4 | 5840001 | 5910000 | 0.729951 | ZPBP |
| 4 | 92280001 | 92330000 | 0.611484 | SND1 |
| 4 | 70980001 | 71110000 | 0.802517 | OSBPL3\|LOC100299757\|GSDME |
| 4 | 87360001 | 87550000 | 0.813835 | TAS2R16\|SLC13A1 |
| 4 | 40420001 | 40490000 | 0.681801 | CD36 |
| 5 | 27040001 | 27110000 | 0.624606 | KRT8\|KRT78\|KRT79 |
| 5 | 1160001 | 1250000 | 0.717182 | LGR5\|ZFC3H1 |
| 5 | 97360001 | 97410000 | 0.598929 | BORCS5 |
| 5 | 43840001 | 43950000 | 0.70949 | LRRC10\|CCT2\|FRS2 |
| 5 | 1.06E+08 | 1.06E+08 | 0.71264 | PARP11\|CRACR2A |
| 5 | 77560001 | 77690000 | 0.719521 | BICD1 |
| 5 | 65680001 | 65730000 | 0.668162 | LOC101902154\|DRAM1 |
| 5 | 1.03E+08 | 1.03E+08 | 0.747899 | C1RL\|LOC112446756 |
| 5 | 1.04E+08 | 1.04E+08 | 0.640519 | ANO2 |
| 5 | 94380001 | 94450000 | 0.648029 | PTPRO |
| 5 | 26780001 | 26930000 | 0.743926 | RARG\|ITGB7\|ZNF740\|CSAD\|SOAT2\|IGFBP6\|SPRYD3\|TNS2\|EIF4B |
| 5 | 1.07E+08 | 1.07E+08 | 0.63458 | NRIP2\|ITFG2\|FKBP4\|DDX11 |
| 5 | 88120001 | 88190000 | 0.616437 | CMAS |
| 5 | 74480001 | 74570000 | 0.718745 | LOC788334\|LOC524576\|LOC510193 |
| 5 | 38080001 | 38130000 | 0.613707 | PRICKLE1 |
| 5 | 1.15E+08 | 1.15E+08 | 0.877311 | PARVG\|SHISAL1 |
| 5 | 1.06E+08 | 1.06E+08 | 0.659323 | FGF23\|TIGAR\|CCND2 |
| 5 | 31940001 | 31990000 | 0.595528 | ZNF641\|LOC784038\|H1FNT |
| 5 | 96100001 | 96190000 | 0.638054 | GRIN2B |
| 5 | 1.09E+08 | 1.09E+08 | 0.687963 | CECR2\|ATP6V1E1 |
| 5 | 1380001 | 1430000 | 0.615799 | RAB21 |
| 5 | 10020001 | 10110000 | 0.690763 | PTPRQ |
| 5 | 24740001 | 24850000 | 0.676461 | NR2C1\|FGD6 |
| 5 | 1.03E+08 | 1.04E+08 | 0.736874 | C1S\|LPCAT3\|EMG1\|PHB2\|PTPN6 |
| 5 | 1.05E+08 | 1.05E+08 | 0.672059 | ANO2\|NTF3 |
| 5 | 9820001 | 9870000 | 0.636446 | OTOGL |
| 5 | 26060001 | 26150000 | 0.700686 | HOXC12\|HOXC13 |
| 5 | 25600001 | 25670000 | 0.749835 | GTSF1\|ITGA5 |
| 5 | 96860001 | 96950000 | 0.688111 | HEBP1\|GPRC5D |
| 6 | 29240001 | 29330000 | 0.761811 | UNC5C |
| 6 | 22620001 | 22770000 | 0.728625 | BANK1 |
| 6 | 4380001 | 4430000 | 0.627223 | NDNF |
| 6 | 25920001 | 26010000 | 0.649486 | TSPAN5 |
| 6 | 19300001 | 19350000 | 0.646789 | GSTCD |
| 6 | 71940001 | 72030000 | 0.616329 | HOPX |
| 6 | 59300001 | 59370000 | 0.632262 | CHRNA9 |
| 6 | 62980001 | 63070000 | 0.702976 | KCTD8 |
| 6 | 16280001 | 16330000 | 0.6202 | COL25A1 |
| 6 | 31160001 | 31210000 | 0.625255 | GRID2 |
| 6 | 1.11E+08 | 1.11E+08 | 0.669206 | CD38 |
| 6 | 86900001 | 87070000 | 0.910695 | GC |
| 6 | 20640001 | 20730000 | 0.797515 | CXXC4 |
| 6 | 72360001 | 72450000 | 0.606996 | NOA1\|POLR2B\|IGFBP7 |
| 6 | 81160001 | 81210000 | 0.604359 | EPHA5 |
| 6 | 1.13E+08 | 1.13E+08 | 0.683489 | TBC1D14 |
| 6 | 81100001 | 81150000 | 0.628442 | EPHA5 |
| 6 | 1.11E+08 | 1.11E+08 | 0.640389 | PROM1 |
| 6 | 60960001 | 61050000 | 0.848257 | BEND4 |
| 6 | 1.17E+08 | 1.17E+08 | 0.616751 | CTBP1 |
| 6 | 87460001 | 87610000 | 0.759885 | ADAMTS3 |
| 6 | 99960001 | 1E+08 | 0.601703 | ARHGAP24 |
| 7 | 380001 | 450000 | 0.674315 | LOC100125913 |
| 7 | 1.1E+08 | 1.1E+08 | 0.660719 | TMEM232 |
| 7 | 88220001 | 88270000 | 0.630428 | MEF2C |
| 7 | 24580001 | 24670000 | 0.769908 | ADAMTS19 |
| 7 | 17340001 | 17390000 | 0.596198 | LOC100337044 |
| 7 | 18120001 | 18230000 | 0.682758 | ACER1\|MLLT1\|ACSBG2 |
| 7 | 17240001 | 17330000 | 0.726761 | ACTL9\|LOC107132616\|LOC101904981\|LOC112447353 |
| 7 | 40840001 | 40890000 | 0.628896 | GCSAML |
| 7 | 73000001 | 73050000 | 0.599168 | GABRB2 |
| 7 | 62360001 | 62430000 | 0.616719 | ANXA6 |
| 7 | 17720001 | 17770000 | 0.606622 | VAV1\|SH2D3A\|TRIP10\|GPR108 |
| 7 | 90580001 | 90630000 | 0.600423 | ADGRV1 |
| 7 | 40660001 | 40830000 | 0.658129 | NLRP3\|LOC516409\|OR2B11\|LOC112447546\|LOC522955\|LOC613390\|OR2C3 |
| 7 | 39680001 | 39810000 | 0.77681 | COL23A1\|CLK4\|ZNF354A |
| 7 | 1.08E+08 | 1.08E+08 | 0.71005 | LOC112447569 |
| 7 | 56880001 | 56990000 | 0.707154 | PRELID2 |
| 7 | 49120001 | 49170000 | 0.612776 | KLHL3 |
| 7 | 57000001 | 57070000 | 0.661636 | PRELID2\|GRXCR2 |
| 7 | 56780001 | 56850000 | 0.685996 | PRELID2 |
| 7 | 57520001 | 57590000 | 0.733096 | POU4F3 |
| 7 | 60540001 | 60610000 | 0.685764 | ABLIM3 |
| 7 | 67880001 | 67930000 | 0.62503 | SGCD |
| 7 | 18480001 | 18550000 | 0.630033 | FUT6\|NRTN\|DUS3L\|PRR22 |
| 7 | 4960001 | 5030000 | 0.600233 | CIST1\|LOC618787\|PDE4C\|RAB3A\|MPV17L2 |
| 7 | 40900001 | 41010000 | 0.675693 | GCSAML\|OR2G2\|LOC508626\|OR2G3\|LOC524985\|LOC787559\|LOC532075 |
| 7 | 45720001 | 46030000 | 0.809832 | TCF7\|SKP1\|PPP2CA\|CDKL3\|UBE2B\|CDKN2AIPNL |
| 7 | 42980001 | 43050000 | 0.642105 | PLPP2\|MIER2 |
| 7 | 18960001 | 19030000 | 0.636314 | PTPRS |
| 7 | 47320001 | 47390000 | 0.684144 | IL9\|FBXL21 |
| 7 | 79900001 | 79950000 | 0.600275 | TENM2 |
| 7 | 90660001 | 90750000 | 0.667841 | ADGRV1 |
| 7 | 40540001 | 40630000 | 0.682102 | ZNF496 |
| 7 | 6600001 | 6650000 | 0.636297 | EPS15L1 |
| 7 | 4620001 | 4790000 | 0.756961 | FKBP8\|ELL\|ISYNA1\|SSBP4\|LRRC25\|GDF15 |
| 8 | 5180001 | 5250000 | 0.644297 | GALNTL6 |
| 8 | 1.03E+08 | 1.03E+08 | 0.615596 | BSPRY\|HDHD3\|ALAD\|POLE3\|C8H9orf43 |
| 8 | 1280001 | 1390000 | 0.650915 | SH3RF1 |
| 8 | 52020001 | 52230000 | 0.753371 | PCSK5 |
| 8 | 65040001 | 65090000 | 0.608913 | ERP44 |
| 8 | 46480001 | 46530000 | 0.618602 | SMC5 |
| 8 | 10380001 | 10490000 | 0.734261 | ZNF395\|PNOC |
| 8 | 65980001 | 66050000 | 0.631836 | CNTNAP3 |
| 8 | 17260001 | 17330000 | 0.651451 | IFT74\|PLAA |
| 8 | 44120001 | 44190000 | 0.69181 | DOCK8 |
| 8 | 76180001 | 76250000 | 0.63521 | RASEF |
| 8 | 1.06E+08 | 1.06E+08 | 0.686048 | ASTN2 |
| 8 | 15240001 | 15290000 | 0.607327 | LINGO2 |
| 8 | 72440001 | 72590000 | 0.789766 | LOC100335268\|DOCK5 |
| 8 | 1.06E+08 | 1.06E+08 | 0.648462 | ASTN2\|TRIM32 |
| 8 | 54160001 | 54210000 | 0.633311 | CEP78 |
| 8 | 8520001 | 8570000 | 0.716935 | PINX1\|SOX7 |
| 8 | 64220001 | 64270000 | 0.603306 | ALG2\|SEC61B |
| 8 | 78340001 | 78390000 | 0.637904 | NTRK2 |
| 8 | 44240001 | 44290000 | 0.656511 | DOCK8 |
| 8 | 1.11E+08 | 1.11E+08 | 0.702772 | CNTRL\|RAB14 |
| 8 | 1.11E+08 | 1.11E+08 | 0.630502 | ADI1\|TRAPPC12\|EIPR1 |
| 8 | 23700001 | 23790000 | 0.732358 | FOCAD\|MLLT3 |
| 8 | 50840001 | 50930000 | 0.841446 | TRPM6 |
| 8 | 84840001 | 84910000 | 0.624757 | FAM120A\|PHF2 |
| 8 | 39260001 | 39310000 | 0.615366 | CD274\|PLGRKT |
| 8 | 42460001 | 42550000 | 0.679522 | SMARCA2 |
| 8 | 7480001 | 7530000 | 0.605759 | DEFB136 |
| 8 | 1.01E+08 | 1.01E+08 | 0.769309 | DNAJC25\|GNG10\|C8H9orf84 |
| 9 | 52860001 | 52950000 | 0.782311 | NDUFAF4 |
| 9 | 11220001 | 11270000 | 0.596897 | RIMS1 |
| 9 | 23640001 | 23690000 | 0.597069 | SNAP91 |
| 9 | 95080001 | 95130000 | 0.623219 | SYTL3 |
| 9 | 23320001 | 23370000 | 0.595754 | ME1 |
| 9 | 68300001 | 68350000 | 0.607396 | SAMD3 |
| 9 | 41540001 | 41610000 | 0.682024 | FOXO3 |
| 9 | 12320001 | 12370000 | 0.638045 | KCNQ5 |
| 9 | 31820001 | 31890000 | 0.703208 | MAN1A1 |
| 9 | 86640001 | 86690000 | 0.62781 | TAB2 |
| 9 | 74860001 | 74990000 | 0.771363 | PEX7\|SLC35D3\|IL20RA |
| 9 | 1.03E+08 | 1.04E+08 | 0.701766 | WDR27\|C9H6orf120\|PHF10\|TCTE3\|LOC104969678\|ERMARD |
| 9 | 49400001 | 49470000 | 0.649162 | ASCC3 |
| 9 | 25500001 | 25570000 | 0.741733 | NCOA7\|HEY2 |
| 9 | 11520001 | 11570000 | 0.638221 | RIMS1 |
| 9 | 1.04E+08 | 1.04E+08 | 0.61758 | FAM120B\|PSMB1 |
| 9 | 43240001 | 43290000 | 0.603359 | QRSL1 |
| 9 | 97520001 | 97570000 | 0.596528 | PRKN |
| 10 | 45580001 | 45650000 | 0.639482 | ZNF609\|TRIP4\|PCLAF |
| 10 | 70900001 | 70950000 | 0.597068 | DACT1 |
| 10 | 22320001 | 22370000 | 0.599366 | LOC100336282 |
| 10 | 26640001 | 26690000 | 0.619291 | LOC790312\|PIP4P1\|APEX1\|OSGEP |
| 10 | 79660001 | 79710000 | 0.630556 | ZFYVE26\|RAD51B |
| 10 | 38420001 | 38470000 | 0.596466 | EPB42 |
| 10 | 61440001 | 61510000 | 0.673434 | CEP152 |
| 10 | 20900001 | 20950000 | 0.603418 | TGM1\|TINF2\|GMPR2\|NEDD8\|MDP1\|CHMP4A |
| 10 | 35080001 | 35210000 | 0.793373 | THBS1 |
| 10 | 10860001 | 10990000 | 0.660443 | MTX3 |
| 10 | 36500001 | 36550000 | 0.634051 | INO80 |
| 10 | 85440001 | 85590000 | 0.749676 | ALDH6A1\|LIN52 |
| 10 | 25560001 | 25670000 | 0.835655 | LOC101904911\|OR10G2\|LOC101904323\|LOC107132841\|SALL2\|METTL3 |
| 10 | 45420001 | 45530000 | 0.669447 | ZNF609\|LOC101907658 |
| 10 | 36560001 | 36630000 | 0.663878 | INO80 |
| 10 | 81520001 | 81650000 | 0.658113 | SLC10A1\|SMOC1 |
| 10 | 44640001 | 44750000 | 0.674009 | GNG2 |
| 10 | 77540001 | 77650000 | 0.644353 | FUT8 |
| 10 | 66820001 | 66970000 | 0.748604 | CDKN3\|CNIH1 |
| 10 | 20320001 | 20430000 | 0.679295 | REC114\|NPTN |
| 10 | 1720001 | 1770000 | 0.611343 | EPB41L4A |
| 10 | 14480001 | 14550000 | 0.767685 | MAP2K5 |
| 10 | 86760001 | 86830000 | 0.699504 | FLVCR2 |
| 11 | 72540001 | 72590000 | 0.689948 | KHK\|EMILIN1\|OST4\|AGBL5 |
| 11 | 80960001 | 81010000 | 0.610491 | VSNL1 |
| 11 | 94540001 | 94590000 | 0.595395 | DENND1A |
| 11 | 1.04E+08 | 1.04E+08 | 0.73283 | LOC112448856 |
| 11 | 99520001 | 99570000 | 0.618512 | MIGA2\|DOLPP1\|CRAT |
| 11 | 60620001 | 60670000 | 0.597387 | COMMD1 |
| 11 | 33160001 | 33210000 | 0.604822 | NRXN1 |
| 11 | 64720001 | 64970000 | 0.747187 | MEIS1 |
| 11 | 77700001 | 77890000 | 0.895527 | TDRD15\|APOB |
| 11 | 5100001 | 5210000 | 0.723298 | AFF3 |
| 11 | 72640001 | 72690000 | 0.632718 | MAPRE3\|DPYSL5 |
| 11 | 78340001 | 78410000 | 0.617227 | RHOB |
| 11 | 99080001 | 99130000 | 0.626459 | GLE1\|SPTAN1 |
| 11 | 63780001 | 63850000 | 0.658081 | SPRED2 |
| 11 | 45580001 | 45650000 | 0.685991 | ST6GAL2 |
| 11 | 59540001 | 59830000 | 0.768028 | C11H2orf74 |
| 11 | 70180001 | 70250000 | 0.670633 | ALK |
| 11 | 1.07E+08 | 1.07E+08 | 0.617082 | CACNA1B |
| 11 | 25520001 | 25630000 | 0.694301 | THADA |
| 11 | 94600001 | 94710000 | 0.800723 | DENND1A |
| 11 | 9780001 | 9830000 | 0.606708 | HK2 |
| 11 | 45760001 | 45810000 | 0.636722 | UXS1 |
| 11 | 27880001 | 28010000 | 0.722838 | SRBD1\|LOC526524 |
| 11 | 60340001 | 60430000 | 0.643284 | FAM161A |
| 11 | 89100001 | 89150000 | 0.607214 | LOC107132953 |
| 11 | 68840001 | 68910000 | 0.638497 | GALNT14 |
| 11 | 96720001 | 96770000 | 0.597428 | PBX3 |
| 11 | 70520001 | 70610000 | 0.689851 | ALK |
| 11 | 4380001 | 4490000 | 0.632283 | LYG1\|TXNDC9\|EIF5B |
| 11 | 59440001 | 59490000 | 0.60345 | LRRTM4 |
| 11 | 68160001 | 68510000 | 0.790473 | ANXA4\|GMCL1\|SNRNP27\|MXD1\|ASPRV1\|PCBP1\|C11H2orf42\|TIA1 |
| 11 | 1.04E+08 | 1.04E+08 | 0.611579 | LOC787891\|GPSM1 |
| 12 | 2260001 | 2350000 | 0.683394 | DIAPH3 |
| 12 | 32820001 | 32890000 | 0.617334 | GPR12 |
| 12 | 28500001 | 28610000 | 0.636024 | N4BP2L2\|N4BP2L1 |
| 12 | 30800001 | 30850000 | 0.628083 | UBL3 |
| 12 | 86980001 | 87070000 | 0.651368 | RASA3\|LOC112449103\|LOC100848072\|CDC16\|UPF3A |
| 12 | 2580001 | 2630000 | 0.640705 | DIAPH3 |
| 12 | 21120001 | 21190000 | 0.669886 | WDFY2 |
| 12 | 13640001 | 13710000 | 0.604551 | ENOX1 |
| 12 | 43940001 | 44030000 | 0.721908 | KLHL1 |
| 12 | 84600001 | 84690000 | 0.792713 | IRS2 |
| 13 | 70100001 | 70170000 | 0.659952 | CHD6 |
| 13 | 57340001 | 57390000 | 0.598477 | PRELID3B\|ATP5F1E\|TUBB1\|CTSZ |
| 13 | 79360001 | 79410000 | 0.624221 | NFATC2 |
| 13 | 24000001 | 24070000 | 0.625933 | ARMC3 |
| 13 | 3640001 | 3710000 | 0.61907 | MKKS\|SLX4IP |
| 13 | 57520001 | 57570000 | 0.622028 | GNAS\|LOC112449315 |
| 13 | 1180001 | 1310000 | 0.832913 | PLCB1 |
| 13 | 43560001 | 43610000 | 0.595719 | LOC112449293\|AKR1C3 |
| 13 | 3720001 | 3770000 | 0.607793 | SLX4IP |
| 13 | 77880001 | 77950000 | 0.630632 | SLC9A8 |
| 13 | 49120001 | 49250000 | 0.672359 | BMP2 |
| 13 | 23720001 | 23790000 | 0.684366 | PIP4K2A |
| 13 | 620001 | 710000 | 0.702381 | TMX4 |
| 13 | 62120001 | 62230000 | 0.779929 | COMMD7\|DNMT3B\|MAPRE1\|EFCAB8 |
| 13 | 78840001 | 78910000 | 0.651988 | ADNP\|DPM1\|MOCS3 |
| 14 | 43020001 | 43130000 | 0.77776 | MRPS28 |
| 14 | 76560001 | 76630000 | 0.663946 | ATP6V0D2\|PSKH2 |
| 14 | 52060001 | 52130000 | 0.701883 | LOC112449556 |
| 14 | 43160001 | 43210000 | 0.59964 | MRPS28 |
| 14 | 34120001 | 34170000 | 0.612491 | NCOA2 |
| 14 | 70040001 | 70110000 | 0.646789 | GEM |
| 14 | 15540001 | 15630000 | 0.613193 | MTSS1\|NDUFB9\|TATDN1\|RNF139\|TRMT12 |
| 14 | 70120001 | 70190000 | 0.636411 | CDH17 |
| 14 | 61540001 | 61590000 | 0.607438 | AZIN1 |
| 14 | 34860001 | 35010000 | 0.702513 | EYA1 |
| 14 | 77500001 | 77550000 | 0.629363 | LOC518422\|LOC506670 |
| 14 | 31260001 | 31370000 | 0.634285 | CSPP1\|ARFGEF1 |
| 14 | 26320001 | 26430000 | 0.693065 | CHD7 |
| 14 | 31040001 | 31150000 | 0.705048 | SGK3\|MCMDC2\|TCF24\|PPP1R42 |
| 14 | 520001 | 710000 | 0.787768 | SLC39A4\|CPSF1\|ADCK5\|SLC52A2\|FBXL6\|TMEM249\|SCRT1\|DGAT1\|HSF1\|BOP1\|SCX\|MROH1\|LOC112449560 |
| 14 | 23860001 | 24030000 | 0.763307 | IMPAD1 |
| 14 | 73220001 | 73410000 | 0.911863 | SLC26A7\|LRRC69\|OTUD6B\|PIP4P2 |
| 14 | 74480001 | 74550000 | 0.68006 | MMP16 |
| 14 | 43240001 | 43370000 | 0.849346 | TPD52 |
| 14 | 75280001 | 75390000 | 0.827327 | LOC787250 |
| 14 | 27120001 | 27210000 | 0.688521 | ASPH |
| 14 | 22220001 | 22290000 | 0.626072 | SOX17 |
| 14 | 36060001 | 36130000 | 0.712462 | KCNB2 |
| 15 | 46560001 | 46610000 | 0.627751 | TIMM10B\|ARFIP2\|TRIM3\|HPX |
| 15 | 55400001 | 55470000 | 0.605985 | UVRAG |
| 15 | 46440001 | 46510000 | 0.624886 | DCHS1\|TPP1\|TAF10\|ILK\|RRP8\|DNHD1 |
| 15 | 52760001 | 52830000 | 0.700254 | P2RY6\|ARHGEF17 |
| 15 | 55800001 | 55890000 | 0.711404 | GVQW3\|EMSY |
| 15 | 9520001 | 9570000 | 0.605864 | CNTN5 |
| 15 | 83000001 | 83070000 | 0.75588 | STX3\|MRPL16\|GIF\|TCN1 |
| 15 | 46040001 | 46110000 | 0.706287 | LOC100125776\|OR2AG1\|LOC112441631\|OR2AG2 |
| 15 | 15300001 | 15350000 | 0.629568 | ENDOD1 |
| 15 | 43080001 | 43150000 | 0.617452 | WEE1\|ZNF143 |
| 15 | 40600001 | 40670000 | 0.645957 | MICAL2 |
| 15 | 9340001 | 9430000 | 0.6822 | CNTN5 |
| 15 | 83100001 | 83170000 | 0.61462 | OOSP2\|MS4A3\|MS4A2 |
| 15 | 44620001 | 44670000 | 0.631034 | LOC782910 |
| 15 | 79860001 | 79970000 | 0.713382 | LOC615810\|OR9G9\|LOC509124\|LOC100301259 |
| 15 | 22540001 | 22590000 | 0.641905 | BCO2\|PTS |
| 15 | 83840001 | 83930000 | 0.657199 | LOC100271685 |
| 15 | 30840001 | 30950000 | 0.665565 | POU2F3\|TMEM136\|ARHGEF12 |
| 15 | 4280001 | 4330000 | 0.59839 | PDGFD |
| 15 | 64660001 | 64710000 | 0.621156 | CAPRIN1\|NAT10 |
| 15 | 82920001 | 82990000 | 0.663354 | LOC527077\|STX3 |
| 15 | 56660001 | 56730000 | 0.656795 | GDPD4 |
| 15 | 82240001 | 82310000 | 0.639776 | FAM111B\|DTX4 |
| 15 | 49720001 | 49810000 | 0.670163 | LOC788939\|LOC788640 |
| 15 | 15680001 | 15730000 | 0.648824 | AMOTL1 |
| 15 | 34180001 | 34250000 | 0.626094 | GRAMD1B\|SCN3B |
| 16 | 64020001 | 64070000 | 0.595722 | LAMC1 |
| 16 | 24200001 | 24270000 | 0.712872 | MARC2\|MARC1 |
| 16 | 21100001 | 21150000 | 0.643667 | GPATCH2\|SPATA17 |
| 16 | 47100001 | 47190000 | 0.758082 | GPR153\|HES3\|ICMT\|RNF207\|RPL22\|CHD5 |
| 16 | 26920001 | 26990000 | 0.667946 | CAPN8 |
| 16 | 64540001 | 64590000 | 0.60881 | SMG7 |
| 16 | 27200001 | 27250000 | 0.632205 | TP53BP2 |
| 16 | 17540001 | 17690000 | 0.733444 | LOC529930 |
| 16 | 27780001 | 27850000 | 0.748538 | CNIH3 |
| 16 | 42400001 | 42630000 | 0.728385 | MTOR\|ANGPTL7\|EXOSC10\|SRM\|MASP2\|TARDBP |
| 16 | 76520001 | 76590000 | 0.664841 | DENND1B |
| 16 | 37260001 | 37310000 | 0.603804 | SELP |
| 16 | 32640001 | 32710000 | 0.68485 | CATSPERE |
| 16 | 43180001 | 43250000 | 0.602863 | KIF1B |
| 16 | 66840001 | 66930000 | 0.732445 | HMCN1 |
| 16 | 18940001 | 18990000 | 0.600565 | USH2A |
| 16 | 38840001 | 38990000 | 0.675784 | FMO1\|FMO4\|PRRC2C |
| 16 | 37000001 | 37130000 | 0.753752 | NME7\|BLZF1\|CCDC181\|SLC19A2 |
| 16 | 71400001 | 71550000 | 0.707659 | DTL\|INTS7 |
| 16 | 63900001 | 63990000 | 0.692184 | DHX9\|SHCBP1L |
| 17 | 50940001 | 50990000 | 0.603411 | SCARB1 |
| 17 | 71640001 | 71690000 | 0.604864 | SPECC1L |
| 17 | 71740001 | 71810000 | 0.677007 | BCR\|LOC112441984\|RAB36 |
| 17 | 57100001 | 57190000 | 0.716327 | VSIG10\|WSB2\|RFC5 |
| 17 | 52800001 | 52870000 | 0.651694 | LOC101902760\|LOC527744\|KNTC1 |
| 17 | 67140001 | 67430000 | 0.76648 | MN1\|PITPNB\|LOC101903015\|TTC28 |
| 17 | 46540001 | 46630000 | 0.684781 | RIMBP2 |
| 17 | 46340001 | 46430000 | 0.691122 | STX2\|RIMBP2 |
| 17 | 68160001 | 68210000 | 0.662156 | TNIP3 |
| 17 | 63300001 | 63410000 | 0.737537 | ANKRD13A\|GIT2\|TCHP\|GLTP |
| 17 | 67860001 | 68010000 | 0.74165 | TTC28\|CHEK2 |
| 17 | 51760001 | 51810000 | 0.604424 | CCDC92\|DNAH10 |
| 17 | 70140001 | 70250000 | 0.758727 | PATZ1\|DRG1\|EIF4ENIF1\|SFI1 |
| 17 | 42280001 | 42350000 | 0.633518 | PDGFC |
| 17 | 36380001 | 36430000 | 0.604131 | FSTL5 |
| 17 | 7740001 | 7810000 | 0.688596 | DCLK2 |
| 18 | 48960001 | 49030000 | 0.665239 | LOC101902125\|IFNL3\|LRFN1 |
| 18 | 1740001 | 1830000 | 0.633821 | DDX19A\|AARS\|DDX19B\|EXOSC6 |
| 18 | 43340001 | 43430000 | 0.699304 | CEP89\|FAAP24\|RHPN2 |
| 18 | 43920001 | 43970000 | 0.597358 | PEPD |
| 18 | 1560001 | 1610000 | 0.604215 | IL34\|SF3B3 |
| 18 | 64100001 | 64150000 | 0.602459 | PEG3 |
| 19 | 24640001 | 24690000 | 0.595877 | ZZEF1 |
| 19 | 19500001 | 19550000 | 0.696736 | LYRM9 |
| 19 | 63180001 | 63310000 | 0.671099 | HELZ |
| 19 | 61920001 | 61970000 | 0.602386 | GNA13 |
| 19 | 58320001 | 58370000 | 0.60085 | SLC39A11 |
| 19 | 37960001 | 38110000 | 0.780406 | SKAP1 |
| 19 | 62900001 | 62990000 | 0.652816 | PRKCA\|CACNG5 |
| 19 | 63060001 | 63150000 | 0.776222 | CACNG4\|CACNG1\|HELZ |
| 19 | 27440001 | 27570000 | 0.692663 | DNAH2\|KDM6B\|TMEM88\|NAA38\|CYB5D1 |
| 19 | 40540001 | 40590000 | 0.620817 | CDC6\|RARA |
| 19 | 37300001 | 37370000 | 0.69472 | ZNF652\|PHOSPHO1\|ABI3\|GNGT2 |
| 19 | 6880001 | 6970000 | 0.722983 | LOC527335 |
| 19 | 20160001 | 20210000 | 0.604182 | NEK8\|TRAF4\|FAM222B |
| 19 | 9860001 | 9950000 | 0.685351 | PPM1E\|TRIM37 |
| 19 | 26600001 | 26670000 | 0.645254 | ZMYND15\|CXCL16\|MED11\|PELP1\|ARRB2 |
| 19 | 22480001 | 22530000 | 0.648418 | DOC2B\|YWHAE |
| 20 | 16820001 | 17170000 | 0.792848 | IPO11\|DIMT1\|KIF2A |
| 20 | 62180001 | 62250000 | 0.615873 | CTNND2 |
| 20 | 4660001 | 4710000 | 0.674446 | ERGIC1 |
| 20 | 320001 | 370000 | 0.600122 | PANK3 |
| 20 | 32820001 | 32890000 | 0.618892 | OXCT1 |
| 20 | 23040001 | 23110000 | 0.622296 | ANKRD55 |
| 20 | 5660001 | 5790000 | 0.822083 | CPEB4\|C20H5orf47\|NSG2 |
| 20 | 62040001 | 62150000 | 0.672588 | CTNND2 |
| 20 | 3100001 | 3190000 | 0.693106 | RANBP17\|TLX3 |
| 20 | 57500001 | 57550000 | 0.616834 | FBXL7 |
| 20 | 65780001 | 65850000 | 0.629341 | ADCY2 |
| 20 | 18760001 | 18830000 | 0.68371 | PDE4D |
| 20 | 71420001 | 71850000 | 0.801859 | LOC617224\|LOC104975299\|TPPP\|CEP72\|SLC9A3\|EXOC3\|AHRR\|PDCD6\|SDHA\|CCDC127\|LRRC14B |
| 20 | 28340001 | 28410000 | 0.644479 | PARP8 |
| 20 | 70840001 | 71010000 | 0.758957 | NDUFS6\|MRPL36\|LPCAT1 |
| 21 | 54260001 | 54670000 | 0.814224 | FSCB |
| 21 | 31520001 | 31590000 | 0.742639 | ETFA\|ISL2 |
| 21 | 33380001 | 33510000 | 0.651217 | SIN3A\|MAN2C1\|NEIL1\|COMMD4 |
| 21 | 17820001 | 17910000 | 0.652774 | AGBL1 |
| 21 | 24500001 | 24570000 | 0.681592 | SH3GL3 |
| 21 | 32620001 | 32790000 | 0.832661 | HMG20A\|LINGO1 |
| 21 | 14060001 | 14230000 | 0.84156 | CHD2 |
| 21 | 45420001 | 45650000 | 0.705459 | KIAA0391\|PSMA6\|NFKBIA |
| 21 | 30700001 | 30850000 | 0.697811 | CRABP1\|IREB2 |
| 21 | 45880001 | 45990000 | 0.728134 | RALGAPA1 |
| 21 | 32500001 | 32570000 | 0.698994 | PEAK1\|HMG20A |
| 21 | 32060001 | 32110000 | 0.627796 | RCN2 |
| 21 | 7720001 | 7810000 | 0.735439 | IGF1R |
| 21 | 42360001 | 42470000 | 0.709056 | NUBPL\|ARHGAP5 |
| 21 | 20960001 | 21030000 | 0.598187 | TICRR |
| 21 | 19500001 | 19590000 | 0.679371 | DET1\|LOC107131596 |
| 21 | 33240001 | 33350000 | 0.697982 | SNX33\|IMP3\|SNUPN\|PTPN9 |
| 21 | 33740001 | 33850000 | 0.672566 | RPP25\|COX5A\|FAM219B\|MPI\|SCAMP2\|ULK3 |
| 21 | 32360001 | 32470000 | 0.710351 | PEAK1 |
| 22 | 21220001 | 21310000 | 0.734905 | EDEM1\|ARL8B |
| 22 | 40420001 | 40490000 | 0.676294 | FHIT |
| 22 | 30240001 | 30310000 | 0.680449 | FOXP1 |
| 22 | 49160001 | 49330000 | 0.812629 | TEX264\|RAD54L2\|DCAF1 |
| 22 | 52100001 | 52290000 | 0.75431 | CSPG5\|ELP6\|SCAP\|PTPN23 |
| 22 | 19180001 | 19250000 | 0.652068 | GRM7 |
| 22 | 49680001 | 49790000 | 0.783238 | DOCK3\|MAPKAPK3\|CISH\|HEMK1 |
| 22 | 48560001 | 48690000 | 0.671872 | DNAH1\|GLYCTK\|WDR82\|PPM1M\|TWF2\|TLR9\|ALAS1 |
| 22 | 54920001 | 55010000 | 0.703406 | SLC6A11 |
| 22 | 58080001 | 58150000 | 0.71849 | TMEM43\|CHCHD4\|LOC112443442\|LOC112443506\|WNT7A |
| 22 | 49380001 | 49610000 | 0.816492 | MANF\|DOCK3 |
| 22 | 49860001 | 49950000 | 0.667234 | CACNA2D2 |
| 22 | 12320001 | 12410000 | 0.68157 | SCN11A\|WDR48 |
| 22 | 2840001 | 2970000 | 0.648243 | ZCWPW2 |
| 22 | 5240001 | 5290000 | 0.717668 | GADL1 |
| 23 | 10260001 | 10330000 | 0.624145 | C23H6orf222 |
| 23 | 8540001 | 8670000 | 0.729107 | PACSIN1\|SPDEF\|C23H6orf106 |
| 23 | 17100001 | 17170000 | 0.643948 | XPO5\|POLH\|GTPBP2\|MAD2L1BP\|RSPH9 |
| 23 | 10400001 | 10450000 | 0.652086 | KCTD20\|STK38 |
| 23 | 32760001 | 32810000 | 0.602725 | RIPOR2 |
| 23 | 39760001 | 39830000 | 0.644209 | NUP153 |
| 23 | 11460001 | 11550000 | 0.695421 | MDGA1 |
| 23 | 11040001 | 11090000 | 0.623056 | PIM1 |
| 24 | 49440001 | 49510000 | 0.655964 | ACAA2\|MYO5B |
| 24 | 42620001 | 42790000 | 0.709284 | GNAL\|CHMP1B\|MPPE1\|IMPA2\|CIDEA |
| 24 | 60880001 | 60930000 | 0.650157 | ZCCHC2 |
| 24 | 48660001 | 48750000 | 0.669762 | DYM |
| 24 | 3920001 | 3970000 | 0.60088 | ZNF407\|LOC112444151\|CNDP1 |
| 24 | 58240001 | 58290000 | 0.619072 | RAX\|CPLX4 |
| 24 | 20920001 | 20970000 | 0.603564 | MOCOS\|ELP2 |
| 24 | 19600001 | 19690000 | 0.731909 | CELF4 |
| 24 | 34120001 | 34210000 | 0.742605 | GATA6 |
| 24 | 25660001 | 25710000 | 0.618511 | DSG3 |
| 24 | 57240001 | 57290000 | 0.597048 | NEDD4L |
| 24 | 920001 | 1090000 | 0.768143 | ATP9B\|SALL3 |
| 24 | 41380001 | 41530000 | 0.661 | NDUFV2\|ANKRD12 |
| 25 | 3660001 | 3750000 | 0.779353 | CDIP1\|C25H16orf96\|UBALD1 |
| 25 | 19340001 | 19430000 | 0.73873 | LOC524391\|LOC786628 |
| 25 | 41620001 | 41670000 | 0.596462 | LOC104968516\|ZFAND2A |
| 25 | 39160001 | 39210000 | 0.633199 | RADIL\|AP5Z1 |
| 25 | 5460001 | 5510000 | 0.597178 | RBFOX1 |
| 25 | 5260001 | 5350000 | 0.772509 | RBFOX1 |
| 25 | 38180001 | 38230000 | 0.597412 | CYTH3 |
| 25 | 25580001 | 25730000 | 0.747472 | GSG1L\|XPO6 |
| 25 | 28700001 | 28750000 | 0.598509 | CALN1 |
| 26 | 33220001 | 33270000 | 0.676786 | VTI1A |
| 26 | 14600001 | 14710000 | 0.803992 | MYOF |
| 26 | 7260001 | 7310000 | 0.724934 | PRKG1 |
| 26 | 38280001 | 38430000 | 0.720428 | RAB11FIP2 |
| 26 | 30800001 | 30850000 | 0.605612 | MXI1 |
| 26 | 44000001 | 44070000 | 0.753973 | OAT |
| 26 | 10580001 | 10690000 | 0.707885 | STAMBPL1\|ACTA2\|FAS |
| 26 | 13740001 | 13790000 | 0.598635 | CPEB3\|MARCH5 |
| 26 | 9900001 | 9950000 | 0.596544 | RNLS |
| 26 | 25600001 | 25710000 | 0.697511 | SORCS3\|PAOX\|ECHS1\|FUOM\|PRAP1\|CALY\|LOC101905041\|ZNF511 |
| 26 | 8100001 | 8170000 | 0.615703 | PRKG1 |
| 27 | 37500001 | 37550000 | 0.610389 | CHRNB3\|CHRNA6\|THAP1 |
| 27 | 41660001 | 41730000 | 0.698057 | THRB |
| 27 | 19800001 | 19850000 | 0.610596 | MTMR7 |
| 27 | 37420001 | 37490000 | 0.615702 | CHRNB3 |
| 28 | 1280001 | 1410000 | 0.849833 | ACTA1 |
| 28 | 1520001 | 1570000 | 0.625294 | TAF5L |
| 28 | 1440001 | 1490000 | 0.644248 | NUP133\|ABCB10 |
| 28 | 26040001 | 26110000 | 0.690619 | COL13A1 |
| 28 | 23740001 | 23790000 | 0.631846 | CTNNA3 |
| 28 | 41640001 | 41710000 | 0.618226 | GLUD1\|SHLD2 |
| 28 | 15900001 | 15970000 | 0.618837 | ANK3 |
| 28 | 3180001 | 3230000 | 0.618461 | LOC614741 |
| 28 | 23420001 | 23470000 | 0.644037 | CTNNA3\|LRRTM3 |
| 28 | 16680001 | 16770000 | 0.669946 | RHOBTB1 |
| 29 | 32100001 | 32150000 | 0.612837 | FLI1 |
| 29 | 49120001 | 49210000 | 0.65719 | KCNQ1\|TRPM5\|TSSC4\|CD81 |
| 29 | 28560001 | 28630000 | 0.64886 | PKNOX2 |
| 29 | 39320001 | 39570000 | 0.691324 | PAG6\|PAG11\|LOC528815 |
| 29 | 47140001 | 47210000 | 0.720081 | ANO1 |
| 29 | 37340001 | 37430000 | 0.680033 | CD6\|CD5 |
| 29 | 6900001 | 6990000 | 0.681733 | GRM5 |
| 29 | 7200001 | 7270000 | 0.713887 | GRM5 |
| 29 | 30300001 | 30470000 | 0.798465 | KIRREL3 |
| 29 | 11320001 | 11490000 | 0.762138 | DLG2 |
| 29 | 24680001 | 24790000 | 0.72294 | LOC107131959\|DBX1 |
| 29 | 44320001 | 44370000 | 0.62002 | KLC2\|RAB1B\|CNIH2\|YIF1A\|TMEM151A\|CD248 |
| 29 | 2000001 | 2050000 | 0.670806 | FAT3 |
| 29 | 10920001 | 10970000 | 0.616254 | DLG2 |
| 29 | 48560001 | 48650000 | 0.653958 | CARS\|NAP1L4 |
| 29 | 26120001 | 26190000 | 0.647516 | SPTY2D1OS\|UEVLD |
| 29 | 34100001 | 34150000 | 0.613923 | OPCML |
| 29 | 30140001 | 30190000 | 0.699402 | KIRREL3 |
| 29 | 17460001 | 17510000 | 0.609403 | NARS2 |
| 29 | 16580001 | 16710000 | 0.714607 | TENM4 |
| 29 | 45180001 | 45250000 | 0.633086 | GRK2\|ANKRD13D\|SSH3 |

**Table S3**. A summary of genes from θπ ratio (*P*-value < 0.5%) between Yanbian and N'Dama cattle

| CHROM | START | END | θπ ratio | GENE |
| --- | --- | --- | --- | --- |
| 1 | 142840001 | 142910000 | 460.69 | SLC37A1 |
| 1 | 135960001 | 136010000 | 115.5263 | BFSP2 |
| 1 | 142600001 | 142670000 | 307.7569 | TFF2\|TFF1\|TMPRSS3 |
| 1 | 32900001 | 33010000 | 412.451 | CADM2 |
| 1 | 130680001 | 130730000 | 141.8474 | MRAS |
| 1 | 33200001 | 33250000 | 825.125 | CADM2 |
| 1 | 137840001 | 137890000 | 97.24283 | CPNE4 |
| 1 | 125540001 | 125630000 | 1019.035 | SLC9A9 |
| 1 | 155420001 | 155470000 | 255.8275 | SATB1 |
| 1 | 155520001 | 155570000 | 123.0791 | SATB1 |
| 1 | 99980001 | 100310000 | 878.3669 | WDR49\|SERPINI2\|ZBBX |
| 1 | 37220001 | 37270000 | 191.8999 | EPHA3 |
| 1 | 116800001 | 116870000 | 108.2694 | MED12L\|P2RY14 |
| 1 | 33960001 | 34010000 | 103.4487 | CADM2 |
| 1 | 106720001 | 106810000 | 176.0988 | PPM1L |
| 1 | 99920001 | 99970000 | 136.9497 | WDR49 |
| 1 | 115500001 | 115570000 | 399.6527 | MBNL1 |
| 1 | 107180001 | 107290000 | 259.4461 | IFT80\|C1H3orf80 |
| 1 | 106340001 | 106390000 | 104.8219 | B3GALNT1 |
| 1 | 26460001 | 26550000 | 143.5703 | ROBO1 |
| 1 | 107100001 | 107150000 | 169.0663 | IFT80 |
| 1 | 77180001 | 77230000 | 171.29 | P3H2 |
| 2 | 61680001 | 61850000 | 419.6926 | R3HDM1\|ZRANB3 |
| 2 | 103980001 | 104070000 | 113.9957 | MREG\|TMEM169\|PECR |
| 2 | 135820001 | 135870000 | 212.4668 | FBXO42 |
| 2 | 72660001 | 72710000 | 109.6735 | GLI2 |
| 2 | 6940001 | 6990000 | 98.74674 | WDR75 |
| 2 | 25280001 | 25350000 | 201.6734 | TLK1 |
| 2 | 135660001 | 135710000 | 96.43861 | CROCC\|NECAP2\|SPATA21 |
| 2 | 31000001 | 31090000 | 174.5842 | SCN2A\|SCN3A |
| 2 | 33760001 | 33870000 | 553.9772 | LOC107132260 |
| 2 | 71780001 | 71830000 | 92.549 | EPB41L5 |
| 2 | 62920001 | 63010000 | 406.3868 | MGAT5 |
| 2 | 79740001 | 79830000 | 402.5975 | MYO1B |
| 2 | 25520001 | 25590000 | 140.2512 | GORASP2\|LOC101905343 |
| 3 | 112900001 | 112990000 | 121.9156 | INPP5D\|ATG16L1 |
| 3 | 86440001 | 86550000 | 552.01 | FGGY |
| 3 | 19180001 | 19230000 | 176.0361 | TUFT1 |
| 3 | 113440001 | 113570000 | 416.0846 | MROH2A\|HJURP |
| 3 | 19060001 | 19110000 | 250.853 | CELF3\|SNX27 |
| 3 | 67400001 | 67530000 | 1215.672 | PIGK |
| 3 | 36120001 | 36170000 | 99.81211 | NTNG1 |
| 3 | 79020001 | 79070000 | 116.3601 | PDE4B |
| 4 | 40460001 | 40550000 | 254.6836 | CD36 |
| 4 | 71000001 | 71050000 | 94.71703 | OSBPL3 |
| 4 | 48940001 | 49010000 | 150.6514 | DLD\|LAMB1 |
| 4 | 116940001 | 116990000 | 206.8938 | PAXIP1\|HTR5A |
| 4 | 113820001 | 113910000 | 406.4531 | SMARCD3\|NUB1 |
| 4 | 46460001 | 46510000 | 99.867 | SRPK2 |
| 4 | 112300001 | 112350000 | 100.9094 | ZNF282\|ZNF212 |
| 4 | 52300001 | 52750000 | 1794.795 | TFEC |
| 5 | 83720001 | 83790000 | 329.6846 | SSPN |
| 5 | 12580001 | 12630000 | 177.9545 | TMTC2 |
| 5 | 108940001 | 108990000 | 157.8624 | CECR2 |
| 5 | 50820001 | 50870000 | 101.0241 | PPM1H |
| 5 | 83480001 | 83550000 | 228.5756 | ITPR2 |
| 5 | 97760001 | 97830000 | 178.5829 | BCL2L14 |
| 5 | 1320001 | 1390000 | 102.0316 | TMEM19\|RAB21 |
| 5 | 10040001 | 10090000 | 248.7358 | PTPRQ |
| 5 | 4740001 | 4790000 | 163.4525 | CAPS2 |
| 5 | 82420001 | 82490000 | 323.1904 | ARNTL2 |
| 5 | 68100001 | 68150000 | 158.8622 | CHST11 |
| 5 | 103380001 | 103450000 | 259.4834 | C1R\|C1S |
| 5 | 106400001 | 106550000 | 601.525 | TSPAN11 |
| 5 | 4680001 | 4730000 | 136.9939 | KCNC2 |
| 6 | 33060001 | 33110000 | 106.9051 | CCSER1 |
| 6 | 86940001 | 87150000 | 586.21 | GC |
| 6 | 77340001 | 77410000 | 131.1929 | ADGRL3 |
| 6 | 71900001 | 72030000 | 1531.953 | THEGL\|HOPX |
| 6 | 87420001 | 87510000 | 136.2429 | ADAMTS3 |
| 6 | 111060001 | 111130000 | 269.5367 | FGFBP1 |
| 6 | 110900001 | 111010000 | 1007.945 | BST1\|CD38 |
| 6 | 87520001 | 87670000 | 221.2303 | ADAMTS3 |
| 6 | 77240001 | 77310000 | 241.5715 | ADGRL3 |
| 6 | 87240001 | 87310000 | 146.719 | NPFFR2 |
| 6 | 98000001 | 98130000 | 195.1281 | COQ2\|HPSE |
| 6 | 100001 | 150000 | 105.0751 | APELA |
| 6 | 29720001 | 29810000 | 487.835 | BMPR1B |
| 6 | 26720001 | 26770000 | 121.7459 | STPG2 |
| 6 | 113760001 | 113830000 | 301.6072 | SORCS2 |
| 7 | 52620001 | 52670000 | 631.4963 | DIAPH1 |
| 7 | 57920001 | 57990000 | 95.98265 | PPP2R2B |
| 7 | 39700001 | 39770000 | 121.8362 | COL23A1\|CLK4 |
| 7 | 17460001 | 17510000 | 100.4119 | LOC100337081\|LOC518134 |
| 7 | 38220001 | 38270000 | 110.2942 | UNC5A |
| 7 | 53060001 | 53130000 | 147.7697 | GNPDA1 |
| 7 | 56960001 | 57070000 | 256.864 | PRELID2\|GRXCR2 |
| 7 | 52760001 | 52810000 | 369.4861 | ARAP3 |
| 7 | 56860001 | 56930000 | 161.2076 | PRELID2 |
| 7 | 6620001 | 6690000 | 347.9775 | EPS15L1\|KLF2 |
| 7 | 26560001 | 26610000 | 92.12566 | MEGF10 |
| 7 | 39120001 | 39230000 | 228.9152 | TMED9\|B4GALT7\|N4BP3\|RMND5B |
| 7 | 39060001 | 39110000 | 136.079 | LOC509184 |
| 7 | 38460001 | 38510000 | 425.0435 | UIMC1 |
| 8 | 106020001 | 106070000 | 106.6537 | ASTN2 |
| 8 | 65900001 | 66130000 | 1554.6 | CNTNAP3 |
| 8 | 43960001 | 44030000 | 184.2779 | KANK1 |
| 8 | 15440001 | 15530000 | 522.023 | LINGO2 |
| 8 | 68980001 | 69030000 | 108.611 | GFRA2 |
| 8 | 52360001 | 52410000 | 93.29697 | PCSK5 |
| 8 | 25980001 | 26030000 | 345.763 | ADAMTSL1 |
| 8 | 52700001 | 52750000 | 90.13694 | GCNT1\|PRUNE2 |
| 8 | 15560001 | 15610000 | 532.445 | LINGO2 |
| 8 | 106120001 | 106250000 | 246.7355 | ASTN2 |
| 8 | 11480001 | 11530000 | 410.2518 | NDUFB6\|TOPORS |
| 8 | 52560001 | 52630000 | 108.1562 | GCNT1 |
| 8 | 101160001 | 101230000 | 289.936 | C8H9orf84 |
| 8 | 50540001 | 50870000 | 640.665 | RORB\|TRPM6 |
| 8 | 73760001 | 73950000 | 1411.88 | DPYSL2 |
| 8 | 11560001 | 11690000 | 1564.505 | DDX58\|ACO1 |
| 8 | 110520001 | 110590000 | 237.1772 | CNTRL |
| 8 | 69140001 | 69210000 | 146.8117 | LOC104969383 |
| 9 | 23280001 | 23350000 | 101.3233 | ME1 |
| 9 | 86280001 | 86350000 | 495.372 | UST |
| 9 | 52520001 | 52630000 | 187.768 | MMS22L\|KLHL32 |
| 9 | 81520001 | 81570000 | 145.3733 | STX11 |
| 9 | 43380001 | 43650000 | 504.9073 | RTN4IP1\|CRYBG1 |
| 10 | 46140001 | 46190000 | 146.472 | HERC1 |
| 10 | 11340001 | 11390000 | 115.5943 | STYX |
| 10 | 43700001 | 43750000 | 125.4076 | NIN\|ABHD12B\|PYGL |
| 10 | 47740001 | 47790000 | 122.368 | TLN2 |
| 10 | 60980001 | 61030000 | 92.54202 | GALK2 |
| 10 | 47680001 | 47730000 | 400.21 | TLN2 |
| 10 | 61340001 | 61470000 | 385.5552 | SHC4\|EID1\|CEP152 |
| 10 | 60860001 | 60950000 | 122.934 | FAM227B\|GALK2 |
| 10 | 61260001 | 61310000 | 254.644 | SHC4 |
| 10 | 8520001 | 8570000 | 99.74273 | PDE8B |
| 11 | 94580001 | 94650000 | 298.4325 | DENND1A |
| 11 | 33440001 | 33490000 | 281.8315 | NRXN1 |
| 11 | 78060001 | 78170000 | 309.6079 | LDAH |
| 11 | 33320001 | 33390000 | 149.3372 | NRXN1 |
| 11 | 60440001 | 60590000 | 208.4975 | FAM161A\|CCT4\|COMMD1 |
| 11 | 64720001 | 64810000 | 173.931 | MEIS1 |
| 11 | 102940001 | 103010000 | 321.09 | TSC1\|GFI1B |
| 11 | 33240001 | 33310000 | 290.89 | NRXN1 |
| 11 | 67920001 | 67990000 | 196.5097 | AAK1 |
| 11 | 73200001 | 73250000 | 91.89733 | ADGRF3\|HADHB |
| 11 | 60640001 | 60690000 | 203.3771 | COMMD1 |
| 11 | 4580001 | 4670000 | 109.4634 | REV1\|AFF3 |
| 11 | 28720001 | 28770000 | 90.60927 | EPAS1 |
| 11 | 19700001 | 19750000 | 125.1245 | PRKD3 |
| 11 | 77640001 | 77770000 | 305.6896 | TDRD15 |
| 12 | 70900001 | 70950000 | 470.5621 | LOC100336232 |
| 12 | 30760001 | 30850000 | 583.0185 | UBL3 |
| 12 | 86940001 | 87010000 | 277.2762 | RASA3\|LOC112449103\|LOC100848072 |
| 12 | 76940001 | 77130000 | 568.9767 | PCCA |
| 12 | 20580001 | 20810000 | 421.289 | FAM124A\|SERPINE3\|INTS6 |
| 12 | 19160001 | 19230000 | 137.6799 | ARL11\|EBPL |
| 13 | 42060001 | 42110000 | 454.6105 | NAPB\|CSTL1\|CST11\|MGC133636 |
| 13 | 540001 | 710000 | 1300.221 | TMX4 |
| 13 | 1000001 | 1090000 | 250.0144 | PLCB1 |
| 13 | 41960001 | 42030000 | 445.7583 | NXT1\|GZF1 |
| 14 | 36340001 | 36390000 | 91.77063 | TERF1 |
| 14 | 60880001 | 60930000 | 92.21816 | RIMS2 |
| 14 | 60980001 | 61150000 | 300.3945 | DCAF13\|SLC25A32\|CTHRC1 |
| 14 | 20400001 | 20450000 | 96.94322 | SNTG1 |
| 14 | 73180001 | 73270000 | 132.876 | SLC26A7\|LRRC69 |
| 14 | 44260001 | 44310000 | 385.3177 | PAG1 |
| 14 | 70020001 | 70070000 | 96.45868 | GEM |
| 14 | 58860001 | 59010000 | 316.7966 | ZFPM2 |
| 14 | 44420001 | 44490000 | 181.0153 | FABP5 |
| 14 | 55260001 | 55330000 | 125.4655 | TRHR |
| 14 | 20520001 | 20570000 | 465.6345 | SNTG1 |
| 14 | 21080001 | 21130000 | 365.6949 | LOC104974020 |
| 14 | 36040001 | 36190000 | 493.3065 | KCNB2 |
| 15 | 28700001 | 28770000 | 244.8281 | SMIM35\|TMPRSS4 |
| 15 | 15680001 | 15730000 | 102.2921 | AMOTL1 |
| 15 | 46040001 | 46090000 | 151.9395 | LOC100125776\|OR2AG1\|LOC112441631 |
| 15 | 6900001 | 6990000 | 254.0585 | ANGPTL5 |
| 15 | 79640001 | 79770000 | 1112.81 | LOC100300446\|LOC100300488\|LOC100299725\|LOC100300575\|LOC100299764\|LOC100299808 |
| 15 | 30920001 | 30970000 | 136.2928 | ARHGEF12 |
| 15 | 43040001 | 43090000 | 98.765 | WEE1 |
| 15 | 63720001 | 63790000 | 278.1101 | CSTF3 |
| 15 | 79820001 | 79950000 | 707.61 | LOC618091\|LOC782555\|LOC781287\|OR5AR1\|LOC615810\|OR9G9\|LOC509124\|LOC100301259 |
| 15 | 81220001 | 81290000 | 1376.665 | LOC524304 |
| 15 | 58640001 | 58770000 | 349.0293 | KIF18A |
| 15 | 6540001 | 6590000 | 161.8278 | YAP1 |
| 15 | 6600001 | 6670000 | 145.4691 | YAP1\|CFAP300 |
| 16 | 71440001 | 71530000 | 201.9298 | DTL\|INTS7 |
| 16 | 59500001 | 59550000 | 190.5575 | RASAL2 |
| 16 | 22500001 | 22670000 | 1036.16 | LYPLAL1 |
| 16 | 39840001 | 39890000 | 118.0471 | DNM3 |
| 16 | 37200001 | 37310000 | 721.0111 | F5\|SELP |
| 16 | 72360001 | 72430000 | 187.7955 | KCNH1 |
| 16 | 50380001 | 50430000 | 103.5777 | PLCH2 |
| 16 | 49940001 | 50010000 | 447.6585 | PRDM16 |
| 17 | 44640001 | 44690000 | 551.93 | FBRSL1 |
| 17 | 7700001 | 7770000 | 246.2183 | DCLK2 |
| 17 | 69200001 | 69250000 | 178.0306 | HORMAD2 |
| 17 | 8380001 | 8430000 | 316.26 | IQCM |
| 17 | 36240001 | 36290000 | 131.5907 | FSTL5 |
| 18 | 16920001 | 16970000 | 92.62498 | N4BP1 |
| 19 | 8300001 | 8350000 | 99.47635 | MSI2 |
| 19 | 47900001 | 47950000 | 147.898 | TACO1\|MAP3K3 |
| 19 | 27780001 | 27830000 | 210.3843 | PER1\|VAMP2\|TMEM107 |
| 19 | 62760001 | 62970000 | 632.84 | PRKCA |
| 19 | 28080001 | 28150000 | 166.2045 | MYH10 |
| 19 | 27900001 | 27970000 | 224.6398 | RANGRF\|SLC25A35\|ARHGEF15\|ODF4\|KRBA2 |
| 19 | 62640001 | 62710000 | 162.966 | PRKCA |
| 19 | 28000001 | 28070000 | 377.4705 | NDEL1\|MYH10 |
| 19 | 26660001 | 26830000 | 403.1875 | PELP1\|ALOX15\|ALOX12E\|ALOX12\|RNASEK\|C19H17orf49\|BCL6B\|SLC16A13\|SLC16A11\|CLEC10A |
| 20 | 18560001 | 18630000 | 179.526 | DEPDC1B |
| 20 | 5820001 | 5930000 | 603.5488 | NSG2 |
| 20 | 16880001 | 16950000 | 702.9919 | IPO11 |
| 20 | 16820001 | 16870000 | 135.3963 | IPO11 |
| 21 | 47420001 | 47470000 | 94.10522 | MIPOL1 |
| 21 | 65040001 | 65110000 | 111.2224 | EVL\|DEGS2 |
| 21 | 56960001 | 57010000 | 454.485 | SLC24A4 |
| 21 | 55000001 | 55050000 | 114.2128 | TGM5\|TGM7 |
| 21 | 33680001 | 33730000 | 318.4992 | PPCDC\|SCAMP5 |
| 21 | 64840001 | 64890000 | 130.8658 | EML1 |
| 21 | 45500001 | 45550000 | 109.709 | KIAA0391\|PSMA6 |
| 21 | 28860001 | 28990000 | 133.4 | PCSK6 |
| 21 | 64940001 | 65030000 | 141.5958 | EVL |
| 21 | 28740001 | 28790000 | 170.716 | LOC104975368\|TARSL2 |
| 21 | 30760001 | 30830000 | 99.36022 | IREB2 |
| 22 | 49880001 | 49930000 | 706.5 | CACNA2D2 |
| 22 | 54420001 | 54490000 | 223.9717 | ATP2B2 |
| 23 | 20180001 | 20270000 | 366.9801 | ADGRF1 |
| 23 | 18880001 | 18950000 | 138.7855 | RUNX2 |
| 23 | 9040001 | 9090000 | 148.9123 | ANKS1A\|TCP11 |
| 23 | 8040001 | 8310000 | 1088.785 | GRM4 |
| 24 | 3600001 | 3650000 | 119.9624 | ZNF407 |
| 24 | 25000001 | 25050000 | 128.0491 | MEP1B |
| 24 | 41760001 | 41810000 | 584.22 | RAB31 |
| 24 | 41540001 | 41650000 | 434.0645 | TWSG1\|RALBP1 |
| 24 | 25080001 | 25150000 | 132.5904 | RNF138\|RNF125 |
| 24 | 39120001 | 39170000 | 92.66183 | EPB41L3 |
| 24 | 20560001 | 20670000 | 346.3979 | FHOD3 |
| 24 | 3720001 | 3790000 | 139.9819 | ZNF407 |
| 24 | 41680001 | 41730000 | 135.6578 | PPP4R1 |
| 24 | 41400001 | 41450000 | 470.65 | NDUFV2\|ANKRD12 |
| 24 | 23100001 | 23150000 | 384.3255 | NOL4 |
| 24 | 39020001 | 39070000 | 102.9912 | EPB41L3 |
| 24 | 30500001 | 30590000 | 117.3767 | TAF4B |
| 24 | 23260001 | 23330000 | 314.0142 | NOL4 |
| 24 | 41460001 | 41510000 | 106.0441 | ANKRD12 |
| 24 | 19620001 | 19750000 | 256.772 | CELF4 |
| 24 | 4260001 | 4350000 | 130.886 | FBXO15 |
| 25 | 20780001 | 20830000 | 284.944 | USP31 |
| 25 | 2840001 | 2890000 | 146.178 | ZNF174\|ZNF597\|NAA60\|C25H16orf90 |
| 25 | 20580001 | 20650000 | 128.6134 | HS3ST2 |
| 25 | 5320001 | 5390000 | 147.13 | RBFOX1 |
| 25 | 24800001 | 24850000 | 139.836 | KDM8\|NSMCE1 |
| 25 | 5420001 | 5510000 | 169.0536 | RBFOX1 |
| 25 | 38160001 | 38230000 | 151.356 | CYTH3 |
| 26 | 10480001 | 10530000 | 264.1879 | LIPM\|ANKRD22 |
| 26 | 38600001 | 38650000 | 126.3615 | FAM204A |
| 26 | 44440001 | 44490000 | 127.3261 | ZRANB1 |
| 26 | 2700001 | 2910000 | 303.7677 | ZWINT |
| 28 | 32040001 | 32090000 | 190.135 | LRMDA |
| 28 | 24380001 | 24430000 | 145.4866 | SIRT1 |
| 28 | 29760001 | 29810000 | 269.4375 | CAMK2G\|PLAU |
| 28 | 26060001 | 26150000 | 191.2582 | COL13A1 |
| 28 | 1460001 | 1550000 | 318.5765 | NUP133\|ABCB10\|TAF5L |
| 28 | 34960001 | 35090000 | 769.7376 | PPIF\|ZCCHC24 |
| 28 | 18060001 | 18110000 | 99.93763 | ARID5B |
| 28 | 26380001 | 26430000 | 91.0951 | TYSND1\|SAR1A\|PPA1 |
| 28 | 14880001 | 14930000 | 136.8 | PHYHIPL\|FAM13C |
| 29 | 6080001 | 6130000 | 144.6156 | NOX4 |
| 29 | 28580001 | 28670000 | 223.8311 | PKNOX2 |
| 29 | 17440001 | 17490000 | 214.6163 | NARS2 |
| 29 | 5920001 | 5990000 | 148.9885 | FOLH1B |
| 29 | 2500001 | 2590000 | 132.4083 | FAT3 |
| 29 | 7200001 | 7250000 | 867.5649 | GRM5 |
| 29 | 24700001 | 24770000 | 191.0481 | DBX1 |

**Table S4**. KEGG Pathway of candidate genes among XP-CLR, Fst and θπ ratio between Yanbian and N'Dama cattle

| Term | ID | Input number | Corrected P-value | Gene |
| --- | --- | --- | --- | --- |
| Olfactory transduction | hsa04740 | 49 | 4.83E-11 | LOC515540_LOC618124_LOC511509\|LOC526294_LOC618112_LOC615901\|OR2AP1\|GNAL\|LOC508595\|LOC788939\|LOC515619_LOC532436_LOC512399_LOC788037_OR52H1\|LOC788825\|LOC527077\|LOC532075_LOC787559\|LOC107131408\|OR2G3\|LOC788640\|LOC104973084_LOC615040\|OR10S1\|GRK2\|LOC509124\|ANO2\|LOC532486\|LOC100850308\|LOC781483_LOC781444_LOC781403\|ARRB2\|LOC522955\|LOC516467\|OR9G9_LOC100301259\|PRKG1\|LOC615810\|LOC524985\|OR4D5\|LOC787418\|LOC107132616\|LOC782910\|OR2AG1_LOC100125776\|SLC8A1\|MGC137098\|LOC100139733_LOC788524_LOC788512\|LOC531816_LOC781509_LOC787455_LOC504623\|LOC617388\|OR2C3_LOC613390\|OR2G2_LOC508626\|LOC101904323\|LOC516409\|OR2B11\|OR3A1_LOC508980\|LOC617122\|OR4C15\|LOC526713_LOC506139_LOC100847112_LOC782624_LOC529303\|OR2AG2\|LOC101904911_OR10G2\| |
| Metabolic pathways | hsa01100 | 89 | 8.09E-09 | DNMT3B\|CBS\|ALAD\|ALAS1\|LPCAT4\|ST6GAL2\|GADL1\|ADH6\|ISYNA1\|GLYCTK\|SYNJ1\|KHK\|CHSY3\|FUT6\|PMM2\|LOC790312\|PAPSS1\|LCT\|PPT2\|COX5A\|ACAA2\|CMAS\|PHOSPHO1\|IMPAD1\|ALG2\|ALDH6A1\|ALG8\|LPCAT1\|SDHA\|NDUFV2\|MAN1A1\|NDUFS6\|ACER1\|ATP6V1A\|ATP6V1E1\|CNDP1\|ADI1\|POLR2B\|DGAT1\|HMGCS2\|ACSBG2\|ACADL\|GAD1\|DPM1\|NME7\|PHGDH\|IMPA2\|GALNTL6\|AGPAT1\|FAS\|UXS1\|PIGK\|CPS1\|OAT\|CYP26B1\|PLPP2\|GALNT18\|QRSL1\|PANK3\|COX10\|ATP6V0D2\|PLCB1\|ABAT\|OLAH\|TUSC3\|ALG5\|GPI\|MTMR7\|MGAT5\|B4GALT6\|ECHS1\|PAH\|UPP2\|DUT\|EXT1\|CD38\|GLUD1\|PTS\|PIK3C3\|GALNT14\|CSAD\|POLE3\|STT3A\|FUT8\|NDUFB9\|ATP6V0E1\|PON1\|CYP4A11\|AKR1C3\| |
| Circadian entrainment | hsa04713 | 18 | 5.34E-07 | GRIN2A\|GNAS\|PRKG1\|PER3\|PRKCB\|PRKCA\|RYR1\|GRIN2B\|ADCYAP1R1\|GNAQ\|ADCY10\|ADCY2\|ADCY5\|GNGT2\|GNG10\|GNG2\|GUCY1A2\|PLCB1\| |
| Arrhythmogenic right ventricular cardiomyopathy (ARVC) | hsa05412 | 16 | 6.12E-07 | SLC8A1\|CACNG1\|ITGA5\|CACNB4\|CACNA2D2\|LAMA2\|ITGB7\|TCF7\|SGCD\|CTNNA3\|CACNG5\|SGCB\|ACTN2\|CACNG4\|ITGA4\|CTNNA1\| |
| Oxytocin signaling pathway | hsa04921 | 22 | 2.13E-06 | GNAS\|CAMK1D\|GNAQ\|MEF2C\|ADCY5\|PPP1R12A\|RYR1\|CD38\|PRKAA2\|CACNA2D2\|ADCY2\|NFATC1\|NFATC2\|GUCY1A2\|PLCB1\|CACNB4\|CACNG1\|PRKCB\|PRKCA\|MAP2K5\|CACNG5\|CACNG4\| |
| Dilated cardiomyopathy | hsa05414 | 16 | 5.47E-06 | GNAS\|SLC8A1\|CACNG1\|ITGA5\|CACNB4\|ADCY2\|CACNA2D2\|LAMA2\|ITGB7\|SGCD\|TPM4\|SGCB\|CACNG4\|CACNG5\|ADCY5\|ITGA4\| |
| Morphine addiction | hsa05032 | 16 | 6.14E-06 | GNAS\|CACNA1B\|PRKCB\|PRKCA\|GABRB2\|PDE4D\|GRK2\|PDE4B\|PDE4C\|PDE3B\|ADCY2\|ADCY5\|GNGT2\|GNG10\|GNG2\|ARRB2\| |
| Pathways in cancer | hsa05200 | 35 | 1.63E-05 | GNAS\|MTOR\|GLI3\|GNA13\|LAMA2\|LAMC1\|TRAF4\|CTBP1\|ARHGEF12\|EDNRA\|ADCY5\|FZD3\|RASGRP1\|FOXO1\|GNG2\|CTNNA3\|BMP2\|RASGRP4\|WNT7A\|LOC100336562\|CCNE1\|IGF1R\|FGF23\|TCF7\|ADCY2\|GNAQ\|GNGT2\|NFKBIA\|PLCB1\|PRKCB\|PRKCA\|FGF12\|RARA\|GNG10\|CTNNA1\| |
| Hypertrophic cardiomyopathy (HCM) | hsa05410 | 14 | 4.09E-05 | SLC8A1\|CACNG1\|ITGA5\|CACNB4\|CACNA2D2\|LAMA2\|ITGB7\|PRKAA2\|SGCD\|TPM4\|SGCB\|CACNG4\|CACNG5\|ITGA4\| |
| N-Glycan biosynthesis | hsa00510 | 11 | 4.12E-05 | ALG5\|MGAT5\|ST6GAL2\|MAN1A1\|DOLPP1\|TUSC3\|DPM1\|STT3A\|FUT8\|ALG8\|ALG2\| |
| Long-term depression | hsa04730 | 12 | 4.27E-05 | GNAS\|PRKG1\|PRKCB\|PRKCA\|GRID2\|GNA13\|RYR1\|GNAQ\|IGF1R\|PPP2CA\|GUCY1A2\|PLCB1\| |
| Gap junction | hsa04540 | 14 | 6.97E-05 | GNAS\|PRKG1\|GRM5\|PRKCB\|PRKCA\|PDGFC\|MAP2K5\|GNAQ\|ADCY2\|TUBB1\|ADCY5\|PDGFD\|GUCY1A2\|PLCB1\| |
| Endocytosis | hsa04144 | 25 | 0.000106679 | NEDD4L\|FH\|ADRB3\|CYTH3\|GRK2\|CHMP4A\|CHMP1B\|PARD3\|SH3GL3\|SH3GL2\|DRG1\|SMAP1\|IZUMO1R\|EPS15L1\|GIT2\|ARRB2\|EHD4\|IGF1R\|RAB8A\|ARFGEF1\|AGAP1\|DNAJC6\|RAB11FIP2\|SH3GLB1\|ERBB4\| |
| Vascular smooth muscle contraction | hsa04270 | 16 | 0.000112811 | GNAS\|PRKG1\|ACTA2\|PRKCB\|PRKCA\|GNA13\|CYP4A11\|GNAQ\|ADCY2\|ADCY5\|CALCRL\|ARHGEF12\|EDNRA\|PPP1R12A\|GUCY1A2\|PLCB1\| |
| Cholinergic synapse | hsa04725 | 15 | 0.000173857 | CACNA1B\|GNG2\|KCNQ3\|KCNQ1\|PRKCB\|PRKCA\|CHRM3\|ADCY2\|GNAQ\|KCNQ5\|ADCY5\|GNGT2\|GNG10\|CHRNA6\|PLCB1\| |
| PI3K-Akt signaling pathway | hsa04151 | 29 | 0.000190041 | MTOR\|PDGFC\|SGK3\|LAMA2\|FGF23\|THBS1\|ITGA5\|GNG2\|ITGA4\|FOXO3\|EPHA2\|IFNAR1\|ITGB7\|PRKAA2\|PDGFD\|YWHAE\|LAMC1\|CCND2\|CCNE1\|IGF1R\|PPP2CA\|LOC618947\|EIF4EBP1\|EIF4B\|PRKCA\|IFNAG\|FGF12\|GNGT2\|GNG10\| |
| Wnt signaling pathway | hsa04310 | 17 | 0.000220288 | CCND2\|PRKCB\|WIF1\|PRKCA\|SKP1\|LOC101906182\|TCF7\|PRICKLE1\|CTBP1\|NFATC2\|SOX17\|NFATC1\|DKK2\|FZD3\|CXXC4\|WNT7A\|PLCB1\| |
| Glutamatergic synapse | hsa04724 | 15 | 0.000224013 | GRIN2A\|GNAS\|GRM5\|PRKCB\|PRKCA\|GRM7\|GRIN2B\|GNAQ\|ADCY2\|ADCY5\|GRK2\|GNGT2\|GNG10\|GNG2\|PLCB1\| |
| Dopaminergic synapse | hsa04728 | 16 | 0.000252706 | GRIN2A\|GNAS\|CACNA1B\|PRKCB\|PRKCA\|GRIN2B\|GNAL\|GNAQ\|CALY\|ADCY5\|GNGT2\|GNG10\|GNG2\|PPP2CA\|ARRB2\|PLCB1\| |
| Synaptic vesicle cycle | hsa04721 | 11 | 0.000265106 | CACNA1B\|SYT1\|STX2\|STX3\|ATP6V0E1\|RIMS1\|ATP6V0D2\|CPLX4\|ATP6V1A\|RAB3A\|ATP6V1E1\| |
| Calcium signaling pathway | hsa04020 | 19 | 0.000326836 | GRIN2A\|SLC8A1\|CACNA1B\|ADRB3\|PHKB\|PRKCB\|PRKCA\|CHRM3\|CD38\|GNAL\|RYR1\|GNAQ\|ADCY2\|VDAC1\|ERBB4\|EDNRA\|GNAS\|GRM5\|PLCB1\| |
| Regulation of actin cytoskeleton | hsa04810 | 21 | 0.000369699 | ARHGEF12\|ITGA5\|PDGFC\|SCIN\|GNA13\|CHRM3\|PPP1R12A\|ITGB7\|SSH3\|FGF23\|IQGAP3\|VAV3\|VAV1\|PDGFD\|ACTN2\|PIP4K2A\|FGF12\|ITGA4\|CYFIP1\|DIAPH3\|DIAPH1\| |
| cAMP signaling pathway | hsa04024 | 20 | 0.000381553 | GRIN2A\|GNAS\|GLI3\|LOC527744\|GRIN2B\|ADCY2\|PDE4D\|PDE4B\|VAV3\|PDE3B\|PDE4C\|ADCYAP1R1\|HTR1B\|EDNRA\|VAV1\|ADCY5\|NFATC1\|PPP1R12A\|ADCY10\|NFKBIA\| |
| cGMP-PKG signaling pathway | hsa04022 | 18 | 0.00039131 | SLC8A1\|PRKG1\|ADRB3\|GNA13\|MEF2C\|PDE3B\|IRS2\|ADCY2\|MEF2D\|NFATC1\|NFATC2\|EDNRA\|VDAC1\|ADCY5\|PPP1R12A\|GUCY1A2\|GNAQ\|PLCB1\| |
| Focal adhesion | hsa04510 | 20 | 0.000476185 | ILK\|LAMC1\|CCND2\|PRKCB\|ITGA5\|PDGFC\|DIAPH1\|IGF1R\|PPP1R12A\|LAMA2\|ITGB7\|THBS1\|PARVG\|VAV3\|VAV1\|PDGFD\|ACTN2\|ARHGAP5\|ITGA4\|PRKCA\| |
| Serotonergic synapse | hsa04726 | 14 | 0.000596592 | GNAS\|CACNA1B\|KCNN2\|PRKCB\|PRKCA\|GABRB2\|GNAQ\|LOC100847677\|ADCY5\|HTR1B\|GNGT2\|GNG10\|GNG2\|PLCB1\| |
| Axon guidance | hsa04360 | 18 | 0.000669232 | SLIT2\|NTNG1\|EPHA5\|DPYSL5\|EPHA2\|PRKCA\|UNC5C\|SSH3\|ILK\|SRGAP2\|ROBO2\|TRPC3\|NFATC2\|ARHGEF12\|ROBO1\|SEMA3A\|ABLIM3\|BMPR2\| |
| Rap1 signaling pathway | hsa04015 | 20 | 0.000729862 | GRIN2A\|GNAS\|EPHA2\|PRKCB\|PDGFC\|IGF1R\|PRKCA\|GRIN2B\|THBS1\|GNAQ\|FGF23\|ADCY2\|PARD3\|PDGFD\|MAGI3\|FGF12\|ADCY5\|MAGI2\|SKAP1\|PLCB1\| |
| Retrograde endocannabinoid signaling | hsa04723 | 13 | 0.000781754 | CACNA1B\|GRM5\|PRKCB\|PRKCA\|GABRB2\|GNAQ\|ADCY2\|ADCY5\|RIMS1\|GNGT2\|GNG10\|GNG2\|PLCB1\| |
| Gastric acid secretion | hsa04971 | 11 | 0.000826291 | GNAS\|KCNQ1\|PRKCB\|PRKCA\|CHRM3\|SLC26A7\|GNAQ\|ADCY2\|ADCY5\|KCNE2\|PLCB1\| |
| GABAergic synapse | hsa04727 | 12 | 0.000859148 | CACNA1B\|PRKCB\|PRKCA\|GABRB2\|ABAT\|GAD1\|ADCY2\|ADCY5\|GNGT2\|GNG10\|GNG2\|SLC6A11\| |
| Salivary secretion | hsa04970 | 12 | 0.000933071 | GNAS\|PRKG1\|ADRB3\|PRKCB\|PRKCA\|CHRM3\|CD38\|GNAQ\|ADCY2\|ADCY5\|GUCY1A2\|PLCB1\| |
| MAPK signaling pathway | hsa04010 | 22 | 0.001115161 | MAPKAPK2\|CDC25B\|FGF23\|MEF2C\|RASGRP1\|NTF3\|RASGRP4\|TAB2\|ARRB2\|CACNA1B\|CACNA2D2\|MAPKAPK3\|NFATC1\|CACNB4\|CACNG1\|PRKCB\|PRKCA\|NTRK2\|MAP2K5\|CACNG5\|CACNG4\|FGF12\| |
| Tight junction | hsa04530 | 15 | 0.001334806 | PRKCB\|PRKCA\|MAGI3\|EXOC4\|SPTAN1\|ACTN2\|CTNNA3\|PARD3\|CTNNA1\|EXOC3\|CLDN17\|AMOTL1\|PPP2CA\|MAGI2\|ASH1L\| |
| Aldosterone synthesis and secretion | hsa04925 | 11 | 0.001539041 | GNAS\|FH\|CPS1\|PRKCB\|PRKCA\|SCARB1\|GNAQ\|ADCY2\|CAMK1D\|ADCY5\|PLCB1\| |
| Pancreatic secretion | hsa04972 | 12 | 0.001630766 | GNAS\|SCTR\|PRKCB\|PRKCA\|CHRM3\|CD38\|RAB8A\|GNAQ\|ADCY2\|ADCY5\|KCNQ1\|PLCB1\| |
| Phospholipase D signaling pathway | hsa04072 | 15 | 0.001796534 | GNAS\|MTOR\|GRM5\|MS4A2\|PRKCA\|GNA13\|GRM7\|AGPAT1\|CYTH3\|ADCY2\|ADCY5\|PDGFD\|PLPP2\|PDGFC\|PLCB1\| |
| TGF-beta signaling pathway | hsa04350 | 11 | 0.001966068 | SMAD9\|SMAD5\|DCN\|SKP1\|THBS1\|INHBA\|BMP5\|BMP2\|ACVR1\|PPP2CA\|BMPR2\| |
| Transcriptional misregulation in cancer | hsa05202 | 17 | 0.002073555 | FOXO1\|MEIS1\|CCND2\|MLLT1\|TLX3\|PBX3\|IGF1R\|ITGB7\|EYA1\|FLI1\|MEF2C\|MLLT3\|RUNX2\|RARA\|FUT8\|SIN3A\|PROM1\| |
| Insulin secretion | hsa04911 | 11 | 0.002135037 | GNAS\|KCNN2\|PRKCB\|PRKCA\|CHRM3\|ADCY2\|GNAQ\|ADCYAP1R1\|ADCY5\|RAB3A\|PLCB1\| |
| Adrenergic signaling in cardiomyocytes | hsa04261 | 15 | 0.002430321 | GNAS\|SLC8A1\|CACNG1\|KCNQ1\|PRKCA\|CACNA2D2\|CACNB4\|GNAQ\|ADCY2\|TPM4\|CACNG4\|CACNG5\|ADCY5\|PPP2CA\|PLCB1\| |
| Proteoglycans in cancer | hsa05205 | 18 | 0.003020761 | LUM\|FZD3\|DCN\|PRKCB\|PRKCA\|IGF1R\|EIF4B\|THBS1\|PTPN6\|ANK3\|ITGA5\|ANK2\|ARHGEF12\|ERBB4\|FRS2\|PPP1R12A\|MTOR\|WNT7A\| |
| Ubiquitin mediated proteolysis | hsa04120 | 14 | 0.00314299 | NEDD4L\|DET1\|UBE2B\|SKP1\|LOC101906182\|CDC16\|TRIM32\|RHOBTB1\|UBE2E3\|TRIM37\|HERC2\|LOC529930\|PRKN\|KLHL9\| |
| Renin secretion | hsa04924 | 9 | 0.003870707 | GNAS\|ADRB3\|PDE3B\|ADCYAP1R1\|ADCY5\|GNAQ\|EDNRA\|GUCY1A2\|PLCB1\| |
| Huntington's disease | hsa05016 | 17 | 0.003912585 | NDUFB9\|COX7A1\|POLR2B\|NDUFV2\|GRM5\|GRIN2B\|NDUFS6\|DNAH1\|GNAQ\|DNAH10\|DNAH6\|VDAC1\|COX5A\|SIN3A\|PLCB1\|SDHA\|DNAH2\| |
| Vibrio cholerae infection | hsa05110 | 8 | 0.003989635 | GNAS\|KCNQ1\|PRKCA\|ATP6V0E1\|ATP6V0D2\|ATP6V1A\|SEC61B\|ATP6V1E1\| |
| Purine metabolism | hsa00230 | 16 | 0.004043661 | PAPSS1\|POLR2B\|PNPT1\|NME7\|FHIT\|PDE4D\|PDE4B\|ADCY2\|PDE3B\|ADCY10\|POLE3\|ADCY5\|PDE4C\|GMPR2\|LOC790312\|GUCY1A2\| |
| Cell adhesion molecules (CAMs) | hsa04514 | 14 | 0.005076781 | GLG1\|CD274\|NCAM1\|NTNG1\|NRXN1\|ITGB7\|NFASC\|CADM3\|NLGN1\|NEGR1\|CD6\|ITGA4\|CLDN17\|CNTNAP2\| |
| Fat digestion and absorption | hsa04975 | 7 | 0.005219088 | APOB\|CD36\|SCARB1\|AGPAT1\|PLPP2\|DGAT1\|CLPS\| |
| Neuroactive ligand-receptor interaction | hsa04080 | 21 | 0.0058916 | CHRNB3\|GRIN2A\|THRB\|ADRB3\|SCTR\|GRM7\|GABRB2\|CHRM3\|GRIN2B\|CALCR\|P2RY6\|GRID2\|ADCYAP1R1\|HTR1B\|CALCRL\|EDNRA\|PARD3\|CHRNA9\|CHRNA6\|GPR83\|GRM5\| |
| beta-Alanine metabolism | hsa00410 | 6 | 0.006492305 | ALDH6A1\|ABAT\|GADL1\|GAD1\|CNDP1\|ECHS1\| |
| Chemokine signaling pathway | hsa04062 | 16 | 0.006654396 | FOXO3\|PRKCB\|GRK2\|CXCL16\|VAV1\|VAV3\|ADCY2\|PARD3\|ARRB2\|ADCY5\|ELMO1\|GNGT2\|GNG10\|GNG2\|NFKBIA\|PLCB1\| |
| Bile secretion | hsa04976 | 9 | 0.006770621 | SLC10A1\|FH\|KCNN2\|SLC9A3\|SCTR\|SCARB1\|ADCY2\|ADCY5\|GNAS\| |
| Inositol phosphate metabolism | hsa00562 | 9 | 0.006770621 | ALDH6A1\|SYNJ1\|IMPA2\|PIK3C3\|MTMR7\|PIP4K2A\|IMPAD1\|ISYNA1\|PLCB1\| |
| RNA transport | hsa03013 | 15 | 0.007630796 | SNUPN\|EIF4EBP1\|PABPC4L\|EIF4B\|SUMO1\|CYFIP1\|RPP30\|RPP38\|UPF3A\|NUP153\|XPO5\|EIF5B\|RPP25\|NUP133\|UPF2\| |
| Protein digestion and absorption | hsa04974 | 10 | 0.008774012 | SLC8A1\|DPP4\|COL11A1\|KCNQ1\|COL13A1\|SLC9A3\|COL17A1\|PAG11_PAG6\|COL5A3\|LOC528815\| |
| Cell cycle | hsa04110 | 12 | 0.009400895 | CDC25B\|CCND2\|CDC6\|PKMYT1\|CCNE1\|YWHAE\|SKP1\|CDC16\|CHEK2\|CDC14A\|WEE2\|WEE1\| |
| AMPK signaling pathway | hsa04152 | 12 | 0.009886175 | FOXO3\|FOXO1\|MTOR\|CD36\|EIF4EBP1\|IGF1R\|RAB8A\|RAB14\|IRS2\|FAS\|PRKAA2\|PPP2CA\| |
| Fatty acid metabolism | hsa01212 | 7 | 0.010233757 | ACSBG2\|ACADL\|PPT2\|PECR\|FAS\|ACAA2\|ECHS1\| |
| Cardiac muscle contraction | hsa04260 | 9 | 0.011060705 | SLC8A1\|CACNG1\|CACNB4\|CACNA2D2\|COX5A\|TPM4\|COX7A1\|CACNG4\|CACNG5\| |
| Longevity regulating pathway | hsa04211 | 10 | 0.011174449 | FOXO3\|FOXO1\|MTOR\|ULK3\|EIF4EBP1\|IGF1R\|PRKAA2\|IRS2\|ADCY2\|ADCY5\| |
| Longevity regulating pathway - multiple species | hsa04213 | 8 | 0.011862324 | FOXO3\|FOXO1\|MTOR\|IGF1R\|PRKAA2\|IRS2\|ADCY2\|ADCY5\| |
| Ovarian steroidogenesis | hsa04913 | 7 | 0.012166277 | GNAS\|FH\|SCARB1\|IGF1R\|ADCY2\|ADCY5\|AKR1C3\| |
| Ras signaling pathway | hsa04014 | 17 | 0.015604384 | GRIN2A\|EPHA2\|PRKCB\|PDGFC\|IGF1R\|PRKCA\|GRIN2B\|FGF23\|RASGRP1\|PDGFD\|GNGT2\|FGF12\|RASGRP4\|GNG10\|GNG2\|KSR1\|RASA3\| |
| Melanogenesis | hsa04916 | 10 | 0.015768652 | GNAS\|PRKCB\|PRKCA\|GNAQ\|TCF7\|ADCY2\|ADCY5\|FZD3\|WNT7A\|PLCB1\| |
| Hippo signaling pathway | hsa04390 | 13 | 0.016675581 | CCND2\|TP53BP2\|BMP5\|PPP2CA\|TCF7\|CTNNA3\|PARD3\|CTNNA1\|BMP2\|FZD3\|YWHAE\|WNT7A\|BMPR2\| |
| mTOR signaling pathway | hsa04150 | 13 | 0.016675581 | MTOR\|ULK3\|PRKCB\|EIF4EBP1\|IGF1R\|EIF4B\|PRKCA\|PRKAA2\|SLC38A9\|ATP6V1A\|FZD3\|ATP6V1E1\|WNT7A\| |
| Phagosome | hsa04145 | 13 | 0.01736304 | PIK3C3\|DYNC2H1\|CD36\|SCARB1\|ATP6V0E1\|ATP6V1A\|THBS1\|ATP6V0D2\|ITGA5\|TUBB1\|MARCO\|SEC61B\|ATP6V1E1\| |
| Butanoate metabolism | hsa00650 | 5 | 0.018036612 | OXCT1\|GAD1\|ECHS1\|HMGCS2\|ABAT\| |
| Regulation of lipolysis in adipocytes | hsa04923 | 7 | 0.019443039 | GNAS\|PRKG1\|ADRB3\|PDE3B\|IRS2\|ADCY2\|ADCY5\| |
| Platelet activation | hsa04611 | 11 | 0.019812544 | GNAS\|PRKG1\|GNA13\|GNAQ\|ADCY2\|ADCY5\|ARHGEF12\|PPP1R12A\|GUCY1A2\|RASGRP1\|PLCB1\| |
| Oocyte meiosis | hsa04114 | 11 | 0.020752425 | PPP2CA\|CPEB3\|PKMYT1\|CCNE1\|IGF1R\|SKP1\|CDC16\|CPEB4\|ADCY2\|ADCY5\|YWHAE\| |
| B cell receptor signaling pathway | hsa04662 | 8 | 0.021512734 | CD81\|PRKCB\|PTPN6\|VAV3\|NFATC1\|NFATC2\|VAV1\|NFKBIA\| |
| Fatty acid degradation | hsa00071 | 6 | 0.023360514 | ACSBG2\|ACADL\|CYP4A11\|ADH6\|ACAA2\|ECHS1\| |
| Hedgehog signaling pathway | hsa04340 | 6 | 0.029193493 | GLI3\|CCND2\|SPOPL\|GRK2\|CSNK1G3\|ARRB2\| |
| Endocrine and other factor-regulated calcium reabsorption | hsa04961 | 6 | 0.029193493 | GNAS\|SLC8A1\|PRKCB\|PRKCA\|GNAQ\|PLCB1\| |
| Sulfur metabolism | hsa00920 | 3 | 0.029222209 | PAPSS1\|SQOR\|IMPAD1\| |
| Valine, leucine and isoleucine degradation | hsa00280 | 6 | 0.031234286 | ALDH6A1\|OXCT1\|ACAA2\|ABAT\|HMGCS2\|ECHS1\| |
| Oxidative phosphorylation | hsa00190 | 11 | 0.031234286 | NDUFB9\|NDUFV2\|ATP6V0E1\|COX10\|NDUFS6\|COX5A\|ATP6V0D2\|COX7A1\|ATP6V1A\|SDHA\|ATP6V1E1\| |
| Phosphatidylinositol signaling system | hsa04070 | 9 | 0.032951471 | PIK3C3\|SYNJ1\|IMPA2\|MTMR7\|PRKCB\|PRKCA\|PIP4K2A\|IMPAD1\|PLCB1\| |
| Progesterone-mediated oocyte maturation | hsa04914 | 9 | 0.032951471 | CDC25B\|CPEB3\|PKMYT1\|IGF1R\|CDC16\|PDE3B\|CPEB4\|ADCY2\|ADCY5\| |
| EGFR tyrosine kinase inhibitor resistance | hsa01521 | 8 | 0.033385968 | FOXO3\|MTOR\|PRKCB\|PRKCA\|IGF1R\|PDGFC\|EIF4EBP1\|PDGFD\| |
| Natural killer cell mediated cytotoxicity | hsa04650 | 11 | 0.033835672 | IFNAR1\|LOC618947\|PRKCB\|PRKCA\|LOC100849046\|VAV3\|NFATC1\|NFATC2\|VAV1\|IFNAG\|PTPN6\| |
| African trypanosomiasis | hsa05143 | 5 | 0.034684044 | PLCB1\|TLR9\|PRKCB\|GNAQ\|PRKCA\| |
| Taurine and hypotaurine metabolism | hsa00430 | 3 | 0.034684044 | GADL1\|CSAD\|GAD1\| |
| ECM-receptor interaction | hsa04512 | 8 | 0.034933559 | LAMC1\|CD47\|CD36\|LAMA2\|ITGB7\|THBS1\|ITGA5\|ITGA4\| |
| Thyroid hormone signaling pathway | hsa04919 | 10 | 0.035896012 | FOXO1\|MTOR\|PRKCB\|PRKCA\|THRB\|RCN2\|TBC1D4\|NCOA2\|SIN3A\|PLCB1\| |
| Leukocyte transendothelial migration | hsa04670 | 10 | 0.035896012 | PRKCB\|PRKCA\|ACTN2\|VAV3\|CTNNA3\|VAV1\|CTNNA1\|ARHGAP5\|CLDN17\|ITGA4\| |
| Amoebiasis | hsa05146 | 9 | 0.036016224 | GNAS\|LAMC1\|PRKCB\|PRKCA\|GNAL\|LAMA2\|GNAQ\|ACTN2\|PLCB1\| |
| Long-term potentiation | hsa04720 | 7 | 0.036316658 | GRIN2A\|GRM5\|PRKCB\|PRKCA\|GRIN2B\|GNAQ\|PLCB1\| |
| Vitamin digestion and absorption | hsa04977 | 4 | 0.042872732 | SLC19A2\|LMBRD1\|APOB\|SCARB1\| |
| p53 signaling pathway | hsa04115 | 7 | 0.042872732 | CCND2\|STEAP3\|EI24\|CCNE1\|LOC101906182\|THBS1\|CHEK2\| |
| Parkinson's disease | hsa05012 | 11 | 0.043782903 | NDUFB9\|NDUFV2\|LOC101902760\|NDUFS6\|GNAL\|COX5A\|VDAC1\|COX7A1\|ADCY5\|PRKN\|SDHA\| |
| Fatty acid biosynthesis | hsa00061 | 3 | 0.046965654 | OLAH\|FAS\|ACSBG2\| |
| Thyroid hormone synthesis | hsa04918 | 7 | 0.047319241 | GNAS\|PRKCB\|PRKCA\|GNAQ\|ADCY2\|ADCY5\|PLCB1\| |
| Prostate cancer | hsa05215 | 8 | 0.048890916 | FOXO1\|MTOR\|PDGFC\|CCNE1\|IGF1R\|TCF7\|PDGFD\|NFKBIA\| |
| Ribosome biogenesis in eukaryotes | hsa03008 | 8 | 0.048890916 | NOP10\|NAT10\|WDR75\|RPP30\|RPP38\|NOP58\|RPP25\|EMG1\| |

**Table S5**. A summary of genes from CLR (P-value < 0.5%) in Yanbian cattle

| CHROM | START | END | CLR | GENE |
| --- | --- | --- | --- | --- |
| 16 | 43060001 | 43450000 | 1293.483 | PEX14\|DFFA\|CORT\|CENPS\|PGD\|KIF1B\|UBE4B |
| 2 | 71740001 | 72130000 | 1276.661 | EPB41L5\|TMEM185B\|RALB\|INHBB |
| 21 | 32440001 | 32710000 | 1162.508 | PEAK1\|HMG20A |
| 20 | 14180001 | 14530000 | 1068.2 | ADAMTS6\|CWC27 |
| 20 | 14700001 | 14870000 | 1054.969 | CWC27\|SREK1IP1\|SHISAL2B\|RGS7BP |
| 21 | 32140001 | 32430000 | 995.5597 | PSTPIP1\|TSPAN3\|PEAK1 |
| 20 | 14560001 | 14650000 | 961.7052 | CWC27 |
| 16 | 42800001 | 43010000 | 953.3815 | CASZ1\|PEX14 |
| 7 | 73360001 | 73630000 | 935.2038 | GABRA6\|GABRA1 |
| 26 | 400001 | 550000 | 894.0685 | OR5D18\|LOC101903496 |
| 29 | 260001 | 330000 | 829.8584 | LOC104970173\|LOC789688 |
| 16 | 51000001 | 51290000 | 790.4196 | CDK11B\|MMP23\|MIB2\|FNDC10\|SSU72\|TMEM240\|ATAD3A\|VWA1\|TMEM88B\|ANKRD65\|MRPL20\|CCNL2\|AURKAIP1\|MXRA8\|DVL1\|TAS1R3\|CPTP\|INTS11\|PUSL1\|ACAP3\|SCNN1D\|LOC112441769\|UBE2J2\|C1QTNF12\|B3GALT6\|SDF4\|TNFRSF4 |
| 16 | 51320001 | 51490000 | 786.7839 | C16H1orf159\|RNF223\|LOC526769\|AGRN\|ISG15\|HES4 |
| 11 | 25560001 | 25890000 | 763.5033 | THADA\|PLEKHH2 |
| 16 | 51520001 | 51670000 | 760.9343 | NOC2L\|SAMD11\|ZBTB17\|SPEN |
| 7 | 73180001 | 73290000 | 741.2054 | GABRB2 |
| 21 | 4700001 | 4850000 | 706.5472 | GABRG3 |
| 5 | 1.08E+08 | 1.09E+08 | 705.7351 | CACNA1C |
| 16 | 43720001 | 43870000 | 699.9685 | CLSTN1\|PIK3CD\|LOC101907127 |
| 26 | 1 | 390000 | 691.3504 | LOC112441518\|LOC112444476 |
| 15 | 55800001 | 56050000 | 652.6111 | GVQW3\|EMSY\|LRRC32 |
| 13 | 64720001 | 64990000 | 639.0923 | CEP250\|LOC112449379\|C13H20orf173\|ERGIC3\|LOC526745\|SPAG4\|CPNE1\|RBM12\|NFS1\|ROMO1\|RBM39\|PHF20 |
| 6 | 94640001 | 95070000 | 627.6035 | ANTXR2\|PRDM8\|FGF5\|CFAP299 |
| 7 | 45660001 | 45830000 | 576.8559 | C7H5orf15\|VDAC1\|TCF7\|SKP1 |
| 16 | 39500001 | 39690000 | 565.342 | DNM3 |
| 24 | 47540001 | 47890000 | 556.9518 | SMAD2\|ZBTB7C |
| 14 | 56120001 | 56310000 | 539.139 | EIF3E |
| 17 | 2020001 | 2150000 | 532.276 | NPY2R |
| 27 | 33280001 | 33650000 | 524.2519 | ASH2L\|STAR\|LSM1\|BAG4\|LOC112444613\|DDHD2\|PLPP5\|NSD3\|LOC104976117\|LETM2\|LOC107131134\|FGFR1 |
| 14 | 600001 | 690000 | 521.2489 | DGAT1\|HSF1\|BOP1\|SCX\|MROH1 |
| 6 | 45760001 | 45910000 | 514.6173 | RBPJ |
| 4 | 94160001 | 94430000 | 512.4315 | CEP41\|MEST\|COPG2\|MARK4 |
| 16 | 42560001 | 42670000 | 502.5244 | SRM\|MASP2\|TARDBP |
| 6 | 94540001 | 94630000 | 499.7602 | ANTXR2 |
| 14 | 55880001 | 56090000 | 493.4137 | EMC2 |
| 15 | 58720001 | 58970000 | 473.6435 | KIF18A\|METTL15 |
| 2 | 20880001 | 21150000 | 472.4364 | LNPK |
| 8 | 69200001 | 69490000 | 459.0412 | DOK2\|XPO7\|NPM2\|FGF17\|DMTN\|FAM160B2\|NUDT18\|HR\|REEP4\|LGI3\|SFTPC\|BMP1 |
| 8 | 79040001 | 79270000 | 454.959 | AGTPBP1\|NAA35\|GOLM1 |
| 7 | 73000001 | 73130000 | 445.1672 | GABRB2 |
| 10 | 29780001 | 30010000 | 443.4413 | FMN1\|GREM1\|SCG5 |
| 12 | 25920001 | 26230000 | 440.3456 | NBEA\|MAB21L1 |
| 2 | 1.2E+08 | 1.2E+08 | 439.7821 | DIS3L2 |
| 19 | 34340001 | 34470000 | 439.5119 | SHMT1\|SMCR8\|TOP3A\|MIEF2\|FLII\|LLGL1\|ALKBH5\|MYO15A |
| 26 | 580001 | 730000 | 436.4748 | UBE2D1 |
| 14 | 21700001 | 22030000 | 430.8867 | OPRK1\|ATP6V1H\|RGS20\|TCEA1\|LYPLA1\|MRPL15 |
| 2 | 90840001 | 91010000 | 426.9358 | BMPR2 |
| 24 | 49600001 | 49770000 | 420.5074 | MYO5B |
| 16 | 50860001 | 50970000 | 412.659 | GNB1\|NADK\|SLC35E2 |
| 5 | 1.08E+08 | 1.08E+08 | 406.6747 | CACNA2D4\|DCP1B\|CACNA1C |
| 19 | 34180001 | 34330000 | 404.1073 | GRAP\|SLC5A10\|FAM83G\|PRPSAP2\|SHMT1 |
| 22 | 48500001 | 48870000 | 401.3729 | DNAH1\|GLYCTK\|WDR82\|PPM1M\|TWF2\|TLR9\|ALAS1\|POC1A\|DUSP7 |
| 21 | 57660001 | 57810000 | 395.3157 | ITPK1 |
| 20 | 4700001 | 4890000 | 389.5626 | ERGIC1\|RPL26L1\|ATP6V0E1\|CREBRF\|BNIP1 |
| 17 | 35140001 | 35490000 | 389.2252 | IL2\|ADAD1\|KIAA1109 |
| 4 | 93900001 | 94030000 | 389.02 | KLHDC10\|TMEM209\|SSMEM1\|LOC101903427 |
| 2 | 91060001 | 91230000 | 371.8778 | FAM117B\|ICA1L\|WDR12 |
| 16 | 38900001 | 39070000 | 368.3288 | FMO4\|PRRC2C |
| 1 | 1.16E+08 | 1.16E+08 | 367.0499 | AADAC\|LOC782258 |
| 10 | 54800001 | 55010000 | 350.5759 | PYGO1\|DNAAF4\|CCPG1\|PIGB |
| 16 | 80300001 | 80410000 | 350.1011 | PPP1R12B\|UBE2T\|LGR6\|LOC100300760 |
| 16 | 80020001 | 80190000 | 347.0824 | KDM5B\|SYT2\|PPP1R12B |
| 7 | 4620001 | 4770000 | 345.7436 | FKBP8\|ELL\|ISYNA1\|SSBP4 |
| 5 | 34400001 | 34730000 | 343.8992 | ARID2 |
| 7 | 19520001 | 19610000 | 341.4538 | SEMA6B\|LRG1\|PLIN5 |
| 29 | 48900001 | 49170000 | 338.0228 | KCNQ1\|LOC112444897\|TRPM5 |
| 1 | 1.16E+08 | 1.16E+08 | 336.9693 | LOC528748 |
| 1 | 69560001 | 69810000 | 336.2302 | SLC12A8\|ZNF148 |
| 25 | 30380001 | 30730000 | 331.6677 | AUTS2 |
| 24 | 48560001 | 48690000 | 328.508 | SMAD7 |
| 10 | 58820001 | 58950000 | 325.9929 | DMXL2\|GLDN |
| 2 | 1.2E+08 | 1.2E+08 | 325.1181 | NPPC\|DIS3L2 |
| 24 | 49440001 | 49530000 | 317.5581 | ACAA2\|MYO5B |
| 29 | 30060001 | 30190000 | 316.941 | KIRREL3 |
| 7 | 61540001 | 61650000 | 315.207 | SLC6A7\|CAMK2A\|ARSI |
| 21 | 3740001 | 3930000 | 314.368 | GABRB3 |
| 5 | 14800001 | 14930000 | 309.3827 | LRRIQ1\|ALX1 |
| 20 | 25080001 | 25150000 | 308.8214 | ARL15 |
| 20 | 57080001 | 57330000 | 308.2773 | FBXL7 |
| 16 | 71600001 | 71750000 | 307.3903 | LPGAT1 |
| 8 | 94100001 | 94430000 | 302.9885 | OR13F1\|LOC783328\|LOC789817\|LOC789815\|OR13F1\|LOC789943\|LOC789812\|LOC513099\|OR13C3\|LOC789787 |
| 9 | 19760001 | 19870000 | 296.8523 | BCKDHB |
| 11 | 99560001 | 99670000 | 295.6636 | CRAT\|PTPA\|IER5L |
| 21 | 66760001 | 66890000 | 295.5473 | PPP2R5C\|DYNC1H1 |
| 16 | 45020001 | 45090000 | 290.8515 | RERE\|SLC45A1 |
| 14 | 80960001 | 81110000 | 290.5736 | ENPP2 |
| 2 | 1.24E+08 | 1.25E+08 | 289.3411 | TMEM200B\|EPB41 |
| 21 | 4340001 | 4430000 | 283.4077 | GABRA5\|GABRG3 |
| 29 | 38640001 | 38770000 | 283.181 | LOC784867\|PAG19 |
| 2 | 6040001 | 6150000 | 278.9103 | HIBCH\|C2H2orf88 |
| 24 | 56180001 | 56310000 | 278.3368 | WDR7 |
| 13 | 49140001 | 49250000 | 278.1952 | BMP2 |
| 8 | 78300001 | 78450000 | 277.5432 | NTRK2 |
| 9 | 41560001 | 41690000 | 277.3037 | FOXO3\|AFG1L |
| 10 | 46060001 | 46190000 | 273.8324 | DAPK2\|HERC1 |
| 24 | 800001 | 970000 | 271.0189 | NFATC1\|ATP9B |
| 5 | 9280001 | 9570000 | 266.1673 | PPP1R12A |
| 13 | 22860001 | 23110000 | 264.6711 | MLLT10\|DNAJC1 |
| 9 | 49100001 | 49350000 | 262.8833 | ASCC3 |
| 15 | 54520001 | 54690000 | 262.6091 | ARRB1\|RPS3\|KLHL35\|GDPD5 |
| 8 | 98340001 | 98470000 | 262.0982 | ACTL7B\|ACTL7A |
| 20 | 71200001 | 71310000 | 259.4668 | SLC12A7\|NKD2 |
| 21 | 14020001 | 14210000 | 255.5642 | RGMA\|CHD2 |
| 7 | 47480001 | 47650000 | 253.6138 | TGFBI\|LOC518124\|SMAD5\|SMIM32 |
| 6 | 88240001 | 88390000 | 251.0821 | ANKRD17 |
| 22 | 10720001 | 10810000 | 249.5686 | DCLK3\|GOLGA4 |
| 18 | 44760001 | 44910000 | 248.9298 | KIAA0355\|GPI\|PDCD2L\|UBA2\|WTIP |
| 14 | 15500001 | 15630000 | 247.5002 | MTSS1\|NDUFB9\|TATDN1\|RNF139\|TRMT12 |
| 9 | 41480001 | 41550000 | 246.2243 | FOXO3 |
| 3 | 1.07E+08 | 1.07E+08 | 245.9673 | MACF1 |
| 9 | 73440001 | 73550000 | 244.3898 | AHI1 |
| 12 | 26340001 | 26510000 | 242.5696 | NBEA |
| 2 | 72900001 | 73010000 | 240.8374 | TFCP2L1 |
| 1 | 1.32E+08 | 1.33E+08 | 239.087 | STAG1 |
| 21 | 32720001 | 32830000 | 238.967 | LINGO1 |
| 15 | 37920001 | 38050000 | 237.9932 | PDE3B |
| 12 | 84220001 | 84390000 | 237.3004 | MYO16 |
| 4 | 94440001 | 94510000 | 237.2513 | COPG2\|TSGA13 |
| 21 | 31620001 | 31690000 | 237.099 | SCAPER |
| 22 | 7040001 | 7150000 | 235.4802 | CNOT10 |
| 9 | 98060001 | 98150000 | 234.5575 | PRKN |
| 8 | 43540001 | 43730000 | 232.5973 | DMRT3\|DMRT1 |
| 25 | 3000001 | 3110000 | 231.5342 | TRAP1\|CREBBP |
| 24 | 48760001 | 48930000 | 230.9892 | DYM |
| 24 | 56860001 | 56950000 | 228.4104 | ATP8B1 |
| 1 | 74760001 | 74890000 | 227.3665 | FGF12 |
| 20 | 71020001 | 71150000 | 225.4057 | LPCAT1\|SLC6A3\|CLPTM1L\|TERT |
| 2 | 72660001 | 72750000 | 224.0743 | GLI2 |
| 16 | 58460001 | 58570000 | 223.9866 | BRINP2 |
| 23 | 13760001 | 13910000 | 220.6545 | DAAM2\|MOCS1 |
| 14 | 22880001 | 22990000 | 219.5248 | XKR4 |
| 10 | 59060001 | 59170000 | 217.6814 | CYP19A1 |
| 15 | 6640001 | 6770000 | 217.116 | CFAP300\|CEP126 |
| 14 | 67840001 | 67930000 | 216.4592 | PTDSS1 |
| 11 | 1.02E+08 | 1.02E+08 | 215.9614 | MED27 |
| 9 | 84340001 | 84510000 | 215.7505 | STXBP5 |
| 6 | 57360001 | 57530000 | 215.231 | TBC1D1 |
| 8 | 54100001 | 54190000 | 214.5325 | CEP78 |
| 9 | 86500001 | 86690000 | 214.4941 | TAB2 |
| 6 | 60980001 | 61090000 | 213.8597 | BEND4 |
| 1 | 76280001 | 76470000 | 212.6322 | GMNC |
| 5 | 74020001 | 74130000 | 210.2891 | RBFOX2 |
| 4 | 26300001 | 26410000 | 210.1914 | SNX13 |
| 4 | 61380001 | 61490000 | 207.6415 | HERPUD2 |
| 21 | 24180001 | 24270000 | 207.2611 | ADAMTSL3 |
| 7 | 89960001 | 90110000 | 206.9606 | MBLAC2\|POLR3G\|LYSMD3 |
| 7 | 49500001 | 49650000 | 205.8716 | WNT8A\|NME5\|LOC101902951\|BRD8\|KIF20A\|CDC23 |
| 8 | 24880001 | 25070000 | 205.809 | ACER2\|RPS6\|DENND4C |
| 26 | 20220001 | 20310000 | 205.6514 | HPSE2 |
| 8 | 77200001 | 77330000 | 205.2941 | C8H9orf64\|HNRNPK\|RMI1 |
| 12 | 36620001 | 36750000 | 204.3911 | RNF17\|ATP12A |
| 1 | 1.09E+08 | 1.09E+08 | 204.1085 | RSRC1 |
| 11 | 74820001 | 74950000 | 203.2642 | ITSN2\|FAM228A\|FAM228B |
| 14 | 34880001 | 35010000 | 202.4938 | EYA1 |
| 28 | 23700001 | 23830000 | 202.3095 | CTNNA3 |
| 1 | 37100001 | 37270000 | 202.125 | EPHA3 |
| 7 | 79120001 | 79210000 | 201.8065 | LOC112447478 |
| 1 | 65160001 | 65270000 | 201.7212 | FSTL1 |
| 5 | 36620001 | 36830000 | 201.6784 | TMEM117\|TWF1\|IRAK4\|PUS7L |
| 11 | 13860001 | 13990000 | 201.5025 | TGFA |
| 7 | 80001 | 250000 | 201.3899 | LOC112447312\|LOC101907627 |
| 11 | 99480001 | 99550000 | 198.2223 | NUP188\|SH3GLB2\|MIGA2\|DOLPP1 |
| 17 | 28780001 | 28910000 | 198.2152 | SCLT1 |
| 11 | 45600001 | 45690000 | 197.6698 | ST6GAL2 |
| 15 | 83280001 | 83370000 | 197.2468 | MS4A1\|MS4A13 |
| 14 | 60640001 | 60730000 | 196.6813 | RIMS2 |
| 15 | 14320001 | 14390000 | 195.9732 | MTMR2 |
| 11 | 1.01E+08 | 1.01E+08 | 194.7929 | HMCN2 |
| 2 | 39740001 | 39830000 | 194.521 | GPD2 |
| 3 | 81540001 | 81710000 | 193.5872 | ROR1 |
| 16 | 29000001 | 29110000 | 192.3273 | SDE2\|H3F3A |
| 9 | 61020001 | 61150000 | 191.0065 | PNRC1\|RNGTT |
| 13 | 42160001 | 42270000 | 190.4096 | LOC505033\|CST3 |
| 22 | 17160001 | 17250000 | 190.3237 | SETD5\|THUMPD3 |
| 9 | 1.03E+08 | 1.03E+08 | 190.0213 | WDR27 |
| 21 | 46420001 | 46490000 | 189.9721 | MBIP |
| 8 | 98500001 | 98570000 | 189.6183 | ELP1\|FAM206A\|CTNNAL1 |
| 7 | 45420001 | 45510000 | 188.5699 | FSTL4 |
| 25 | 25820001 | 25890000 | 188.0643 | SBK1\|LAT\|SPNS1\|NFATC2IP |
| 1 | 1.43E+08 | 1.43E+08 | 187.3655 | PDE9A\|WDR4 |
| 1 | 45680001 | 45850000 | 186.9197 | IMPG2\|SENP7 |
| 10 | 5140001 | 5290000 | 186.3292 | THOC3\|CPLX2 |
| 4 | 96680001 | 96770000 | 185.4823 | CHCHD3 |
| 5 | 38460001 | 38530000 | 185.1507 | YAF2 |
| 9 | 62360001 | 62430000 | 185.1288 | RARS2\|SLC35A1 |
| 7 | 70820001 | 70910000 | 184.4089 | UBLCP1\|IL12B |
| 10 | 71780001 | 71870000 | 183.8397 | JKAMP\|CCDC175\|RTN1 |
| 23 | 16620001 | 16750000 | 183.1059 | PEX6\|PPP2R5D\|MEA1\|KLHDC3\|RRP36\|CUL7\|MRPL2\|KLC4\|PTK7 |
| 14 | 800001 | 950000 | 182.9158 | SPATC1\|PARP10\|GRINA\|PLEC\|EPPK1 |
| 5 | 30940001 | 31090000 | 182.3854 | DDX23\|CACNB3\|ADCY6\|TEX49\|CCNT1 |
| 4 | 54320001 | 54390000 | 182.1399 | FOXP2 |
| 4 | 25700001 | 25810000 | 181.9423 | AHR |
| 11 | 99720001 | 99830000 | 179.7687 | LOC101904867 |
| 21 | 21120001 | 21230000 | 179.7465 | WDR93\|MESP1\|MESP2\|ANPEP |
| 29 | 38460001 | 38550000 | 179.2843 | PAG21 |
| 8 | 78180001 | 78270000 | 177.8222 | NTRK2 |
| 24 | 52140001 | 52230000 | 177.6202 | LOC112444158 |
| 8 | 84540001 | 84630000 | 175.3851 | WNK2 |
| 29 | 24520001 | 24610000 | 175.1711 | PRMT3\|HTATIP2\|LOC518027 |
| 2 | 1.26E+08 | 1.26E+08 | 174.973 | WASF2\|GPR3\|CD164L2\|FCN3\|MAP3K6\|SYTL1\|TMEM222 |
| 3 | 77200001 | 77350000 | 174.8928 | DIRAS3 |
| 8 | 98980001 | 99050000 | 174.1901 | PTPN3 |
| 25 | 23700001 | 23810000 | 174.069 | HS3ST4 |
| 1 | 32760001 | 32830000 | 173.8315 | CADM2 |
| 26 | 48860001 | 48950000 | 173.0339 | MGMT |
| 17 | 9860001 | 9950000 | 172.8291 | NR3C2 |
| 1 | 42740001 | 42850000 | 172.4362 | OR5K1\|CLDND1\|GPR15 |
| 21 | 19080001 | 19170000 | 171.98 | NTRK3 |
| 2 | 37640001 | 37730000 | 171.2367 | PKP4 |
| 21 | 42360001 | 42430000 | 171.1728 | NUBPL |
| 18 | 48340001 | 48450000 | 171.1286 | RYR1\|MAP4K1\|EIF3K\|ACTN4 |
| 28 | 41480001 | 41570000 | 170.8167 | BMPR1A\|MMRN2 |
| 13 | 72240001 | 72330000 | 169.8885 | MYBL2\|GTSF1L |
| 19 | 33780001 | 33890000 | 169.513 | AKAP10\|ULK2\|ALDH3A1 |
| 20 | 26140001 | 26250000 | 169.1976 | ITGA1 |
| 13 | 65000001 | 65070000 | 169.1816 | PHF20 |
| 12 | 29260001 | 29350000 | 169.1188 | RXFP2 |
| 15 | 6360001 | 6450000 | 169.0902 | BIRC2\|BIRC3 |
| 7 | 61660001 | 61790000 | 169.0604 | TCOF1\|CD74\|RPS14 |
| 14 | 6360001 | 6450000 | 168.8782 | KHDRBS3 |
| 17 | 8140001 | 8230000 | 168.4779 | IQCM |
| 14 | 44160001 | 44250000 | 166.8619 | PAG1 |
| 7 | 70740001 | 70810000 | 166.8232 | RNF145 |
| 6 | 17140001 | 17230000 | 166.5474 | LEF1\|HADH |
| 17 | 67640001 | 67750000 | 166.3502 | TTC28 |
| 4 | 1.19E+08 | 1.19E+08 | 166.0219 | NCAPG2\|ESYT2 |
| 4 | 56340001 | 56470000 | 165.7651 | DOCK4 |
| 20 | 70780001 | 70870000 | 165.73 | IRX4\|NDUFS6\|MRPL36 |
| 13 | 66720001 | 66830000 | 165.4242 | CTNNBL1\|VSTM2L |
| 1 | 1.11E+08 | 1.11E+08 | 165.1952 | TIPARP |
| 19 | 46100001 | 46170000 | 164.6037 | KANSL1 |
| 1 | 1.11E+08 | 1.11E+08 | 164.2892 | KCNAB1 |
| 3 | 1.11E+08 | 1.11E+08 | 163.8479 | LOC101907642\|DLGAP3 |
| 15 | 14480001 | 14550000 | 163.3864 | CEP57\|FAM76B |
| 8 | 96660001 | 96750000 | 163.3575 | ZNF462 |
| 28 | 33360001 | 33510000 | 163.3227 | KCNMA1 |
| 2 | 31200001 | 31310000 | 163.0782 | SLC38A11 |
| 4 | 93580001 | 93710000 | 162.752 | NRF1\|UBE2H |
| 12 | 69020001 | 69090000 | 162.3957 | LOC107131398\|TGDS |
| 11 | 81020001 | 81090000 | 162.3097 | VSNL1 |
| 11 | 88560001 | 88630000 | 162.1832 | KIDINS220\|ID2 |
| 16 | 39160001 | 39270000 | 161.4733 | VAMP4\|METTL13\|DNM3 |
| 3 | 12100001 | 12190000 | 161.1381 | LOC788175\|LOC512286 |
| 5 | 57420001 | 57510000 | 161.1371 | MMP19\|LOC507581\|DNAJC14\|ORMDL2\|SARNP\|GDF11 |
| 24 | 46360001 | 46430000 | 161.0964 | LOXHD1\|ST8SIA5 |
| 4 | 54400001 | 54490000 | 160.1992 | FOXP2 |
| 2 | 9420001 | 9510000 | 159.7888 | ZSWIM2 |
| 19 | 53260001 | 53390000 | 159.0463 | RBFOX3\|ENGASE\|C1QTNF1 |
| 4 | 80660001 | 80730000 | 158.2764 | SUGCT |
| 5 | 97500001 | 97570000 | 158.2255 | LRP6 |
| 18 | 56020001 | 56090000 | 157.7129 | PRR12\|RRAS\|SCAF1\|IRF3\|BCL2L12\|PRMT1\|ADM5 |
| 4 | 19080001 | 19170000 | 157.6236 | PHF14 |
| 29 | 32600001 | 32690000 | 157.3451 | BARX2 |
| 8 | 72580001 | 72670000 | 157.2835 | DOCK5 |
| 16 | 3540001 | 3630000 | 156.7812 | SLC41A1\|PM20D1 |
| 8 | 94980001 | 95070000 | 155.702 | SLC44A1 |
| 15 | 58280001 | 58390000 | 154.3067 | BDNF |
| 4 | 91420001 | 91530000 | 153.4664 | GRM8 |
| 10 | 5320001 | 5410000 | 153.2616 | CPLX2 |
| 14 | 34000001 | 34090000 | 152.8453 | NCOA2 |
| 16 | 28540001 | 28630000 | 151.7135 | ENAH |
| 10 | 92820001 | 92890000 | 151.6059 | SEL1L |
| 20 | 42260001 | 42350000 | 150.3259 | CDH6 |
| 20 | 71600001 | 71670000 | 150.0948 | SLC9A3\|EXOC3 |
| 7 | 8340001 | 8430000 | 149.5644 | LOC782513\|LOC100299465\|LOC107131150 |
| 2 | 1680001 | 1750000 | 149.4507 | ARHGEF4 |
| 5 | 40820001 | 40910000 | 149.2143 | SLC2A13 |
| 11 | 45700001 | 45790000 | 148.9781 | UXS1 |
| 3 | 9260001 | 9350000 | 148.7173 | VANGL2\|LOC104971425\|NHLH1\|NCSTN |
| 3 | 14000001 | 14090000 | 148.4666 | INSRR\|SH2D2A\|PRCC\|HDGF |
| 11 | 20900001 | 20970000 | 148.4433 | HNRNPLL |
| 2 | 1.23E+08 | 1.23E+08 | 148.3415 | LAPTM5 |
| 14 | 1060001 | 1130000 | 148.0136 | CCDC166\|ZNF623\|LOC101903326\|TSTA3\|PYCR3\|TIGD5\|EEF1D\|NAPRT |
| 6 | 1.05E+08 | 1.05E+08 | 146.9906 | WDR1 |
| 5 | 35260001 | 35350000 | 146.9722 | DBX2 |
| 13 | 5720001 | 5810000 | 146.7473 | LOC107133022 |
| 2 | 1.21E+08 | 1.21E+08 | 146.5949 | TMEM54\|HPCA\|FNDC5\|S100PBP |
| 9 | 80740001 | 80810000 | 146.5885 | AIG1 |
| 7 | 34600001 | 34670000 | 145.3783 | TNFAIP8\|DMXL1 |
| 25 | 19820001 | 19890000 | 145.051 | VWA3A\|SDR42E2\|EEF2K |
| 3 | 1460001 | 1530000 | 144.9133 | POU2F1 |
| 3 | 14320001 | 14390000 | 144.7782 | MEF2D |

**Table S6**. A summary of genes from θπ (P-value < 0.5%) in Yanbian cattle

| CHROM | START | END | -log(θπ) | GENE |
| --- | --- | --- | --- | --- |
| 1 | 1.16E+08 | 1.16E+08 | 3.75012 | AADAC\|LOC782258 |
| 1 | 1.17E+08 | 1.17E+08 | 3.77645 | IGSF10 |
| 1 | 1.11E+08 | 1.11E+08 | 3.76972 | KCNAB1 |
| 1 | 1.58E+08 | 1.58E+08 | 3.98001 | TXLNA |
| 1 | 69600001 | 69690000 | 3.77951 | SLC12A8\|ZNF148 |
| 1 | 1.09E+08 | 1.09E+08 | 3.7021 | RSRC1 |
| 1 | 1.32E+08 | 1.33E+08 | 3.65913 | STAG1 |
| 1 | 83140001 | 83190000 | 3.65372 | ABCC5 |
| 1 | 76380001 | 76470000 | 3.86956 | GMNC |
| 1 | 65180001 | 65310000 | 3.85509 | FSTL1 |
| 1 | 1.16E+08 | 1.16E+08 | 3.75139 | LOC528748 |
| 1 | 1.58E+08 | 1.58E+08 | 4.24119 | GPX5\|GPX6\|LOC112448270\|LOC527195 |
| 1 | 8940001 | 8990000 | 3.668 | LOC526789 |
| 1 | 65700001 | 65750000 | 3.69869 | STXBP5L |
| 1 | 69720001 | 69770000 | 3.66146 | ZNF148 |
| 2 | 1.26E+08 | 1.26E+08 | 3.81362 | WASF2\|GPR3\|CD164L2\|FCN3\|MAP3K6\|SYTL1\|TMEM222 |
| 2 | 71520001 | 71590000 | 3.69081 | PTPN4 |
| 2 | 71600001 | 71730000 | 3.78226 | PTPN4 |
| 2 | 72620001 | 72710000 | 3.75753 | GLI2 |
| 2 | 1.26E+08 | 1.26E+08 | 3.73198 | FGR\|AHDC1 |
| 2 | 71780001 | 72030000 | 4.31546 | EPB41L5\|TMEM185B\|RALB |
| 2 | 6080001 | 6130000 | 3.68887 | HIBCH\|C2H2orf88 |
| 2 | 1.21E+08 | 1.21E+08 | 3.85185 | CCDC28B |
| 2 | 1.25E+08 | 1.25E+08 | 3.7205 | RCC1\|PHACTR4 |
| 2 | 1.24E+08 | 1.25E+08 | 3.71399 | TMEM200B\|EPB41 |
| 2 | 1.2E+08 | 1.2E+08 | 3.92653 | DIS3L2 |
| 2 | 20980001 | 21070000 | 3.73168 | LNPK |
| 2 | 1680001 | 1750000 | 3.74536 | ARHGEF4 |
| 2 | 1.19E+08 | 1.19E+08 | 3.67439 | PTMA\|PDE6D |
| 2 | 72920001 | 73070000 | 4.03981 | TFCP2L1 |
| 2 | 20760001 | 20810000 | 3.67414 | HOXD4\|HOXD8\|HOXD9\|HOXD10\|HOXD11 |
| 2 | 90880001 | 90930000 | 3.74441 | BMPR2 |
| 3 | 21460001 | 21510000 | 3.66328 | ITGA10\|ANKRD35\|PIAS3 |
| 3 | 12100001 | 12210000 | 3.96563 | LOC788175\|LOC512286 |
| 3 | 80460001 | 80510000 | 3.70296 | JAK1 |
| 3 | 1.19E+08 | 1.19E+08 | 3.89554 | CSF2RA |
| 3 | 1.07E+08 | 1.07E+08 | 3.6861 | RHBDL2 |
| 3 | 1.07E+08 | 1.07E+08 | 3.75818 | MACF1 |
| 3 | 54240001 | 54350000 | 3.86103 | GBP4\|LOC112445996\|LOC112445995 |
| 3 | 3120001 | 3170000 | 3.72651 | TMCO1 |
| 4 | 7740001 | 7790000 | 3.73046 | SUN3\|CLDN12 |
| 4 | 93700001 | 93930000 | 3.73352 | UBE2H\|ZC3HC1\|KLHDC10 |
| 4 | 68800001 | 68850000 | 3.65372 | EVX1\|LOC112446393\|HOXA13 |
| 4 | 54360001 | 54450000 | 3.74221 | FOXP2 |
| 4 | 68900001 | 68950000 | 3.87209 | HOXA5\|HOXA3\|HOXA4\|HOXA2\|HOXA1 |
| 4 | 94200001 | 94550000 | 4.2412 | MEST\|COPG2\|MARK4\|TSGA13\|KLF14 |
| 4 | 93600001 | 93690000 | 3.67981 | UBE2H |
| 4 | 93980001 | 94130000 | 4.02573 | TMEM209\|SSMEM1\|LOC101903427\|CPA5\|CPA1 |
| 4 | 56380001 | 56470000 | 3.8151 | DOCK4 |
| 4 | 96700001 | 96790000 | 3.84983 | CHCHD3 |
| 4 | 47960001 | 48010000 | 3.6953 | PIK3CG |
| 4 | 76760001 | 76810000 | 3.94514 | LOC112446406\|OGDH |
| 5 | 57060001 | 57110000 | 3.68941 | CS\|COQ10A\|ANKRD52\|SLC39A5\|NABP2\|RNF41 |
| 5 | 30520001 | 30570000 | 3.68637 | PRPH\|LOC100141266\|TUBA1C |
| 5 | 56680001 | 56830000 | 3.74948 | HSD17B6\|PRIM1\|NACA\|PTGES3\|ATP5F1B\|BAZ2A\|RBMS2 |
| 5 | 27600001 | 27650000 | 4.08363 | KRT81\|LOC615451\|LOC112446672\|LOC112446673 |
| 5 | 25960001 | 26090000 | 4.12703 | HOXC4\|HOXC5\|HOXC6\|HOXC8\|HOXC9\|HOXC10\|HOXC11\|HOXC12\|HOXC13 |
| 5 | 24740001 | 24810000 | 3.73168 | NR2C1\|FGD6 |
| 5 | 38460001 | 38550000 | 3.87591 | YAF2\|GXYLT1 |
| 5 | 1.13E+08 | 1.13E+08 | 3.69781 | TEF\|TOB2 |
| 5 | 56940001 | 57030000 | 3.81845 | TIMELESS\|APOF\|ApoN\|STAT2\|IL23A\|PAN2 |
| 5 | 74020001 | 74110000 | 3.74567 | RBFOX2 |
| 5 | 97520001 | 97570000 | 3.66723 | LRP6 |
| 5 | 1.09E+08 | 1.09E+08 | 3.84463 | CACNA1C |
| 5 | 14820001 | 14910000 | 3.95732 | LRRIQ1 |
| 5 | 57420001 | 57570000 | 3.90685 | MMP19\|LOC507581\|DNAJC14\|ORMDL2\|SARNP\|GDF11\|CD63\|RDH5\|BLOC1S1\|ITGA7 |
| 6 | 17160001 | 17210000 | 3.72802 | LEF1\|HADH |
| 6 | 94620001 | 94670000 | 3.672 | ANTXR2 |
| 6 | 67300001 | 67350000 | 3.6625 | FRYL |
| 6 | 60001 | 130000 | 3.99548 | APELA |
| 6 | 88220001 | 88330000 | 3.94614 | ANKRD17 |
| 6 | 94960001 | 95050000 | 3.67602 | FGF5\|CFAP299 |
| 6 | 1.13E+08 | 1.13E+08 | 4.19169 | LOC107132586 |
| 6 | 35740001 | 35790000 | 3.69445 | FAM13A |
| 6 | 94540001 | 94610000 | 3.6568 | ANTXR2 |
| 6 | 1 | 50000 | 4.05242 | LOC788476 |
| 6 | 60980001 | 61030000 | 3.74726 | BEND4 |
| 6 | 94820001 | 94870000 | 3.68887 | PRDM8 |
| 6 | 45740001 | 45910000 | 4.04924 | RBPJ |
| 7 | 68560001 | 68610000 | 4.01671 | LOC112447526 |
| 7 | 21900001 | 21950000 | 3.79164 | IRF1\|LOC107132617 |
| 7 | 43340001 | 43610000 | 3.90914 | PTBP1\|PLPPR3\|AZU1\|PRTN3\|ELANE\|CFD\|MED16\|R3HDM4\|KISS1R\|ARID3A\|WDR18\|GRIN3B\|TMEM259\|CNN2\|ABCA7\|ARHGAP45\|POLR2E\|GPX4\|SBNO2 |
| 7 | 49820001 | 49910000 | 3.74064 | EGR1\|ETF1\|HSPA9\|CTNNA1 |
| 7 | 43620001 | 43750000 | 3.74158 | SBNO2\|STK11\|CBARP\|ATP5F1D\|MIDN\|CIRBP\|C7H19orf24\|EFNA2 |
| 7 | 17360001 | 17430000 | 3.70034 | LOC100337044 |
| 7 | 39080001 | 39130000 | 3.69249 | LOC509184\|TMED9\|B4GALT7 |
| 7 | 45720001 | 45990000 | 4.32736 | TCF7\|SKP1\|PPP2CA\|CDKL3\|UBE2B\|CDKN2AIPNL |
| 7 | 16740001 | 16790000 | 3.68063 | ELAVL1\|CCL25\|FBN3 |
| 7 | 20480001 | 20550000 | 3.9096 | NFIC |
| 7 | 11520001 | 11630000 | 3.78122 | ADGRL1\|ASF1B\|PRKACA\|SAMD1\|C7H19orf67\|MISP3 |
| 7 | 49540001 | 49670000 | 3.74631 | NME5\|LOC101902951\|BRD8\|KIF20A\|CDC23 |
| 7 | 17720001 | 17810000 | 3.85834 | VAV1\|SH2D3A\|TRIP10\|GPR108\|C3 |
| 7 | 4640001 | 4770000 | 3.97625 | FKBP8\|ELL\|ISYNA1\|SSBP4 |
| 7 | 46080001 | 46190000 | 4.01915 | JADE2\|SAR1B\|SEC24A |
| 7 | 4120001 | 4170000 | 3.71105 | TMEM161A\|SLC25A42\|ARMC6 |
| 7 | 50060001 | 50170000 | 3.79944 | CTNNA1 |
| 7 | 42900001 | 42950000 | 3.80556 | LOC522560\|LOC788709\|LOC112447520\|LOC787611 |
| 7 | 38940001 | 39050000 | 3.84147 | GRK6\|PRR7\|DBN1\|PDLIM7\|DOK3\|DDX41\|FAM193B |
| 7 | 73380001 | 73590000 | 4.03302 | GABRA6\|GABRA1 |
| 7 | 1.1E+08 | 1.1E+08 | 4.20785 | WDR36 |
| 7 | 18940001 | 19030000 | 3.99773 | PTPRS |
| 7 | 19540001 | 19590000 | 3.66591 | SEMA6B |
| 7 | 70660001 | 70790000 | 3.98439 | EBF1\|RNF145 |
| 7 | 73200001 | 73270000 | 3.73137 | GABRB2 |
| 7 | 80001 | 230000 | 4.08776 | LOC112447312\|LOC101907627 |
| 7 | 47520001 | 47590000 | 3.77138 | SMAD5 |
| 8 | 39160001 | 39230000 | 3.77679 | PDCD1LG2 |
| 8 | 43640001 | 43690000 | 3.73137 | DMRT1 |
| 8 | 25000001 | 25050000 | 3.67468 | DENND4C |
| 8 | 86600001 | 86650000 | 3.65706 | AUH |
| 8 | 24900001 | 24970000 | 3.67765 | RPS6\|DENND4C |
| 8 | 54020001 | 54170000 | 4.10987 | CEP78 |
| 8 | 69240001 | 69450000 | 3.87379 | XPO7\|NPM2\|FGF17\|DMTN\|FAM160B2\|NUDT18\|HR |
| 8 | 1.12E+08 | 1.12E+08 | 3.80518 | MYT1L |
| 8 | 73220001 | 73270000 | 3.65809 | EBF2 |
| 8 | 50840001 | 50890000 | 3.96092 | TRPM6 |
| 8 | 75960001 | 76050000 | 3.74726 | RPP25L\|DCTN3\|ARID3C\|SIGMAR1\|GALT\|IL11RA\|CCL27\|LOC508933\|LOC104969408 |
| 8 | 17160001 | 17230000 | 3.70153 | IFT74 |
| 8 | 76880001 | 76930000 | 3.73598 | UBQLN1 |
| 9 | 73060001 | 73110000 | 3.67683 | HBS1L |
| 9 | 86540001 | 86590000 | 3.68117 | TAB2 |
| 9 | 72080001 | 72130000 | 3.66094 | TBPL1 |
| 9 | 41520001 | 41590000 | 3.68693 | FOXO3 |
| 9 | 19580001 | 19630000 | 3.80775 | ELOVL4 |
| 9 | 19700001 | 19770000 | 3.95886 | TTK |
| 9 | 39320001 | 39490000 | 3.9522 | RPF2\|GTF3C6\|AMD1\|CDK19 |
| 9 | 73440001 | 73530000 | 3.86538 | AHI1 |
| 9 | 1.03E+08 | 1.04E+08 | 3.68391 | WDR27\|C9H6orf120\|PHF10 |
| 9 | 41640001 | 41690000 | 3.66407 | AFG1L |
| 10 | 36180001 | 36290000 | 3.66776 | KNL1\|RAD51 |
| 10 | 59080001 | 59210000 | 4.27728 | CYP19A1 |
| 10 | 21980001 | 22070000 | 3.79305 | MRPL52\|SLC7A7\|OXA1L |
| 10 | 58660001 | 58890000 | 4.25527 | SCG3\|DMXL2 |
| 10 | 23120001 | 23170000 | 4.04611 | LOC100336282 |
| 10 | 10020001 | 10070000 | 4.08985 | DMGDH |
| 10 | 54820001 | 54930000 | 4.18643 | PYGO1\|DNAAF4 |
| 10 | 18880001 | 18970000 | 4.13702 | MYO9A |
| 10 | 87200001 | 87270000 | 3.75948 | TTLL5\|TGFB3\|IFT43 |
| 10 | 29800001 | 29950000 | 4.21156 | FMN1 |
| 10 | 71820001 | 71870000 | 3.72742 | CCDC175\|RTN1 |
| 11 | 94960001 | 95010000 | 3.65603 | DENND1A |
| 11 | 26460001 | 26510000 | 3.77038 | PPM1B\|SLC10A6 |
| 11 | 96720001 | 96770000 | 3.75818 | PBX3 |
| 11 | 13860001 | 13970000 | 3.89599 | TGFA |
| 11 | 14680001 | 14750000 | 3.79446 | SPAST |
| 11 | 25620001 | 25850000 | 4.45391 | THADA |
| 11 | 99520001 | 99650000 | 3.84943 | MIGA2\|DOLPP1\|CRAT\|PTPA\|IER5L |
| 11 | 74820001 | 74910000 | 3.71871 | ITSN2 |
| 11 | 14360001 | 14490000 | 4.1917 | SRD5A2 |
| 11 | 20900001 | 20970000 | 3.78157 | HNRNPLL |
| 11 | 1.02E+08 | 1.02E+08 | 3.68281 | MED27 |
| 12 | 87160001 | 87250000 | 3.93329 | LOC112449111 |
| 12 | 69820001 | 69910000 | 3.97 | LOC104973089 |
| 12 | 25920001 | 26190000 | 3.9522 | NBEA\|MAB21L1 |
| 12 | 86920001 | 86970000 | 3.91006 | RASA3 |
| 12 | 72200001 | 72270000 | 3.9188 | LOC112449109 |
| 12 | 29220001 | 29310000 | 3.74915 | RXFP2 |
| 12 | 21460001 | 21530000 | 4.31664 | NEK5 |
| 12 | 36640001 | 36710000 | 3.72169 | ATP12A |
| 12 | 85040001 | 85090000 | 3.68915 | COL4A2 |
| 12 | 75020001 | 75090000 | 3.8802 | IPO5 |
| 12 | 70880001 | 70930000 | 3.8475 | LOC100336232 |
| 13 | 49120001 | 49230000 | 3.84623 | BMP2 |
| 13 | 22220001 | 22270000 | 3.67147 | NEBL |
| 13 | 66720001 | 66770000 | 3.66042 | CTNNBL1 |
| 13 | 78160001 | 78210000 | 3.67816 | TMEM189\|CEBPB |
| 13 | 22720001 | 22770000 | 3.65397 | SKIDA1\|MLLT10 |
| 13 | 22900001 | 23090000 | 3.77038 | MLLT10\|DNAJC1 |
| 13 | 65920001 | 66090000 | 4.00802 | TLDC2\|SAMHD1\|RBL1\|MROH8 |
| 13 | 11780001 | 11830000 | 3.68914 | CAMK1D |
| 13 | 64820001 | 65050000 | 4.0156 | LOC526745\|SPAG4\|CPNE1\|RBM12\|NFS1\|ROMO1\|RBM39\|PHF20 |
| 13 | 69600001 | 69690000 | 3.83251 | TOP1 |
| 13 | 77900001 | 77990000 | 3.75753 | SLC9A8\|LOC112449243\|SPATA2 |
| 13 | 64260001 | 64310000 | 3.74852 | MYH7B\|TRPC4AP |
| 14 | 44160001 | 44250000 | 3.71723 | PAG1 |
| 14 | 22900001 | 22950000 | 3.69869 | XKR4 |
| 14 | 540001 | 610000 | 3.75235 | CPSF1\|ADCK5\|SLC52A2\|FBXL6\|TMEM249\|SCRT1\|DGAT1 |
| 14 | 35100001 | 35190000 | 3.90275 | EYA1 |
| 14 | 56200001 | 56250000 | 3.69249 | EIF3E |
| 14 | 21880001 | 21930000 | 3.65449 | ATP6V1H\|RGS20 |
| 14 | 15520001 | 15630000 | 3.99886 | MTSS1\|NDUFB9\|TATDN1\|RNF139\|TRMT12 |
| 14 | 34020001 | 34130000 | 3.76079 | NCOA2 |
| 14 | 82320001 | 82410000 | 3.73626 | SLC2A5 |
| 14 | 13740001 | 13830000 | 4.14251 | LOC112449613 |
| 14 | 57180001 | 57230000 | 3.65965 | ANGPT1 |
| 14 | 640001 | 750000 | 3.73629 | BOP1\|SCX\|MROH1\|LOC112449560\|HGH1\|WDR97\|MAF1\|SHARPIN\|CYC1 |
| 14 | 41780001 | 41890000 | 4.32495 | PKIA\|ZC2HC1A |
| 15 | 58880001 | 58970000 | 3.81585 | METTL15 |
| 15 | 37940001 | 38030000 | 4.02332 | PDE3B |
| 15 | 55840001 | 56050000 | 4.09691 | EMSY\|LRRC32 |
| 15 | 14320001 | 14430000 | 3.84663 | MTMR2 |
| 15 | 58760001 | 58810000 | 3.65449 | KIF18A\|METTL15 |
| 15 | 55380001 | 55450000 | 3.69333 | UVRAG |
| 15 | 84580001 | 84690000 | 3.94364 | LOC100299117\|LOC617614 |
| 15 | 84740001 | 84790000 | 3.9741 | LOC112441561 |
| 16 | 46480001 | 46610000 | 3.74694 | CAMTA1 |
| 16 | 43760001 | 43830000 | 3.7826 | CLSTN1\|PIK3CD |
| 16 | 61800001 | 61850000 | 3.76606 | XPR1 |
| 16 | 50880001 | 51270000 | 4.09619 | GNB1\|NADK\|SLC35E2\|CDK11B\|MMP23\|MIB2\|FNDC10\|SSU72\|TMEM240\|ATAD3A\|VWA1\|TMEM88B\|ANKRD65\|MRPL20\|CCNL2\|AURKAIP1\|MXRA8\|DVL1\|TAS1R3\|CPTP\|INTS11\|PUSL1\|ACAP3\|SCNN1D\|LOC112441769\|UBE2J2\|C1QTNF12\|B3GALT6 |
| 16 | 43380001 | 43550000 | 3.83444 | UBE4B\|RBP7 |
| 16 | 42340001 | 42530000 | 4.14093 | UBIAD1\|MTOR\|ANGPTL7\|EXOSC10 |
| 16 | 57000001 | 57050000 | 4.05243 | TNR |
| 16 | 29020001 | 29110000 | 3.91611 | SDE2\|H3F3A |
| 16 | 44740001 | 44790000 | 3.72349 | RERE |
| 16 | 42940001 | 43150000 | 4.00802 | CASZ1\|PEX14\|DFFA\|CORT\|CENPS |
| 16 | 51360001 | 51610000 | 4.26561 | C16H1orf159\|RNF223\|LOC526769\|AGRN\|ISG15\|HES4\|PERM1\|KLHL17\|NOC2L\|SAMD11\|ZBTB17 |
| 16 | 39540001 | 39670000 | 4.07545 | DNM3 |
| 16 | 71640001 | 71710000 | 3.70325 | LPGAT1 |
| 16 | 39780001 | 39910000 | 3.77815 | DNM3 |
| 16 | 58460001 | 58510000 | 3.65758 | BRINP2 |
| 16 | 44600001 | 44690000 | 3.93135 | RERE |
| 16 | 80980001 | 81030000 | 3.78157 | LOC100297820 |
| 16 | 420001 | 550000 | 4.15288 | OR5L1 |
| 16 | 80080001 | 80150000 | 3.79338 | SYT2\|PPP1R12B |
| 16 | 29240001 | 29290000 | 3.72893 | LIN9 |
| 16 | 44380001 | 44450000 | 4.15609 | LOC107131287\|CA6 |
| 16 | 38960001 | 39030000 | 3.72289 | PRRC2C |
| 16 | 60360001 | 60430000 | 3.6568 | FAM20B\|TOR3A |
| 16 | 44960001 | 45110000 | 3.89779 | RERE\|SLC45A1 |
| 16 | 42600001 | 42690000 | 4.30388 | TARDBP |
| 16 | 43580001 | 43710000 | 4.00744 | NMNAT1\|LZIC\|CTNNBIP1 |
| 17 | 70860001 | 70910000 | 3.74375 | IGLL1 |
| 17 | 55100001 | 55330000 | 3.88149 | PHETA1\|SH2B3\|ATXN2\|BRAP |
| 17 | 35300001 | 35430000 | 3.75236 | ADAD1\|KIAA1109 |
| 17 | 8460001 | 8510000 | 3.98002 | IQCM |
| 17 | 73120001 | 73170000 | 3.68609 | PRODH\|DGCR6L\|LOC101903252 |
| 17 | 35220001 | 35290000 | 3.7498 | IL2\|ADAD1 |
| 17 | 71120001 | 71190000 | 3.91846 | IGLL1\|LOC100847119\|LOC112442062\|LOC100297192 |
| 17 | 2020001 | 2110000 | 4.01797 | NPY2R |
| 17 | 67800001 | 67990000 | 3.98057 | TTC28\|CHEK2 |
| 17 | 54540001 | 54610000 | 3.67873 | PPP1CC |
| 17 | 56940001 | 57050000 | 3.75916 | TAOK3 |
| 18 | 62080001 | 62130000 | 3.65602 | UBE2S\|RPL28\|TMEM238\|TMEM190\|IL11\|FAM71E2\|COX6B2 |
| 18 | 44780001 | 44830000 | 3.70467 | GPI\|PDCD2L |
| 18 | 56000001 | 56070000 | 3.82827 | NOSIP\|PRRG2\|PRR12\|RRAS\|SCAF1\|IRF3\|BCL2L12 |
| 18 | 53160001 | 53210000 | 3.74002 | OPA3\|GPR4\|EML2 |
| 18 | 17020001 | 17070000 | 3.69812 | N4BP1 |
| 18 | 14680001 | 14790000 | 3.74726 | TCF25\|MC1R\|TUBB3\|DEF8\|CENPBD1\|LOC532875\|DBNDD1\|GAS8 |
| 18 | 65800001 | 65850000 | 3.69109 | LOC101903385\|LOC101903649 |
| 18 | 9300001 | 9350000 | 3.66407 | CDH13 |
| 18 | 34480001 | 34530000 | 3.71076 | TERB1\|NAE1 |
| 18 | 60200001 | 60250000 | 3.83598 | LOC112442368\|LOC101905616 |
| 18 | 25360001 | 25410000 | 3.69026 | CCL22\|CX3CL1 |
| 18 | 55640001 | 55690000 | 3.65602 | PPFIA3\|TRPM4\|HRC |
| 18 | 2320001 | 2390000 | 3.8325 | WDR59\|ZNRF1 |
| 19 | 38720001 | 38770000 | 3.66723 | TBX21\|TBKBP1 |
| 19 | 46080001 | 46170000 | 3.71634 | KANSL1 |
| 19 | 48760001 | 48850000 | 3.84464 | SMURF2 |
| 19 | 21380001 | 21430000 | 3.69897 | TMIGD1 |
| 19 | 22480001 | 22550000 | 3.836 | DOC2B\|YWHAE |
| 19 | 21860001 | 21910000 | 3.66934 | ABR\|TIMM22\|NXN |
| 19 | 34260001 | 34390000 | 3.90502 | SLC5A10\|FAM83G\|PRPSAP2\|SHMT1\|SMCR8\|TOP3A\|MIEF2\|FLII |
| 19 | 27440001 | 27590000 | 3.98493 | DNAH2\|KDM6B\|TMEM88\|NAA38\|CYB5D1\|CHD3\|RNF227 |
| 19 | 47000001 | 47070000 | 3.70439 | TLK2\|MRC2 |
| 19 | 36640001 | 36710000 | 3.70296 | DLX3\|DLX4\|LOC100196902 |
| 19 | 33780001 | 33910000 | 3.92701 | AKAP10\|ULK2\|ALDH3A1\|SLC47A2 |
| 20 | 71720001 | 71850000 | 3.90639 | AHRR\|PDCD6\|SDHA\|CCDC127\|LRRC14B |
| 20 | 14440001 | 14610000 | 3.87421 | CWC27 |
| 20 | 14300001 | 14350000 | 3.65809 | ADAMTS6 |
| 20 | 22020001 | 22070000 | 3.7265 | GPBP1 |
| 20 | 23760001 | 23810000 | 3.69137 | MTREX |
| 20 | 51200001 | 51250000 | 4.22374 | CDH12 |
| 20 | 14760001 | 14870000 | 4.25425 | SREK1IP1\|SHISAL2B\|RGS7BP |
| 20 | 23620001 | 23690000 | 3.70382 | PLPP1 |
| 20 | 25040001 | 25230000 | 3.93672 | ARL15 |
| 20 | 4740001 | 4850000 | 3.91239 | ATP6V0E1\|CREBRF\|BNIP1 |
| 21 | 32960001 | 33030000 | 4.46533 | LOC112443149\|LOC112443150 |
| 21 | 57680001 | 57770000 | 3.9672 | ITPK1 |
| 21 | 32600001 | 32670000 | 3.93869 | HMG20A |
| 21 | 32360001 | 32450000 | 4.07343 | PEAK1 |
| 21 | 4700001 | 4890000 | 4.00227 | GABRG3 |
| 21 | 32040001 | 32110000 | 3.96406 | RCN2 |
| 21 | 32140001 | 32250000 | 3.93135 | PSTPIP1\|TSPAN3\|PEAK1 |
| 21 | 32480001 | 32570000 | 4.24219 | PEAK1\|HMG20A |
| 21 | 58940001 | 59030000 | 3.85958 | SERPINA6 |
| 21 | 21160001 | 21210000 | 3.76639 | MESP1\|MESP2\|ANPEP |
| 21 | 32760001 | 32830000 | 3.93427 | LINGO1 |
| 22 | 32260001 | 32310000 | 3.66355 | FRMD4B |
| 22 | 50440001 | 50490000 | 3.69811 | CDHR4\|IP6K1\|GMPPB\|RNF123 |
| 22 | 49340001 | 49410000 | 3.69699 | DCAF1\|RBM15B\|MANF\|DOCK3 |
| 22 | 28920001 | 28970000 | 3.65835 | SHQ1 |
| 22 | 48540001 | 48630000 | 3.85064 | DNAH1\|GLYCTK\|WDR82 |
| 22 | 10740001 | 10870000 | 3.98439 | DCLK3\|GOLGA4 |
| 22 | 51580001 | 51630000 | 3.68969 | CATHL1\|LOC112443481\|CATHL4\|LOC112441458\|CATHL3 |
| 22 | 50100001 | 50150000 | 3.68554 | GNAI2\|SLC38A3 |
| 22 | 49680001 | 49730000 | 3.66329 | DOCK3\|MAPKAPK3 |
| 23 | 1 | 50000 | 4.06213 | LOC101906171\|LOC112443721 |
| 23 | 16660001 | 16710000 | 3.65602 | KLHDC3\|RRP36\|CUL7\|MRPL2\|KLC4\|PTK7 |
| 23 | 21740001 | 21790000 | 4.19524 | LOC613394\|LOC781635 |
| 23 | 16560001 | 16630000 | 4.02632 | CNPY3\|GNMT\|LOC112443696\|PEX6\|PPP2R5D |
| 23 | 26220001 | 26270000 | 3.84992 | LOC101903211\|LOC107131258 |
| 23 | 580001 | 650000 | 3.8604 | KHDRBS2 |
| 24 | 47760001 | 47850000 | 3.90229 | ZBTB7C |
| 24 | 62300001 | 62350000 | 4.01443 | LOC101906312 |
| 24 | 29940001 | 29990000 | 3.71575 | CHST9 |
| 24 | 48600001 | 48670000 | 3.7941 | SMAD7 |
| 25 | 27140001 | 27190000 | 3.65397 | STX1B\|STX4\|ZNF668\|ZNF646 |
| 25 | 3160001 | 3210000 | 3.66277 | CREBBP |
| 25 | 3300001 | 3350000 | 3.66539 | ADCY9 |
| 25 | 26180001 | 26270000 | 3.93622 | MAPK3\|GDPD3\|YPEL3\|TBX6\|PPP4C\|ALDOA\|FAM57B\|C25H16orf92\|DOC2A\|INO80E\|HIRIP3 |
| 25 | 23720001 | 23770000 | 3.75623 | HS3ST4 |
| 25 | 25620001 | 25730000 | 3.91893 | XPO6 |
| 25 | 30640001 | 30690000 | 3.7096 | AUTS2 |
| 25 | 9480001 | 9550000 | 3.85063 | TVP23A\|CIITA |
| 26 | 340001 | 730000 | 4.65321 | OR5D18\|LOC101903496\|UBE2D1 |
| 26 | 20200001 | 20330000 | 3.7211 | HPSE2 |
| 26 | 50260001 | 50310000 | 4.16597 | TCERG1L |
| 26 | 15200001 | 15250000 | 3.70669 | LOC112444532\|LGI1 |
| 26 | 260001 | 310000 | 3.93087 | LOC112444476 |
| 26 | 44500001 | 44550000 | 3.6651 | ZRANB1\|CTBP2 |
| 26 | 50360001 | 50410000 | 3.80592 | TCERG1L |
| 26 | 23120001 | 23170000 | 3.82598 | SUFU |
| 26 | 1 | 70000 | 4.65835 | LOC112441518 |
| 26 | 51980001 | 52030000 | 3.88539 | PWWP2B |
| 26 | 50180001 | 50230000 | 3.71693 | TCERG1L\|LOC528422 |
| 27 | 33300001 | 33610000 | 4.05888 | ASH2L\|STAR\|LSM1\|BAG4\|LOC112444613\|DDHD2\|PLPP5\|NSD3\|LOC104976117\|LETM2\|LOC107131134\|FGFR1 |
| 27 | 1100001 | 1150000 | 4.02512 | DLGAP2 |
| 27 | 34080001 | 34130000 | 3.68417 | PLEKHA2\|HTRA4 |
| 28 | 30720001 | 30770000 | 3.67468 | KAT6B |
| 28 | 16520001 | 16590000 | 3.76276 | CDK1 |
| 29 | 50980001 | 51030000 | 3.80014 | IFITM3\|LOC112444847 |
| 29 | 1880001 | 1930000 | 3.66644 | MTNR1B |
| 29 | 1 | 410000 | 4.34208 | LOC104970173\|LOC789688 |
| 29 | 48900001 | 49050000 | 4.09833 | KCNQ1\|LOC112444897 |

**Table S7**. KEGG Pathway of candidate genes between CLR and θπ in Yanbian cattle

| Term | ID | Input number | Corrected P-value | Gene |
| --- | --- | --- | --- | --- |
| TGF-beta signaling pathway | bta04350 | 15 | 9.07E-05 | MAPK3\|SMAD2\|TGFB3\|SMAD7\|SMURF2\|RBL1\|SKP1\|BMPR1A\|BMPR2\|SMAD5\|CREBBP\|INHBB\|BMP2\|ID2\|PPP2CA\| |
| Pathways in cancer | bta05200 | 36 | 0.000117417 | SMAD2\|TGFB3\|RALB\|PIK3CD\|DVL1\|FGF17\|GLI2\|FGFR1\|BIRC3\|BMP2\|PRKACA\|WNT8A\|JAK1\|LOC104970173\|SUFU\|ADCY9\|GNAI2\|MAPK3\|DAPK2\|BIRC2\|RAD51\|FGF12\|FGF5\|CTNNA3\|CSF2RA\|CTBP2\|CREBBP\|GNB1\|TCF7\|CTNNA1\|ADCY6\|PIK3CG\|COL4A2\|LEF1\|TGFA\|MTOR\| |
| Hippo signaling pathway | bta04390 | 19 | 0.000478358 | TCF7\|CTNNA1\|WNT8A\|TGFB3\|WTIP\|LLGL1\|CTNNA3\|DVL1\|BMPR2\|GLI2\|YWHAE\|SMAD7\|LEF1\|BMPR1A\|SMAD2\|BMP2\|ID2\|PPP1CC\|PPP2CA\| |
| Morphine addiction | bta05032 | 14 | 0.001007543 | ARRB1\|ADCY6\|PDE3B\|GABRB3\|GABRA1\|GABRA5\|ADCY9\|GRK6\|GABRA6\|GNAI2\|GABRG3\|GNB1\|PRKACA\|GABRB2\| |
| Oxytocin signaling pathway | bta04921 | 18 | 0.001731618 | MAPK3\|CACNB3\|NFATC1\|CACNA1C\|CACNA2D4\|PIK3CD\|PIK3CG\|PPP1R12B\|EEF2K\|ADCY9\|CAMK2A\|PPP1R12A\|GNAI2\|RYR1\|CAMK1D\|ADCY6\|PRKACA\|PPP1CC\| |
| GABAergic synapse | bta04727 | 13 | 0.001961049 | CACNA1C\|SLC38A3\|GABRB3\|GABRA1\|GABRA5\|ADCY9\|GABRA6\|GNAI2\|ADCY6\|GABRG3\|GNB1\|PRKACA\|GABRB2\| |
| Adrenergic signaling in cardiomyocytes | bta04261 | 17 | 0.002309665 | MAPK3\|CACNB3\|LOC104970173\|CACNA1C\|CACNA2D4\|PPP2R5C\|PIK3CG\|PIK3CD\|PPP2R5D\|ADCY9\|CAMK2A\|GNAI2\|ADCY6\|KCNQ1\|PRKACA\|PPP1CC\|PPP2CA\| |
| Ubiquitin mediated proteolysis | bta04120 | 16 | 0.003367357 | UBE2D1\|UBE2S\|UBE2H\|BIRC3\|UBE2J2\|PIAS3\|SMURF2\|UBE2B\|UBA2\|CUL7\|PRKN\|HERC1\|SKP1\|UBE4B\|CDC23\|BIRC2\| |
| Arrhythmogenic right ventricular cardiomyopathy (ARVC) | bta05412 | 11 | 0.003471924 | TCF7\|CTNNA1\|CACNB3\|CACNA1C\|CACNA2D4\|ITGA10\|ACTN4\|CTNNA3\|ITGA7\|LEF1\|ITGA1\| |
| Chemokine signaling pathway | bta04062 | 19 | 0.004197106 | MAPK3\|FGR\|ARRB1\|ADCY6\|LOC508933\|STAT2\|PIK3CD\|PIK3CG\|GRK6\|ADCY9\|PRKACA\|FOXO3\|GNAI2\|GNB1\|CX3CL1\|CCL27\|CCL25\|VAV1\|CCL22\| |
| Regulation of actin cytoskeleton | bta04810 | 20 | 0.007119986 | MAPK3\|PIK3CD\|FGF5\|WASF2\|FGF12\|PPP1R12B\|ITGA10\|ENAH\|INSRR\|PPP1R12A\|ITGA1\|FGF17\|ACTN4\|ITGA7\|VAV1\|PIK3CG\|FGFR1\|ARHGEF4\|RRAS\|PPP1CC\| |
| Retrograde endocannabinoid signaling | bta04723 | 13 | 0.007315994 | MAPK3\|CACNA1C\|GABRB3\|GABRA1\|GABRA5\|ADCY9\|GABRA6\|GNAI2\|ADCY6\|GABRG3\|GNB1\|PRKACA\|GABRB2\| |
| MAPK signaling pathway | bta04010 | 22 | 0.009544663 | NFATC1\|TGFB3\|TAB2\|FGF17\|FGFR1\|MAP4K1\|PRKACA\|DUSP7\|CACNA2D4\|NTRK2\|TAOK3\|MAPK3\|CACNB3\|BDNF\|ARRB1\|CACNA1C\|FGF12\|FGF5\|PPM1B\|MAP3K6\|RRAS\|MAPKAPK3\| |
| Wnt signaling pathway | bta04310 | 15 | 0.01092009 | NKD2\|TCF7\|WNT8A\|NFATC1\|CTNNBIP1\|LRP6\|DVL1\|CAMK2A\|LEF1\|CTBP2\|CREBBP\|SKP1\|DAAM2\|PRKACA\|VANGL2\| |
| Adherens junction | bta04520 | 10 | 0.011146886 | MAPK3\|CTNNA1\|CTNNA3\|SMAD2\|ACTN4\|FGFR1\|LEF1\|CREBBP\|TCF7\|WASF2\| |
| Melanogenesis | bta04916 | 12 | 0.01399657 | MAPK3\|TCF7\|WNT8A\|ADCY6\|GNAI2\|ADCY9\|CAMK2A\|LEF1\|DVL1\|CREBBP\|MC1R\|PRKACA\| |
| Dilated cardiomyopathy | bta05414 | 11 | 0.014190523 | CACNB3\|LOC104970173\|CACNA1C\|TGFB3\|ITGA10\|CACNA2D4\|ADCY9\|ITGA7\|ITGA1\|ADCY6\|PRKACA\| |
| cAMP signaling pathway | bta04024 | 18 | 0.015604517 | MAPK3\|BDNF\|NFATC1\|CACNA1C\|PDE3B\|PIK3CG\|PIK3CD\|ADCY9\|CAMK2A\|PRKACA\|PPP1R12A\|GNAI2\|VAV1\|CREBBP\|ADCY6\|GRIN3B\|RRAS\|PPP1CC\| |
| Oocyte meiosis | bta04114 | 13 | 0.015655466 | MAPK3\|ADCY6\|CDK1\|PPP2R5C\|PPP2R5D\|ADCY9\|CAMK2A\|SKP1\|YWHAE\|CDC23\|PRKACA\|PPP1CC\|PPP2CA\| |
| Tuberculosis | bta05152 | 17 | 0.016134175 | MAPK3\|MRC2\|JAK1\|CATHL1\|IL23A\|CIITA\|CEBPB\|TLR9\|TGFB3\|C3\|CAMK2A\|CD74\|IRAK4\|CREBBP\|HSPA9\|IL12B\|ATP6V1H\| |
| Prostate cancer | bta05215 | 11 | 0.016134175 | MAPK3\|TCF7\|SRD5A2\|CREBBP\|PIK3CD\|PIK3CG\|INSRR\|FGFR1\|LEF1\|TGFA\|MTOR\| |
| Signaling pathways regulating pluripotency of stem cells | bta04550 | 14 | 0.022627748 | MAPK3\|SMAD2\|WNT8A\|JAK1\|PIK3CG\|PIK3CD\|SMAD5\|DVL1\|BMPR2\|FGFR1\|BMPR1A\|INHBB\|BMP2\|ID2\| |
| Pancreatic cancer | bta05212 | 9 | 0.024473328 | MAPK3\|SMAD2\|JAK1\|RAD51\|TGFB3\|RALB\|PIK3CG\|PIK3CD\|TGFA\| |
| Endometrial cancer | bta05213 | 8 | 0.025025606 | MAPK3\|CTNNA1\|CTNNA3\|PIK3CG\|PIK3CD\|LEF1\|FOXO3\|TCF7\| |
| Chagas disease (American trypanosomiasis) | bta05142 | 12 | 0.027874009 | MAPK3\|SMAD2\|IL2\|TGFB3\|PIK3CD\|TLR9\|PIK3CG\|C3\|IRAK4\|GNAI2\|IL12B\|PPP2CA\| |
| HTLV-I infection | bta05166 | 21 | 0.028621565 | SMAD2\|WNT8A\|NFATC1\|ADCY6\|TGFB3\|CHEK2\|JAK1\|MYBL2\|PIK3CG\|PIK3CD\|EGR1\|ADCY9\|PRKACA\|VDAC1\|IL2\|CREBBP\|TBPL1\|TERT\|CDC23\|DVL1\|RRAS\| |
| Nicotine addiction | bta05033 | 7 | 0.028621565 | GRIN3B\|GABRB3\|GABRA1\|GABRA5\|GABRA6\|GABRG3\|GABRB2\| |
| PI3K-Akt signaling pathway | bta04151 | 25 | 0.029540128 | PIK3CD\|FGF17\|FGFR1\|ITGA1\|RPS6\|PPP2CA\|STK11\|JAK1\|TNR\|ITGA7\|FOXO3\|MAPK3\|ANGPT1\|FGF12\|PPP2R5C\|ITGA10\|FGF5\|EFNA2\|YWHAE\|GNB1\|IL2\|PIK3CG\|COL4A2\|PPP2R5D\|MTOR\| |
| Proteoglycans in cancer | bta05205 | 17 | 0.037355488 | MAPK3\|CD63\|WNT8A\|HPSE2\|PIK3CD\|PIK3CG\|PPP1R12B\|FGFR1\|CAMK2A\|PPP1R12A\|HOXD10\|PRKACA\|MTOR\|IL12B\|RPS6\|RRAS\|PPP1CC\| |
| Toxoplasmosis | bta05145 | 12 | 0.038201241 | MAPK3\|JAK1\|BIRC2\|TGFB3\|CIITA\|PIK3CD\|PIK3CG\|TAB2\|IRAK4\|GNAI2\|BIRC3\|IL12B\| |
| mRNA surveillance pathway | bta03015 | 10 | 0.042823111 | RNGTT\|PPP2R5C\|PPP2R5D\|CPSF1\|ETF1\|WDR82\|SSU72\|HBS1L\|PPP1CC\|PPP2CA\| |
| Progesterone-mediated oocyte maturation | bta04914 | 10 | 0.045525421 | MAPK3\|ADCY6\|PDE3B\|CDK1\|PIK3CG\|PIK3CD\|ADCY9\|GNAI2\|CDC23\|PRKACA\| |
| Neurotrophin signaling pathway | bta04722 | 12 | 0.045631177 | MAPK3\|BDNF\|PIK3CG\|PIK3CD\|CAMK2A\|NTRK3\|IRAK4\|FOXO3\|SH2B3\|YWHAE\|NTRK2\|KIDINS220\| |

**References**

Danecek P, Auton A, Abecasis GR, Albers CA, Banks ED, Depristo MA, Handsaker RE, Lunter G, Marth GT and Sherry STJB 2011. The variant call format and VCFtools. 27, 2156-2158.

Li H and Durbin RJb 2009. Fast and accurate short read alignment with Burrows–Wheeler transform. 25, 1754-1760.
